# Base-promoted lipase-catalyzed kinetic resolution of atropisomeric 1,1′-biaryl-2,2′-diols†

**DOI:** 10.1039/c8ra09070j

**Published:** 2019-01-09

**Authors:** Gamal A. I. Moustafa, Kengo Kasama, Koichi Higashio, Shuji Akai

**Affiliations:** Graduate School of Pharmaceutical Sciences, Osaka University 1-6 Yamadaoka Suita, Osaka 565-0871 Japan akai@phs.osaka-u.ac.jp; Department of Medicinal Chemistry, Faculty of Pharmacy, Minia University Minia 61519 Egypt

## Abstract

Herein we report a dramatic acceleration of the lipase-catalyzed kinetic resolution of atropisomeric 1,1′-biaryl-2,2′-diols by the addition of sodium carbonate. This result likely originates from the increased nucleophilicity of the phenolic hydroxyl group toward the acyl-enzyme intermediate. Under these conditions, various substituted *C*_2_-symmetric and non-*C*_2_-symmetric binaphthols and biphenols were efficiently resolved with ∼50% conversion in only 13–30 h with excellent enantioselectivity.

## Abstract 2

The addition of a stoichiometric amount of Na_2_CO_3_ dramatically accelerates the lipase-catalyzed kinetic resolution of a range of atropisomeric 1,1′-biaryl-2,2′-diols.

## Graphical abstract

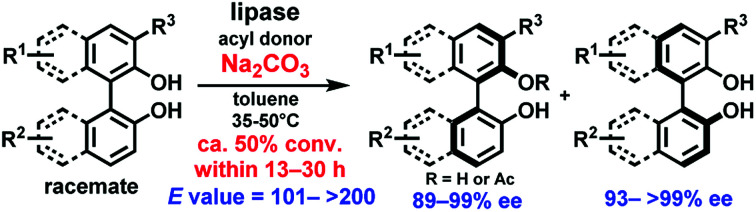

## Introduction

Axially chiral biaryls are ubiquitous structural motifs in various catalysts, natural products, and pharmaceuticals.^1^ In particular, optically active atropisomeric 1,1′-biaryl-2,2′-diols (*e.g.* 1,1′-bi-2-naphthols (BINOLs)) have been widely used as chiral ligands for asymmetric transformations and as privileged scaffolds for designing numerous important chiral catalysts/ligands.^2^ Consequently, the development of efficient enantioselective syntheses of 1,1′-biaryl-2,2′-diols has attracted considerable attention from various research groups.^3^ Although the enantioselective oxidative coupling of 2-naphthols using chiral transition metals is an atom-economical route, it is mainly applicable to homochiral binaphthols with specific substitution patterns.^4^ Apart from the kinetic resolution (KR) of racemic precursors *via* non-enzymatic routes,^5^ the lipase-catalyzed KR of racemic atropisomeric 1,1′-biaryl-2,2′-diols has been intensively studied.^6^ At the core of this, racemic BINOL and its derivatives have been resolved *via* either hydrolytic (in aqueous media)^7^ or acylative (in non-aqueous media) approaches.^8,9^ In the latter cases, the acylative KR of racemic BINOLs using a lipoprotein lipase from *Pseudomonas* sp. requires long reaction times (3–14 days) to afford the corresponding (*R*)-mono-esters in 32–53% yields with 90–95% ee and the recovery of (*S*)-BINOLs with 55–89% ee (*E* values:^10,11^ 41–117).^8*a*,*b*^ We believe that acceleration of the resolution process and the expansion of substrate scope are highly demanded to establish a more practical KR. Notably, to the best of our knowledge, none of the existing enzymatic KR protocols has been applied to *C*_1_-symmetric, *i.e.* non-*C*_2_-symmetric biaryl diols. Therefore, we studied the development of a modular KR that enhances the enzymatic transesterification and also covers a broader range of substituted biaryl diols. In this article, we show that the addition of sodium carbonate dramatically accelerates the KR for a broad range of *C*_1_-symmetric and *C*_2_-symmetric atropoisomeric 1,1′-biaryl-2,2′-diols, while maintaining excellent enantiocontrol.

## Results and discussion

We began this study by investigating the KR parameters of BINOL (±)-1a as a model substrate using either vinyl acetate (10 equiv.) or isopropenyl acetate (10 equiv.) as an acyl donor and the commercially available immobilized *Pseudomonas* sp. lipoprotein lipase (Toyobo LIP301). As shown in Table 1, only 4–30% conversions were achieved after 24 h at the temperature of 35 or 50 °C with lipase loading of 1–3 w/w in spite of the high enantioselectivity (*E* value = 58–>200) (entries 1–6). To realize the requisite rate enhancement, we envisioned that the addition of some basic additives would promote the reaction by enhancing the nucleophilicity of the phenolic hydroxyl group towards an acyl-enzyme intermediate. To our delight, the addition of Na_2_CO_3_ (1.5 mol equiv.) at 35 °C dramatically increased the acylation rate while maintaining a substantial level of enantio-discrimination (*E* value = >200) giving the monoacetate (*R*)-2a in 50% NMR yield with 99% ee and the recovered (*S*)-1a in 50% NMR yield with 98% ee (entry 8). This result was reproduced on a larger scale (0.5 mmol of (±)-1a) leading to perfect conversion after 24 h with the formation of (*R*)-2a (51% isolated yield, 96% ee) and (*S*)-1a (48% isolated yield, >99% ee) (*E* = >200) (entry 9). Reducing the amount of Na_2_CO_3_ to 0.3 mol equiv. resulted in only 8% conversion after 24 h (entry 10). We could reduce the lipase loading further to 1 w/w and the acyl donor to 5 equiv. at 50 °C to afford both (*R*)-2a and (*S*)-1a in high optical purities with 50% conversion (entries 11–12) (*cf.* previous studies required 4 w/w lipase and 20 equiv. of vinyl acetate^8*a*,*b*^). The use of isopropenyl acetate led to a similar rate enhancement with high enantioselectivity (*E* value = 149) (entry 14).^12^ In all cases, the formation of diacetate 3a was not observed.

**Table 1:** Optimization of the lipase-catalyzed KR of BINOL (±)-1a^a^

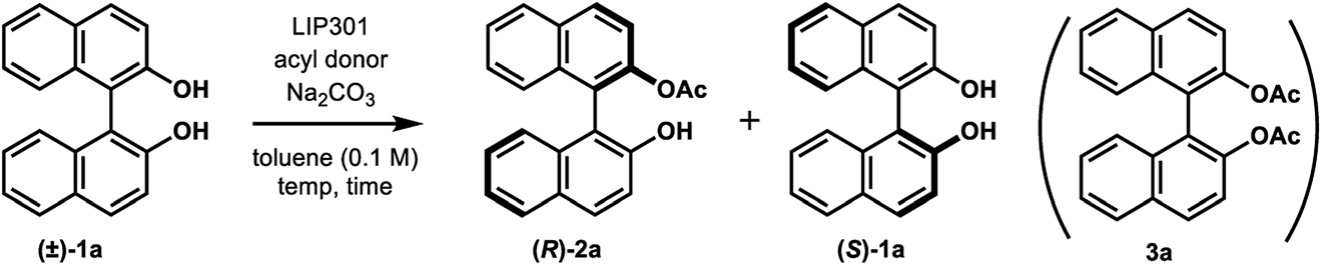									
Entry	Na_2_CO_3_ (mol equiv.)	LIP301^b^ (w/w)	Acyl donor (equiv.)	Temp (°C)	Time (h)	Conv. (%)^c^	(*R*)-2a (% ee)^d^	(*S*)-1a (% ee)^d^	*E* e
1	0	1	Vinyl acetate (10)	35	24	4	>99	4	>200
2	0	3	Vinyl acetate (10)	35	24	7	>99	8	>200
3	0	3	Vinyl acetate (10)	50	24	30	95	40	58
4	0	1	Isopropenyl acetate (10)	35	24	7	98	7	106
5	0	3	Isopropenyl acetate (10)	35	24	8	98	9	108
6	0	3	Isopropenyl acetate (10)	50	24	16	98	18	118
7	1.5	1	Vinyl acetate (10)	35	24	36	99	56	>200
8	1.5	3	Vinyl acetate (10)	35	24	50	99	98	>200
9^f^	1.5	3	Vinyl acetate (10)	35	24	51	96 (51% yield)^g^	>99 (48% yield)^g^	>200
10	0.3	3	Vinyl acetate (10)	35	24	8	99	8	>200
11	1.5	1	Vinyl acetate (10)	50	24	50	95	94	139
12	1.5	1	Vinyl acetate (5)	50	24	50	96	95	138
13	1.5	1	Isopropenyl acetate (10)	35	24	33	98	49	161
14	1.5	3	Isopropenyl acetate (10)	35	17	50	98	94	149

a Except for entry 9, the screening was done using *ca.* 0.03 mmol of (±)-1a.

b Commercially available immobilized *Pseudomonas* sp. lipoprotein lipase (Toyobo LIP301).

c Calculated based on the optical purities of (*R*)-2a and (*S*)-1a, see ref. 10 and 11*a*.

d Determined by chiral HPLC.

e For *E* value, see ref. 10 and 11*a*.

f Reaction conducted using 0.5 mmol of (±)-1a.

g Isolated yields.

We also examined the effects of other types of organic and inorganic bases at 35 °C for 24 h (Table 2). Both the conversions and enantioselectivities dropped off sharply when pyridine or Hünig's base were added (entries 2 and 3). Although 48% conversion was attained by adding triethylamine, the enantioselectivity was very poor (entry 4). The conversions did not exceed 13% when Li_2_CO_3_, MgCO_3_, and CaCO_3_ were examined despite the high enantioselectivity (entries 5, 7, and 9, respectively). Very low conversion and poor enantioselectivity were obtained upon using Cs_2_CO_3_ (entry 10). The use of K_2_CO_3_ resulted in 46% conversion with moderate enantiocontrol (*E* = 43) (entry 8). Other sodium salts like NaHCO_3_ and Na_2_HPO_4_ led to poor conversions after 24 h, albeit in the high *E* values (>200) (entries 11, 12 and 14). Although Na_3_PO_4_ afforded a 52% conversion (entry 13), the resultant enantioselectivity (*E* = 99) was inferior to that obtained with Na_2_CO_3_ (*E* = >200, entry 6).

**Table 2:** Effect of basic additives on the KR of (±)-1a

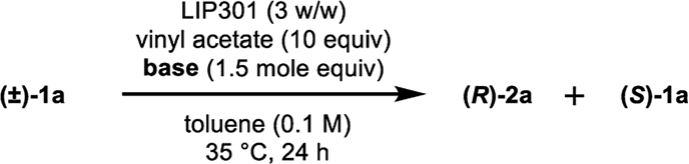					
Entry	Base	Conv. (%)^a^	(*R*)-2a (% ee)^b^	(*S*)-1a (% ee)^b^	*E*
1^c^	None	7	>99	8	>200
2	Pyridine	13	91	13	24
3	EtN(iPr)_2_	24	60	19	5
4^d^	Et_3_N	48	52	48	5
5	Li_2_CO_3_	4	99	4	>200
6^e^	Na_2_CO_3_	50	99	98	>200
7	MgCO_3_	13	99	15	>200
8^d^	K_2_CO_3_	46	90	75	43
9	CaCO_3_	5	99	5	>200
10^d^	Cs_2_CO_3_	10	18	2	1.5
11	NaHCO_3_	12	99	13	>200
12^f^	NaHCO_3_	17	99	20	>200
13	Na_3_PO_4_	52	90	99	99
14	Na_2_HPO_4_	20	99	20	>200

a Calculated based on the optical purities of (*R*)-2a and (*S*)-1a, see ref. 10 and 11*a*.

b Determined by chiral HPLC.

c From Table 1, entry 2.

d The formation of diacetate 3a was not observed under these conditions.

e From Table 1, entry 8.

f 3 mol equiv. of NaHCO_3_ was added.

We found that the poor enantioselectivity caused by triethylamine, K_2_CO_3_, or Cs_2_CO_3_, in comparison to the excellent enantiocontrol in the case of Na_2_CO_3_, was due to the competitive non-enzymatic acetylation. Thus, a similar reaction of (±)-1a in the presence of these bases, while omitting the lipase, resulted in the formation of a mixture of (±)-2a and the diacetate (±)-3a (Table 3, entries 1, 3, and 4), while the formation of diacetate 3a was not detected in the presence of lipase (Table 2, entries 4, 8, and 10). On the other hand, Na_2_CO_3_ did not promote any non-enzymatic acylation (entry 2) and has been identified as the ideal base for the enzymatic transformation in terms of both reaction rate and enantioselectivity.

**Table 3:** Testing the non-enzymatic acetylation of (±)-1a in the presence of bases

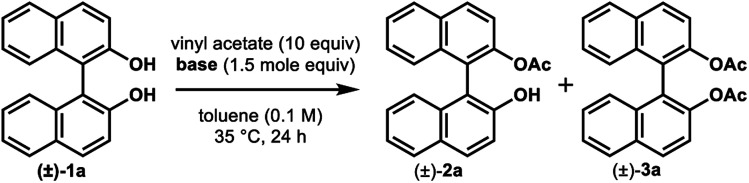				
Entry	Base	1a (Yield%)^a^	2a (Yield%)^a^	3a (Yield%)^a^
1	Et_3_N	48	48	4
2	Na_2_CO_3_	100	0	0
3	K_2_CO_3_	0	2	98
4	Cs_2_CO_3_	25	56	19

a Determined by ^1^H NMR analysis of the crude mixture.

Under the optimal conditions obtained using Na_2_CO_3_ as the base, various substituted *C*_2_-symmetric biaryl diols (±)-1b–e were successfully resolved with ∼50% conversion in only 16–30 h with excellent enantioselectivity (*E* = > 100) (Table 4, entries 1, 2, 4 and 5). Particularly, 6,6′-dimethoxy-1,1′-biphenol (1d) underwent highly enantioselective KR with ∼50% conversion after 24 h using lower amounts of lipase (2 w/w) and vinyl acetate (5 equiv.), thus offering an advantage over the reported enzymatic method that required three days using 4 w/w of lipase PS-IM and 1.5 equiv. of vinyl acetate to achieve 41% conversion.^8*c*^ Notably, the reaction of 1c proceeded very slowly in absence of Na_2_CO_3_; only 10% yield of (*R*)-2c was obtained after 24 h (entry 3), which again confirms the significance of Na_2_CO_3_ in accelerating the resolution process as seen in entry 2. All these reactions led only to the formation of monoacetates (*R*)-2 without any detectable amount of the corresponding diacetates 3. On the other hand, 3,3′-dibromo-1,1′-binaphthol (1f) did not react at all (entry 6); similar results were obtained using different types of immobilized lipases under different conditions probably due to the steric bulkiness imparted by the substituents at the 3,3′-positions that may retard the acyl transfer from the acyl-enzyme intermediate to the substrate.

**Table 4:** KR of *C*_2_-symmetric biaryl diols (±)-1b–f^a^

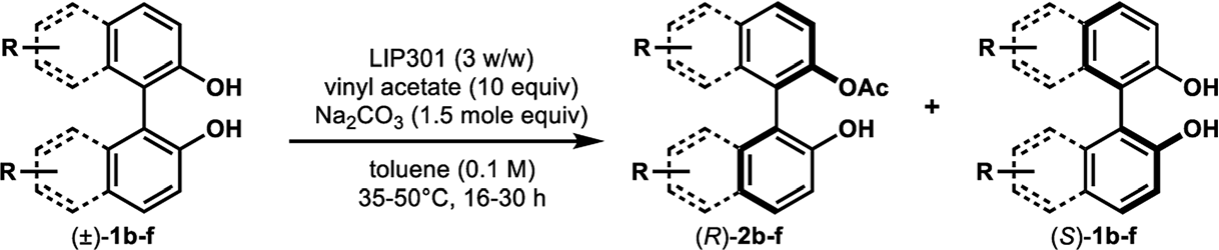							
Entry	Substrate	Temp. and time	(*R*)-2b–g		(*S*)-1b–g		*E*
			Isolated yield (%)	% ee^b^	Isolated yield (%)	% ee^b^	
1	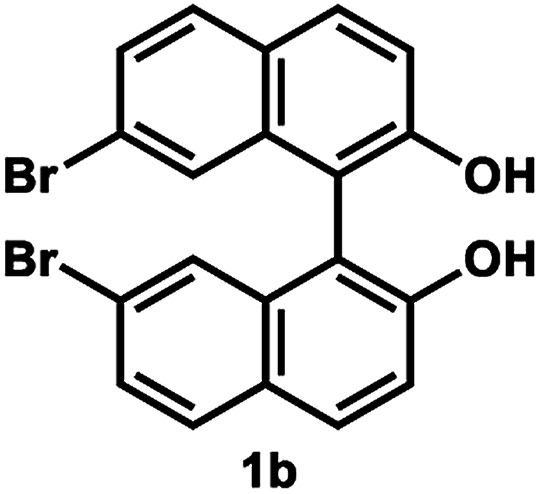	50 °C, 29 h	49	95	50	99	>200
2	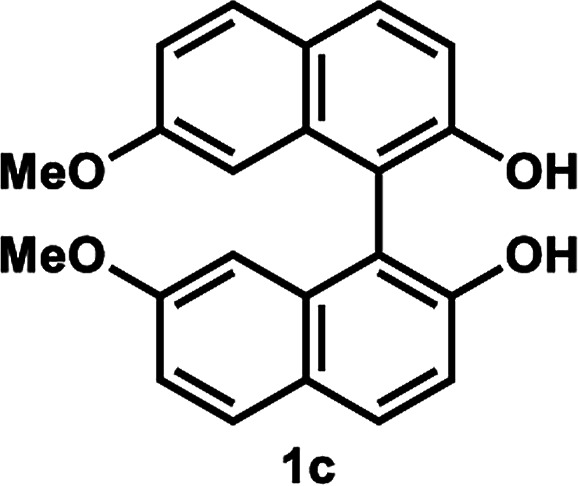	50 °C, 30 h	54	97	43	99	>200
3^c^		50 °C, 30 h	10	99	86	11	>200
4^d^	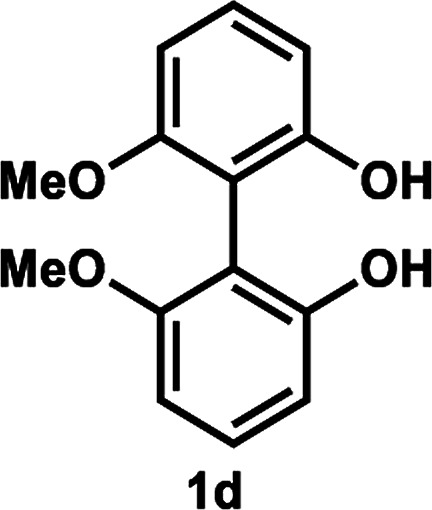	35 °C, 24 h	53	89	47	>99	101
5^d^^,^^e^	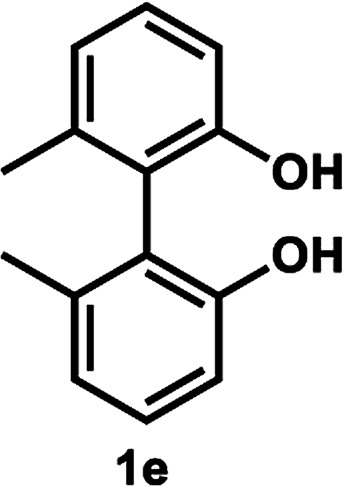	35 °C, 16 h	52	95	47	97	165
6	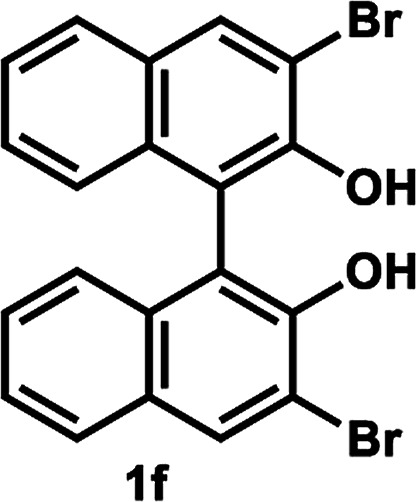	50 °C, 24 h	No reaction				

a Reaction was conducted using (±)-1b–f (0.1 mmol).

b Determined by chiral HPLC.

c In absence of Na_2_CO_3_.

d 2 w/w LIP301 and 5 equiv. vinyl acetate were used.

e i-Pr_2_O was used instead of toluene.

The successful application of our method to *C*_1_-symmetric biaryl diols (±)-1g–k is noteworthy (Table 5). Although they produced a mixture of regioisomeric acetates (*R*)-2g–k, we found that each isomer has the same (*R*) absolute configuration and high optical purity. For instance, two acetates (*R*)-2i were obtained; each of them with 91% ee, and their mixture was subjected to methanolysis to give a single product (*R*)-1i with 91% ee (Table 5, entry 3). In a similar manner, simple methanolysis of the mixtures of regioisomeric acetates (*R*)-2g–h and (*R*)-2j–k afforded (*R*)-diols (*R*)-1g–h and (*R*)-1i–k with complete retention of optical purity (entries 1, 2, 5, and 6). While the reported asymmetric oxidative cross coupling of 2-naphthols afforded (*R*)-1h with a maximum optical purity of 72% ee,^4*e*^ the optimized KR protocol discussed here afforded much higher optical purities for both (*R*)-1h (95% ee) and (*S*)-1h (>99%) (entry 2). Importantly, the acetylation of 1i did not proceed in absence of Na_2_CO_3_, reconfirming the necessity to add this base to promote the KR (entry 4). Notably, the reaction worked well for substrates possessing only one substituent at the 3-position (Table 5, entries 3, 5, and 6).

**Table 5:** KR of non-*C*_2_-symmetric biaryl diols (±)-1g–k^a^

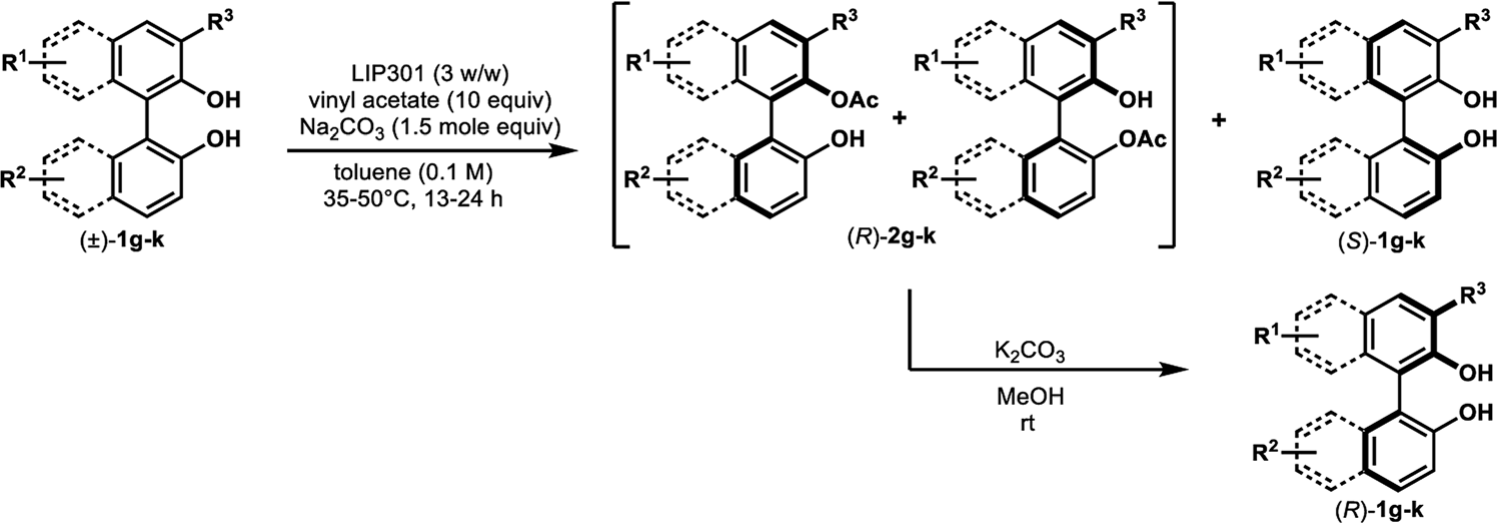							
Entry	Substrate	Temp. and time	(*R*)-1g–k		(*S*)-1g–k		*E*
			Isolated yield (%)^b^	% ee^b^	Isolated yield (%)	% ee^c^	
1^d^	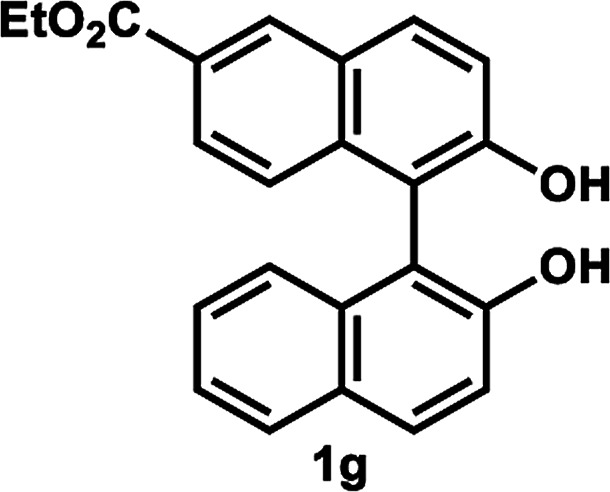	35 °C, 14 h	48	95	51	93	126
2	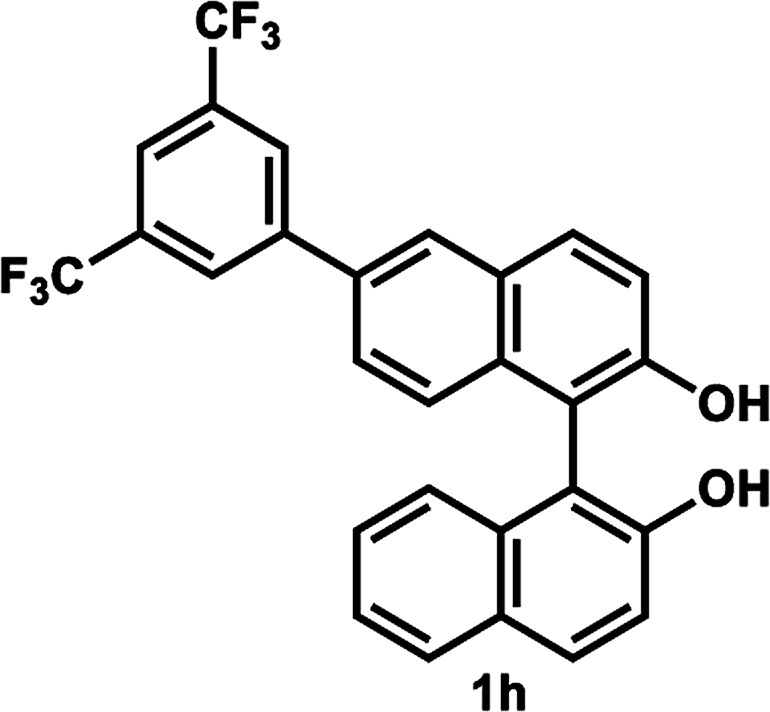	35 °C, 24 h	47	95	40	>99	>200
3^d^^,^^e^	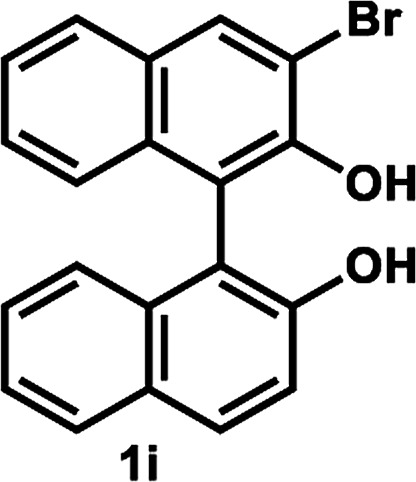	35 °C, 13 h	48	91	44	>99	114
4^f^		35 °C, 24 h	No reaction				
5	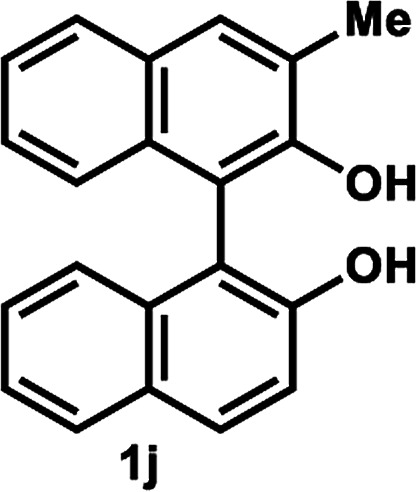	50 °C, 24 h	45	92	41	99	126
6	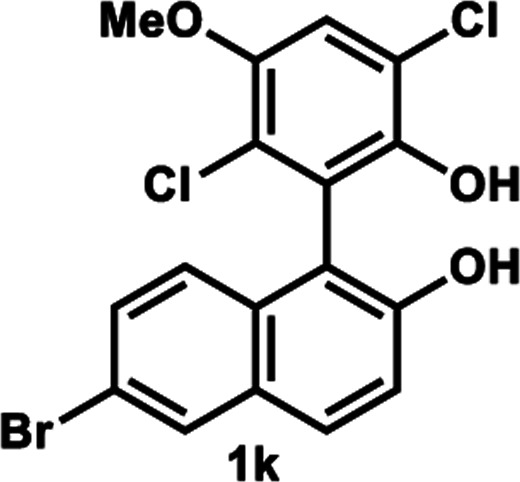	50 °C, 14 h	43	96	44	99	>200

a Reaction was conducted using (±)-1g–k (0.1 mmol).

b Obtained by methanolysis of a mixture of two regioisomers (*R*)-2 (for details, see Experimental section), and the optical purity was determined by chiral HPLC after methanolysis.

c Determined by chiral HPLC.

d Using 3 mol equiv. Na_2_CO_3_.

e Using 4 w/w LIP301.

f In absence of Na_2_CO_3_.

We further examined the applicability of our method to the biaryl methanol derivative 4a (Table 6). However, the addition of Na_2_CO_3_ in this case was deleterious to the enantioselectivity, affording the optically inactive product 5a (2% ee, 100% NMR yield) in which the primary hydroxyl group is acetylated (Table 6, entry 1). The 100% conversion is probably due to a concomitant non-enzymatic acetylation of the primary alcohol.^13^ The omission of Na_2_CO_3_ dramatically improved the enantioselectivity (*E* = 34) (entry 2). Lowering the reaction temperature to 25 °C afforded better *E* value (65) (entry 3); such high enantioselectivity was not realized in previously reported lipase-catalyzed resolution.^14^ The protocol was also applied successfully to diol 4b, resulting in high enantioselectivity (*E* = 127) with 50% conversion after 32 h (entry 4).^15^

**Table 6:** KR of biaryl methanol derivatives (±)-4a–b^a^

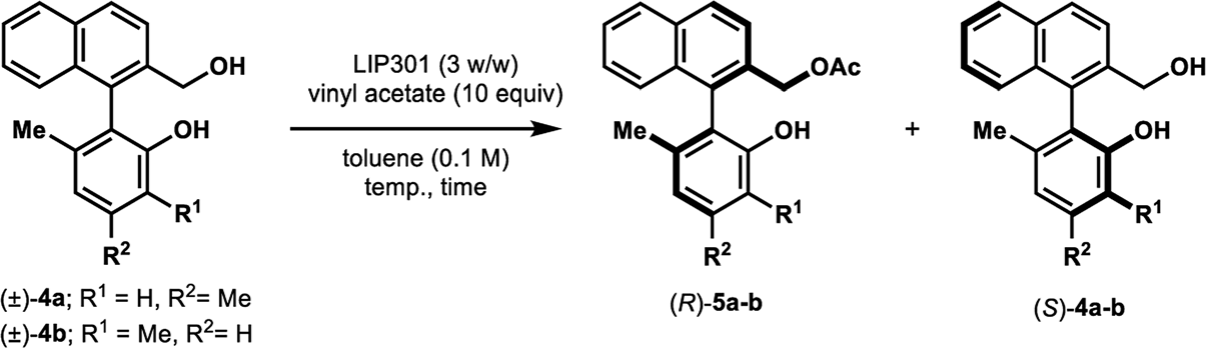								
Entry	Substrate	Na_2_CO_3_ (mol equiv.)	Temp. (°C)	Time (h)	Conv. (%)^b^	(*R*)-5a–b^c^	(*S*)-4a–b^c^	*E*
1	4a	1.5	35	24	100	2% ee, quant.	ND	ND
2	4a	0	35	24	57	68% ee, 57% NMR yield	>99% ee, 43% NMR yield	34
3	4a	0	25	48	52	88% ee, 55% isolated yield	97% ee, 43% isolated yield	65
4	4b	0	25	32	50	93% ee, 49% isolated yield	98% ee, 51% isolated yield	127

a Reaction was conducted using (±)-4a–b (0.1 mmol).

b Determined by ^1^H NMR analysis of the crude mixture.

c Optical purity was determined by chiral HPLC. ND: not determined.

Based on the results that produced acceleration of the reaction by the addition of Na_2_CO_3_ in the case of phenolic substrates 1a–e and 1g–k, we considered the lipase-mediated catalytic cycle for the enantioselective esterification. In principle, the KR cycle begins with the transfer of acyl group from the acyl donor (*e.g.* vinyl acetate) to the hydroxyl group of serine in the reactive site of lipase to form an acyl-enzyme intermediate,^16^ which is subsequently attacked by the phenolic hydroxyl group of 1 in an enantioselective manner. Since *Pseudomonas* sp. lipoprotein lipase has been used in the KR of secondary alcohols without Na_2_CO_3_ leading to ∼50% conversion in shorter time,^17^ the formation of the acyl-enzyme intermediate is probably not the rate-limiting step. Instead, steric hindrance in the biaryl skeleton of 1 may decelerate the acyl transfer to 1. Moreover, the phenolic hydroxyl groups in 1 are more acidic with less nucleophilicity toward the acyl-enzyme than the alcoholic hydroxyl groups. This assumption was supported by the results obtained for alcohols 4, in which the addition of Na_2_CO_3_ was not required and the acylation occurred on their primary hydroxyl groups with perfect chemoselectivity.

It is worth mentioning that the dynamic kinetic resolution (DKR) of secondary alcohols using a combination of ruthenium complexes and lipases has often been performed in the presence of Na_2_CO_3_.^18^ Similarly, we also noted the importance of Na_2_CO_3_ addition in our recent chemoenzymatic DKR of certain 2,2′-dihydroxy-1,1′-biaryls using a combination of a ruthenium complex and LIP301;^9^ however, no clear explanation of the role of such additive has been proposed until now. Herein, we have partially elucidated the impact of this additive in enhancing the KR rate. In addition, the optical purities (76–89% ee) of the compounds 1b, 1c, 1g, and 1i, obtained by our DKR protocol,^9^ have been improved to 91–97% ee in this study by modification of the KR conditions.

## Conclusions

In contrast to the former lipase-catalyzed KRs of atropisomeric 2,2′-dihydroxy-1,1′-biaryls that required long reaction times (3–14 days) and were of limited scope,^8^ we have improved the rate of enzymatic KR and expanded its applicability to a range of this class of compounds, such as 1a–e and 1g–k, with ∼50% conversions within 30 h and with excellent enantioselectivity (*E* = 101–>200). The rate enhancement, realized by the addition of Na_2_CO_3_, is probably due to an improvement in the nucleophilicity of the phenolic hydroxyl group of 1 towards an acyl-enzyme intermediate. Since there is only one report describing an organocatalyzed resolution of non-*C*_2_-symmetric axially chiral dihydroxy biaryl 1i with moderate enantioselectivity (*S* value = 38),^5*a*^ our method shows broader applicability to non-*C*_2_-symmetric dihydroxy biaryls with higher enantioselectivities. The finding described here will also expand the efficiency of DKR of atropisomers particularly when combined with efficient racemization catalysts. Detailed mechanistic investigation of the effect of Na_2_CO_3_ is in progress in our laboratory.

## Experimental

### General considerations

Melting points were determined on a Yanagimoto Melting Point Apparatus and are uncorrected. Infrared (IR) absorption spectra were recorded on a SHIMADZU FTIR-8400S spectrophotometer. ^1^H and ^13^C NMR spectra were measured on a JEOL JNM-ECA500 (^1^H: 500 MHz, ^13^C: 125 MHz) or JEOL JNM-ECS400 (^1^H: 400 MHz, ^13^C: 100 MHz) instrument with chemical shifts reported in *δ* (ppm) relative to the residual nondeuterated solvent signal for ^1^H (CHCl_3_: *δ* = 7.26 ppm) and relative to the deuterated solvent signal for ^13^C (CDCl_3_: *δ* = 77.0 ppm). The mass spectra (MS) were measured on a JEOL JMS-S3000 (MALDI) with TOF mass analyser. HPLC analyses were carried out using a JASCO LC-2000Plus system (HPLC pump: PU-2080, UV detector: MD-2018) equipped with a Daicel CHIRALPAK IC-3, CHIRALPAK AD-3, CHIRALCEL OD-3, CHIRALCEL OZ-3 or CHIRALPAK IE column; each with a size of 4.6 mm × 250 mm. Optical rotations were measured on a JASCO P-1020 polarimeter. Gel permeation chromatography was carried out on LaboACE LC-5060 with JAIGEL-2HR columns (Japan Analytical Industry). The lipase from *Pseudomonas* sp. (TOYOBO lipoprotein lipase GradeIII LPL-311) immobilized on Hyflo Super-Cel (commercial name: TOYOBO LIP301) was gifted from TOYOBO CO., LTD. Kanto silica gel 60N was used for column chromatography. In general, the reactions were carried out in anhydrous solvents. Racemic substrates 1b,^19^1c,^20^1d,^21^1e,^22^1f,^23^1g,^9^1h,^4*e*^1i,^24^1j,^23^1k,^25^4a,^14^ and 4b^14^ were prepared according to reported procedure.

### Kinetic resolution of (±)-1a (Table 1, entry 9)

To a stirred solution of (±)-1a (143 mg, 0.50 mmol) and vinyl acetate (0.46 mL, 5.0 mmol) in anhydrous PhCH_3_ (5.0 mL, 0.10 M) were added LIP301 (0.43 g, 3 w/w) and Na_2_CO_3_ (80 mg, 0.75 mmol). After being stirred for 24 h at 35 °C, the reaction mixture was filtered through a Celite pad. The Celite pad was washed with EtOAc and the combined filtrate was evaporated *in vacuo*. The residue was purified by flash column chromatography (hexanes/EtOAc = 8 : 1) to give ester (*R*)-2a (84 mg, 51% yield, 96% ee) and recovered (*S*)-1a (68 mg, 48% yield, >99% ee).

#### (*R*)-2′-Hydroxy-(1,1′-binaphthalen)-2-yl acetate (*R*)-2a

White solid; mp 56–57 °C (lit.^9^ mp 55–58 °C); [*α*]^22^_D_ + 84.7 (*c* 1.01, CHCl_3_) (lit.^9^ [*α*]^20^_D_ + 76 (*c* 1.08, CHCl_3_) for (*R*)-2a with 99% ee); ^1^H-NMR (500 MHz, CDCl_3_) *δ* 8.07 (d, *J* = 8.5 Hz, 1H), 7.97 (d, *J* = 8.5 Hz, 1H), 7.91 (d, *J* = 8.5 Hz, 1H), 7.86 (d, *J* = 8.5 Hz, 1H), 7.51 (td, *J* = 7.5, 1.5 Hz, 1H), 7.40 (d, *J* = 9.0 Hz, 1H), 7.31–7.36 (m, 3H), 7.23–7.26 (m, 3H), 7.03 (d, *J* = 7.5 Hz, 1H), 5.20 (s, 1H), 1.87 (s, 3H). The spectroscopic data are in good agreement with those reported.^9^ Its optical purity (96% ee) was determined by HPLC analysis at 20 °C using a CHIRALPAK IC-3 column (hexanes/2-propanol = 95 : 5; flow rate: 1.0 mL min^−1^; retention times: 8.0 min (*R*), 10.7 min (*S*)).

#### (*S*)-1,1′-Bi-2-naphthol (*S*)-1a

White solid; mp 205–207 °C (lit.^26^ mp 207–210 °C); [*α*]^22^_D_ − 34.7 (*c* 1.02, THF) (lit.^26^ [*α*]^21^_D_ − 34 (*c* 1.00, THF) for (*S*)-1a with 99% ee); ^1^H-NMR (500 MHz, CDCl_3_) *δ* 7.99 (d, *J* = 8.5 Hz, 2H), 7.90 (d, *J* = 8.0 Hz, 2H), 7.36–7.40 (m, 4H), 7.30–7.33 (m, 2H), 7.16 (d, *J* = 8.5 Hz, 2H), 5.06 (s, 2H). The spectroscopic data are in good agreement with those reported.^27^ Its optical purity (>99% ee) was determined by HPLC analysis at 20 °C using a CHIRALPAK IC-3 column (hexanes/2-propanol = 95 : 5; flow rate: 1.0 mL min^−1^; retention times: 14.5 min (*R*), 20.8 min (*S*)).

### Kinetic resolution of *C*_2_-symmetric biaryl diols (±)-1b–f (Table 4)

#### Representative procedure A (Table 4, entry 2)

To a stirred solution of (±)-1c (34.6 mg, 0.10 mmol) and vinyl acetate (93 μL, 1.0 mmol) in anhydrous PhCH_3_ (1.0 mL, 0.10 M) were added LIP301 (133 mg, 3 w/w) and Na_2_CO_3_ (15.9 mg, 0.15 mmol). After being stirred for 30 h at 50 °C, the reaction mixture was filtered through a Celite pad. The Celite pad was washed with EtOAc and the combined filtrate was evaporated *in vacuo*. The residue was purified by preparative thin layer chromatography (PTLC) (PhCH_3_/EtOAc = 8 : 1) to give ester (*R*)-2c (21 mg, 54% yield, 97% ee) and recovered (*S*)-1c (14.8 mg, 43% yield, 99% ee).

##### (*R*)-2′-Hydroxy-7,7′-dimethoxy-(1,1′-binaphthalen)-2-yl acetate (*R*)-2c

White solid; mp 47–49 °C; [*α*]^20^_D_ − 40.8 (*c* 0.24, CHCl_3_); ^1^H-NMR (400 MHz, CDCl_3_) *δ* 8.00 (d, *J* = 9.0 Hz, 1H), 7.88 (d, *J* = 9.0 Hz, 1H), 7.83 (d, *J* = 9.0 Hz, 1H), 7.76 (d, *J* = 9.0 Hz, 1H), 7.28 (d, *J* = 1.0 Hz, 1H), 7.17–7.20 (m, 2H), 7.01 (dd, *J* = 9.0, 3.0 Hz, 1H), 6.59 (d, *J* = 2.0 Hz, 1H), 6.40 (d, *J* = 2.0 Hz, 1H), 5.17 (s, 1H), 3.58 (s, 3H), 3.57 (s, 3H), 1.85 (s, 3H); ^13^C-NMR (100 MHz, CDCl_3_) *δ* 170.3, 158.9, 158.3, 152.1, 148.6, 134.8, 134.7, 130.4, 130.1, 129.8, 129.5, 127.6, 124.3, 121.7, 119.3, 118.8, 115.50, 115.46, 113.2, 104.0, 103.6, 55.1, 55.0, 20.4; IR (CHCl_3_) *ν* 3545, 1755 cm^−1^; HRMS (MALDI) *m*/*z* calcd for C_24_H_20_O_5_ [M]^+^: 388.1305, found: 388.1304. Its optical purity (97% ee) was determined by HPLC analysis at 20 °C using a CHIRALPAK IC-3 column (hexanes/2-propanol = 92.5 : 7.5; flow rate: 1.0 mL min^−1^; retention times: 8.6 min (*R*), 12.4 min (*S*)).

##### (*S*)-7,7′-Dimethoxy-1,1′-bi-2-naphthol (*S*)-1c

White solid; mp 62–63 °C; [*α*]^20^_D_ + 152.5 (*c* 0.31, CHCl_3_) (lit.^28^ [*α*]^22^_D_ + 122.3 (*c* 1.0, CHCl_3_) for (*S*)-1c with 94% ee); ^1^H-NMR (400 MHz, CDCl_3_) *δ* 7.88 (d, *J* = 9.0 Hz, 2H), 7.79 (d, *J* = 9.0 Hz, 2H), 7.22 (d, *J* = 9.0 Hz, 2H), 7.04 (dd, *J* = 9.0, 3.0 Hz, 2H), 6.48 (d, *J* = 3.0 Hz, 2H), 5.07 (s, 2H), 3.58 (s, 6H). The spectroscopic data are in good agreement with those reported.^28^ Its optical purity (99% ee) was determined by HPLC analysis at 20 °C using a CHIRALPAK IC-3 column (hexanes/2-propanol = 80 : 20; flow rate: 1.0 mL min^−1^; retention times: 7.3 min (*R*), 9.4 min (*S*)).

#### Table 4, entry 1

Following the above-mentioned procedure A for KR, (±)-1b (44.4 mg, 0.10 mmol) was converted to (*R*)-2b (24 mg, 49% yield, 95% ee) and (*S*)-1b (22 mg, 50% yield, 99% ee), after purification of the crude reaction mixture by PTLC (PhCH_3_/EtOAc = 8 : 1).

##### (*R*)-7,7′-Dibromo-2′-hydroxy-(1,1′-binaphthalen)-2-yl acetate (*R*)-2b

White solid; mp 65–67 °C; [*α*]^23^_D_ − 75.7 (*c* 0.63, CHCl_3_); ^1^H-NMR (500 MHz, CDCl_3_) *δ* 8.05 (d, *J* = 9.0 Hz, 1H), 7.89 (d, *J* = 9.0 Hz, 1H), 7.85 (d, *J* = 9.0 Hz, 1H), 7.73 (d, *J* = 9.0 Hz, 1H), 7.61 (dd, *J* = 9.0, 2.0 Hz, 1H), 7.33–7.44 (m, 4H), 7.13 (d, *J* = 2.0 Hz, 1H), 5.25 (s, 1H), 1.87 (s, 3H); ^13^C-NMR (100 MHz, CDCl_3_) *δ* 170.3, 152.6, 148.9, 134.54, 134.50, 131.1, 130.73, 130.68, 130.03, 130.01, 129.8, 127.4, 127.2, 126.2, 122.4, 122.2, 121.9, 121.5, 118.9, 112.7, 20.4 (two peaks are overlapped); IR (CHCl_3_) *ν* 3529, 1754 cm^−1^; HRMS (MALDI) *m*/*z* calcd for C_22_H_12_O_3_Na^79^Br_2_ [M + Na]^+^: 506.9202, found: 506.9198. Its optical purity (95% ee) was determined by chiral HPLC analysis at 20 °C using a CHIRALPAK IC-3 column (hexanes/2-propanol) = 97.5 : 2.5; flow rate: 1.0 mL min^−1^; retention times: 8.3 min for (*R*)-2b, 11.4 min for (*S*)-2b.

##### (*S*)-7,7′-Dibromo-1,1′-bi-2-naphthol (*S*)-1b

White solid; mp 136–138 °C; [*α*]^23^_D_ + 263 (*c* 0.75, CHCl_3_) (lit.^28^ [*α*]^21^_D_ + 129.1 (*c* 1.8, CHCl_3_) for (*S*)-1b with 73% ee); ^1^H-NMR (500 MHz, CDCl_3_) *δ* 7.95 (d, *J* = 9.0 Hz, 2H), 7.77 (d, *J* = 9.0 Hz, 2H), 7.48 (dd, *J* = 9.0, 2.0 Hz, 2H), 7.38 (d, *J* = 9.0 Hz, 2H), 7.23 (d, *J* = 2.0 Hz, 2H), 5.04 (s, 2H). The spectroscopic data are in good agreement with those reported.^28^ Its optical purity (99% ee) was determined by chiral HPLC analysis at 20 °C using a CHIRALPAK IC-3 column (hexanes/2-propanol = 97.5 : 2.5; flow rate: 1.0 mL min^−1^; retention times: 29.2 min for (*R*)-1b; 31.6 min for (*S*)-1b).

#### Table 4, entry 4

Following the above-mentioned procedure A for KR, (±)-1d (24.6 mg, 0.10 mmol) was converted to (*R*)-2d (15.0 mg, 53% yield, 89% ee) and (*S*)-1d (12.0 mg, 47% yield, >99% ee), after purification of the crude reaction mixture by flash column chromatography (CH_2_Cl_2_/Et_2_O = 20 : 1).

##### (*R*)-2′-Hydroxy-6,6′-dimethoxy-(1,1′-biphenyl)-2-yl acetate (*R*)-2d

Colorless oil; [*α*]^21^_D_ + 93.4 (*c* 0.63, CHCl_3_) (lit.^8*c*^ [*α*]^20^_D_ + 91.2 (*c* 0.70, CHCl_3_) for (*R*)-2d with 98% ee); ^1^H-NMR (400 MHz, CDCl_3_) *δ* 7.42 (t, *J* = 8.0 Hz, 1H), 7.24 (t, *J* = 8.0 Hz, 1H), 6.92–6.94 (m, 1H), 6.83–6.85 (m, 1H), 6.66 (d, *J* = 8.0 Hz, 1H), 6.55 (dd, *J* = 8.0, 1.0 Hz, 1H), 5.01 (s, 1H), 3.78 (s, 3H), 3.71 (s, 3H), 1.96 (s, 3H). The spectroscopic data are in good agreement with those reported.^8*c*^ Its optical purity (89% ee) was determined by HPLC analysis at 20 °C using a CHIRALPAK IC-3 column (hexanes/2-propanol = 92 : 8; flow rate: 1.0 mL min^−1^; retention times: 29.1 min (*R*), 32.2 min (*S*)).

##### (*S*)-2,2′-Dihydroxy-6,6′-dimethoxy-1,1′-biphenyl (*S*)-1d

White solid; mp 138–140 °C; [*α*]^21^_D_ − 102 (*c* 0.64, CHCl_3_) (lit.^21^ [*α*]^20^_D_ − 144 (*c* 0.77, CHCl_3_) for (*S*)-1d with 98.6% ee); ^1^H-NMR (400 MHz, CDCl_3_) *δ* 7.31 (t, *J* = 8.0 Hz, 2H), 6.72 (d, *J* = 8.0 Hz, 2H), 6.62 (d, *J* = 8.0 Hz, 2H), 5.05 (s, 2H), 3.77 (s, 6H). The spectroscopic data are in good agreement with those reported.^21^ Its optical purity (>99% ee) was determined by HPLC analysis at 20 °C using a CHIRALPAK IC-3 column (hexanes/2-propanol = 85 : 15; flow rate: 1.0 mL min^−1^; retention times: 12.9 min (*R*), 18.9 min (*S*)).

#### Table 4, entry 5

Following the above-mentioned procedure A for KR, (±)-1e (21.4 mg, 0.10 mmol) was converted to (*R*)-2e (13.5 mg, 52% yield, 95% ee) and (*S*)-1e (10.1 mg, 47% yield, 97% ee), after purification of the crude reaction mixture by flash column chromatography (hexanes/EtOAc = 8 : 1).

##### (*R*)-2,2′-Dihydroxy-6,6′-dimethyl-1,1′-biphenyl (*R*)-2e

Colorless oil; [*α*]^22^_D_ + 36.8 (*c* 0.63, CHCl_3_); ^1^H-NMR (400 MHz, CDCl_3_) *δ* 7.37 (t, *J* = 8.0 Hz, 1H), 7.25 (d, *J* = 8.0 Hz, 2H), 7.17 (t, *J* = 8.0 Hz, 1H), 7.00 (d, *J* = 8.0 Hz, 1H), 6.83–6.86 (m, 2H), 4.82 (s, 1H), 2.04 (s, 3H), 1.94 (s, 3H), 1.91 (s, 3H); ^13^C-NMR (100 MHz, CDCl_3_) *δ* 170.5, 153.0, 149.6, 140.1, 137.6, 129.5, 129.1, 128.5, 128.3, 122.7, 122.1, 120.0, 113.9, 20.3, 19.6, 19.4; IR (CHCl_3_) *ν* 3532, 1744 cm^−1^; HRMS (MALDI) *m*/*z* calcd for C_16_H_16_O_3_Na [M + Na]^+^: 279.0997, found: 279.0992. Its optical purity (95% ee) was determined by HPLC analysis at 20 °C using a CHIRALPAK IC-3 column (hexanes/2-propanol = 99 : 1; flow rate: 1.0 mL min^−1^; retention times: 13.7 min (*R*), 16.9 min (*S*)).

##### (*S*)-2′-Hydroxy-6,6′-dimethyl-(1,1′-biphenyl)-2-yl acetate (*S*)-1e

White solid; mp 122–124 °C (lit.^29^ mp 159–160 °C); [*α*]^21^_D_ − 54.3 (*c* 0.50, CHCl_3_) (lit.^23^ [*α*]^20^_D_−60.5 (*c* 1.0, CHCl_3_) for (*S*)-1e with >99% ee); ^1^H-NMR (400 MHz, CDCl_3_) *δ* 7.21–7.27 (m, 2H), 6.86–6.92 (m, 4H), 4.71 (brs, 2H), 2.01 (s, 6H). The spectroscopic data are in good agreement with those reported.^29^ Its optical purity (97% ee) was determined by HPLC analysis at 20 °C using a CHIRALCEL OZ-3 column (hexanes/2-propanol = 95 : 5; flow rate: 1.0 mL min^−1^; retention times: 7.5 min (*R*), 10.4 min (*S*)).

### Kinetic resolution of non *C*_2_-symmetric biaryl diols (±)-1g–k (Table 5)

#### Representative procedure B (Table 5, entry 3)

To a stirred solution of (±)-1i (36.5 mg, 0.10 mmol) and vinyl acetate (93 μL, 1.0 mmol) in anhydrous PhCH_3_ (1.0 mL, 0.10 M) were added LIP301 (146 mg, 4 w/w) and Na_2_CO_3_ (32 mg, 0.30 mmol). After being stirred for 13 h at 35 °C, the reaction mixture was filtered through a Celite pad. The Celite pad was washed with EtOAc and the combined filtrate was evaporated *in vacuo*. The residue was purified by PTLC (PhCH_3_/EtOAc = 10 : 1) to give a 3 : 2 mixture of two regioisomeric esters (*R*)-2i (19.5 mg, 48% yield, 91% ee for each regioisomer) in addition to recovered (*S*)-1i (16.0 mg, 44% yield, >99% ee). The mixture of regioisomers (*R*)-2i was subjected to methanolysis as follows: to a stirred solution of (*R*)-2i (19.5 mg, 0.048 mmol, 91% ee) in MeOH (1.0 mL) was added K_2_CO_3_ (28 mg, 0.20 mmol). After being stirred for 15 min at room temperature, the reaction mixture was acidified with 1 N HCl and diluted with CH_2_Cl_2_. The mixture was transferred to a separating funnel and the organic layer was washed with brine. The organic layer was separated, dried over MgSO_4_, filtered, and concentrated *in vacuo* to give (*R*)-1i (17.4 mg, 48% yield from (±)-1i, 91% ee) as a pure pale-yellow solid.

##### (*R*)-3-Bromo-2′-hydroxy-(1,1′-binaphthalen)-2-yl acetate and (*R*)-3′-bromo-2′-hydroxy-(1,1′-binaphthalen)-2-yl acetate (*R*)-2i

Pale yellow solid; ^1^H-NMR (500 MHz, CDCl_3_) *δ* 8.35 (s, 0.4H), 8.22 (s, 0.6H), 8.07 (d, *J* = 9.0 Hz, 0.6H), 7.97 (d, *J* = 8.0 Hz, 0.6H), 7.92 (d, *J* = 9.0 Hz, 0.4H), 7.89 (d, *J* = 9.0 Hz, 0.4H), 7.86 (d, *J* = 8.0 Hz, 0.4H), 7.78 (d, *J* = 8.0 Hz, 0.6H), 7.51 (m, 1H), 7.43 (d, *J* = 9.0 Hz, 0.6H), 7.32–7.37 (m, 2.4H), 7.25–7.28 (m, 1H), 7.22 (d, *J* = 9.0 Hz, 0.6H) 7.20 (d, *J* = 9.0 Hz, 0.4H), 7.04 (d, *J* = 9.0 Hz, 0.6H), 7.01 (d, *J* = 9.0, 0.4H), 5.30–5.64 (brs, 1H), 1.97 (s, 1.2H), 1.88 (s, 1.8H). Its optical purity (91% ee) was determined by HPLC analysis at 20 °C using a CHIRALCEL IC-3 column (hexanes/2-propanol) = 95 : 5; flow rate: 1.0 mL min^−1^; retention times for (*R*)-regioisomers of 2i: 6.7 min and 8.9 min; retention times for (*S*)-regioisomers of 2i: 8.0 min and 10.0 min.

##### (*R*)-3-Bromo-1,1′-bi-2-naphthol (*R*)-1i

White solid; mp 82–89 °C (lit.^9^ mp 130–132 °C); [*α*]^23^_D_ + 56.5 (*c* 0.28, CHCl_3_) (lit.^9^ [*α*]^20^_D_ + 38.3 (*c* 0.23, CHCl_3_) for (*R*)-1i with 86% ee); ^1^H-NMR (400 MHz, CDCl_3_) *δ* 8.28 (s, 1H), 7.98 (d, *J* = 9.0 Hz, 1H), 7.90 (d, *J* = 8.0 Hz, 1H), 7.83 (d, *J* = 8.0 Hz, 1H), 7.28–7.42 (m, 5H), 7.14 (d, *J* = 7.0 Hz, 1H), 7.10 (d, *J* = 9.0 Hz, 1H), 5.58 (s, 1H), 4.95 (s, 1H). The spectroscopic data are in good agreement with those reported.^9^ Its optical purity (91% ee) was determined by HPLC analysis at 20 °C using a CHIRALPAK IC-3 column (hexanes/2-propanol = 95 : 5; flow rate: 1.0 mL min^−1^; retention times: 10.1 min (*R*), 27.0 min (*S*)).

##### (*S*)-3-Bromo-1,1′-bi-2-naphthol (*S*)-1i

White solid; mp 64–68 °C; [α]^24^_D_ − 64.1 (*c* 0.39, CHCl_3_). Its spectroscopic data are in good agreement with those described above for (*R*)-1i. Its optical purity (>99% ee) was determined by HPLC analysis at 20 °C using a CHIRALPAK IC-3 column (hexanes/2-propanol = 95 : 5; flow rate: 1.0 mL min^−1^; retention times: 11.4 min (*R*), 26.5 min (*S*)).

#### Table 5, entry 1

Following the above-mentioned procedure B, (±)-1g (36 mg, 0.10 mmol) was treated with vinyl acetate (93 μL, 1.0 mmol) and LIP301 (108 mg, 3 w/w) and Na_2_CO_3_ (32 mg, 0.30 mmol) to give (*R*)-2g (19.2 mg, 48% yield) and (*S*)-1g (18.0 mg, 51% yield, 93% ee) after purification of the crude reaction mixture by PTLC (PhCH_3_/EtOAc = 10 : 1). Methanolysis of (*R*)-2g (19.2 mg, 0.048 mmol), as described above, afforded (*R*)-1g (17.0 mg, 48% yield from (±)-1g, 95% ee).

##### Ethyl (*R*)-2-acetoxy-2′-hydroxy-(1,1′-binaphthalene)-6-carboxylate and ethyl (*R*)-2′-acetoxy-2-hydroxy-(1,1′-binaphthalene)-6-carboxylate (*R*)-2g

Pale yellow solid; ^1^H-NMR (500 MHz, CDCl_3_) *δ* 8.58 (d, *J* = 2.0 Hz, 0.3H), 8.48 (d, *J* = 1.0 Hz, 0.7H), 8.04 (d, *J* = 9.0 Hz, 0.3H), 7.95 (d, *J* = 9.0 Hz, 0.7H), 7.88 (d, *J* = 9.0 Hz, 0.7H), 7.84 (d, *J* = 9.0 Hz, 0.7H), 7.76–7.79 (m, 0.6H), 7.73 (d, *J* = 8.0 Hz, 0.3H), 7.70 (dd, *J* = 9.0, 2.0 Hz, 0.7H), 7.37–7.40 (m, 0.7H), 7.33 (d, *J* = 9.0 Hz, 0.3H), 7.19–7.28 (m, 1.7H), 7.16 (d, *J* = 9.0 Hz, 0.3H), 7.07–7.13 (m, 1H), 6.93 (d, *J* = 9.0 Hz, 0.7H), 6.84 (d, *J* = 8.0 Hz, 0.3H), 5.07–5.26 (m, 1H), 4.24–4.31 (m, 2H), 1.74 (s, 0.9H), 1.72 (s, 2.1H), 1.27 (m, 3H).

##### Ethyl (*R*)-2,2′-dihydroxy-(1,1′-binaphthalene)-6-carboxylate (*R*)-1g

White solid; mp 83–85 °C (lit.^9^ mp 98–100 °C); [*α*]^22^_D_ − 72.8 (*c* 0.39, CHCl_3_) (lit.^9^ [*α*]^21^_D_ − 54.6 (*c* 0.7, CHCl_3_) for (*R*)-1g with 89% ee); ^1^H-NMR (500 MHz, CDCl_3_) *δ* 8.60 (s, 1H), 8.06 (d, *J* = 9.0 Hz, 1H), 7.98 (d, *J* = 9.0 Hz, 1H), 7.90 (d, *J* = 9.0 Hz, 1H), 7.84 (dd, *J* = 9.0, 2.0 Hz, 1H), 7.43 (d, *J* = 9.0 Hz, 1H), 7.36–7.39 (m, 2H), 7.31 (td, *J* = 7.0, 2.0 Hz, 1H), 7.16 (d, *J* = 9.0 Hz, 1H), 7.09 (d, *J* = 9.0 Hz, 1H), 5.31 (s, 2H), 5.21 (s, 1H), 4.38 (q, *J* = 7.0 Hz, 2H), 1.41 (t, *J* = 7.0 Hz, 3H). The spectroscopic data are in good agreement with those reported.^9^ Its optical purity (95% ee) was determined by HPLC analysis at 20 °C using a CHIRALCEL OD-3 column (hexanes/2-propanol = 85 : 15; flow rate: 1.0 mL min^−1^; retention times: 19.5 min (*R*), 14.0 min (*S*)).

##### Ethyl (*S*)-2,2′-dihydroxy-(1,1′-binaphthalene)-6-carboxylate (*S*)-1g

White solid; mp 93–95 °C; [*α*]^21^_D_ + 67.5 (*c* 0.82, CHCl_3_). Its spectroscopic data are in good agreement with those described above for (*R*)-1g. Its optical purity (93% ee) was determined by HPLC analysis at 20 °C using a CHIRALPAK IC-3 column (hexanes/2-propanol = 85 : 15; flow rate: 1.0 mL min^−1^; retention times: 18.8 min (*R*), 12.9 min (*S*)).

#### Table 5, entry 2

Following the above-mentioned procedure B, (±)-1h (49.8 mg, 0.10 mmol) was treated with vinyl acetate (93 μL, 1.0 mmol) and LIP301 (149 mg, 3 w/w) and Na_2_CO_3_ (16.0 mg, 0.15 mmol) to give to (*R*)-2h (25.4 mg, 47% yield) and (*S*)-1h (20.1 mg, 40% yield, >99% ee), after purification of the crude reaction mixture by flash column chromatography (PhCH_3_/EtOAc = 20 : 1). Methanolysis of (*R*)-2h (25.4 mg, 0.047 mmol), as described above, afforded (*R*)-1h (23.0 mg, 47% yield from (±)-1h, 95% ee).

##### (*R*)-6-(3,5-Bis(trifluoromethyl)phenyl)-2′-hydroxy-(1,1′-binaphthalen)-2-yl acetate and (*R*)-6′-(3,5-bis(trifluoromethyl)phenyl)-2′-hydroxy-(1,1′-binaphthalen)-2-yl acetate (*R*)-2h

Pale yellow solid. ^1^H-NMR (400 MHz, CDCl_3_) *δ* 7.85–8.22 (m, 7H), 7.35–7.58 (m, 5H), 7.25–7.30 (m, 1H), 7.18 (d, *J* = 9.0 Hz, 0.7H), 7.04 (d, *J* = 8.0 Hz, 0.3H), 5.32 (s, 0.7H), 5.23 (s, 0.3H), 1.91 (s, 2H), 1.90 (s, 1H).

##### (*R*)-6-(3,5-Bis(trifluoromethyl)phenyl)-1,1′-bi-2-naphthol (*R*)-1h

White solid; mp 94–95 °C; [*α*]^22^_D_ − 71.2 (*c* 0.86, CHCl_3_) (lit.^4*e*^ [*α*]^22^_D_ − 39.8 (*c* 5.53, CHCl_3_) for (*R*)-1h with 72% ee); ^1^H-NMR (400 MHz, CDCl_3_) *δ* 8.06–8.12 (m, 4H), 8.01 (d, *J* = 8.5 Hz, 1H), 7.92 (d, *J* = 8.0 Hz, 1H), 7.87 (s, 1H), 7.53 (dd, *J* = 8.5, 1.5 Hz, 1H), 7.46 (d, *J* = 9.0 Hz, 1H), 7.40 (d, *J* = 9.0 Hz, 2H), 7.34 (t, *J* = 7.5 Hz, 1H), 7.28 (d, *J* = 8.5 Hz, 1H), 7.16 (d, *J* = 8.5 Hz, 1H), 5.18 (s, 1H), 5.06 (s, 1H). The spectroscopic data are in good agreement with those reported.^4*e*^ Its optical purity (95% ee) was determined by HPLC analysis at 20 °C using a CHIRALPAK IC-3 column (hexanes/2-propanol = 95 : 5; flow rate: 1.0 mL min^−1^; retention times: 9.4 min (*R*), 11.9 min (*S*)).

##### (*S*)-6-(3,5-Bis(trifluoromethyl)phenyl)-1,1′-bi-2-naphthol (*S*)-1h

White solid; mp 91–93 °C; [*α*]^22^_D_ + 69.1 (*c* 0.97, CHCl_3_). Its spectroscopic data are in good agreement with those described above for (*R*)-1h. Its optical purity (>99% ee) was determined by HPLC analysis at 20 °C using a CHIRALPAK IC-3 column (hexanes/2-propanol = 95 : 5; flow rate: 1.0 mL min^−1^; retention times: 9.7 min (*R*), 11.6 min (*S*)).

#### Table 5, entry 5

Following the above-mentioned procedure B, (±)-1j (30.0 mg, 0.10 mmol) was treated with vinyl acetate (93 μL, 1.0 mmol) and LIP301 (90 mg, 3 w/w) and Na_2_CO_3_ (16.0 mg, 0.15 mmol) to give to (*R*)-2j (15.4 mg, 45% yield) and (*S*)-1j (12.4 mg, 41% yield, 99% ee) after purification of the crude reaction mixture by gel permeation chromatography using chloroform as an eluent. Methanolysis of (*R*)-2j (15.4 mg, 0.045 mmol) as described above, afforded (*R*)-1j (13.4 mg, 45% yield from (±)-1j, 92% ee).

##### (*R*)-2-Hydroxy-3′-methyl-(1,1′-binaphthalen)-2-yl acetate and (*R*)-2′-hydroxy-3′-methyl-(1,1′-binaphthalen)-2-yl acetate (*R*)-2j

White solid; ^1^H-NMR (500 MHz, CDCl_3_) *δ* 8.07 (d, *J* = 9.0 Hz, 0.7H), 7.97 (d, *J* = 8.0 Hz, 0.7H), 7.85–7.92 (m, 1H), 7.78 (d, *J* = 8.0 Hz, 0.7H), 7.74 (s, 0.7H), 7.53–7.16 (m, 6.2H), 7.03 (d, *J* = 8.6 Hz, 0.3H), 6.97 (d, *J* = 9.0 Hz, 0.7H), 5.21 (s, 1H), 2.50 (s, 2.1H), 2.44 (s, 0.9H), 1.87 (s, 2.1H), 1.86 (s, 0.9H).

##### (*R*)-3-Methyl-1,1′-bi-2-naphthol (*R*)-1j

White solid; mp 94–96 °C; [*α*]^22^_D_ + 17.4 (*c* 0.75, CDCl_3_) (lit.^30^ [*α*]^20^_D_ + 22.1 (*c* 1.5, CDCl_3_) for (*R*)-1j with 95% ee); ^1^H-NMR (500 MHz, CDCl_3_) *δ* 7.98 (d, *J* = 9.0 Hz, 1H), 7.90 (d, *J* = 8.0 Hz, 1H), 7.81–7.83 (m, 2H), 7.29–7.40 (m, 4H), 7.23–7.26 (m, 1H), 7.15 (d, *J* = 9.0 Hz, 1H), 7.08 (d, *J* = 8.0 Hz, 1H), 5.12 (brs, 1H), 5.07 (brs, 1H), 2.52 (s, 3H). The spectroscopic data are in good agreement with those reported.^30^ Its optical purity (92% ee) was determined by HPLC analysis at 20 °C using a CHIRALPAK IC-3 column (hexanes/2-propanol = 95 : 5; flow rate: 1.0 mL min^−1^; retention times: 6.0 min (*R*), 9.3 min (*S*)).

##### (*S*)-3-Methyl-1,1′-bi-2-naphthol (*S*)-1j

White solid; mp 93–99 °C; [*α*]^22^_D_ − 19.4 (*c* 0.75, CDCl_3_). Its spectroscopic data are in good agreement with those described above for (*R*)-1j. Its optical purity (99% ee) was determined by HPLC analysis at 20 °C using a CHIRALPAK IC-3 column (hexanes/2-propanol = 95 : 5; flow rate: 1.0 mL min^−1^; retention times: 6.1 min (*R*), 9.4 min (*S*)).

#### Table 5, entry 6

Following the above-mentioned procedure B, (±)-1k (41.4 mg, 0.10 mmol) was treated with vinyl acetate (93 μL, 1.0 mmol) and LIP301 (124 mg, 3 w/w) and Na_2_CO_3_ (16 mg, 0.15 mmol) to give to (*R*)-2k (19.6 mg, 43% yield) and (*S*)-1k (18.2 mg, 44% yield, 99% ee), after purification of the crude reaction mixture by PTLC (PhCH_3_/EtOAc = 10 : 1). Methanolysis of (*R*)-2k (19.6 mg, 0.043 mmol), as described above, afforded (*R*)-1k (17.9 mg, 43% yield from (±)-1k, 96% ee).

##### (*R*)-6-Bromo-1-(2,5-dichloro-6-hydroxy-3-methoxyphenyl)naphthalen-2-yl acetate and (*R*)-2-(6-bromo-2-hydroxynaphthalen-1-yl)-3,6-dichloro-4-methoxyphenyl acetate (*R*)-2k

Pale yellow solid; ^1^H-NMR (500 MHz, CDCl_3_) *δ* 8.08 (d, *J* = 2.0 Hz, 0.2H), 8.96 (d, *J* = 2.0 Hz, 0.8H), 7.89 (d, *J* = 9.0 Hz, 0.2H), 7.74 (d, *J* = 9.0 Hz, 0.8H), 7.49 (dd, *J* = 9.0, 2.0 Hz, 0.2H), 7.38–7.43 (m, 1H), 7.25–7.27 (m, 0.8H), 7.21 (s, 0.8H), 7.19 (d, *J* = 8.0 Hz, 0.2H), 7.10 (s, 0.2H), 7.01 (d, *J* = 9.0 Hz, 0.8H), 5.23–5.30 (brs, 1H), 3.99 (s, 2.4H), 3.93 (s, 0.6H), 2.11 (s, 0.6H), 1.86 (s, 2.4H).

##### (*R*)-6-Bromo-1-(2,5-dichloro-6-hydroxy-3-methoxyphenyl)naphthalen-2-ol (*R*)-1k

White solid; mp 72–74 °C; [*α*]^22^_D_ + 20.2 (*c* 0.83, CHCl_3_); ^1^H-NMR (500 MHz, CDCl_3_) *δ* 8.00 (d, *J* = 2.0 Hz, 1H), 7.80 (d, *J* = 9.0 Hz, 1H), 7.44 (dd, *J* = 9.0, 2.0 Hz, 1H), 7.30 (d, *J* = 9.0 Hz, 1H), 7.14 (s, 1H), 7.08 (d, *J* = 9.0 Hz, 1H), 5.12 (s, 1H), 5.03 (s, 1H), 3.95 (s, 3H); ^13^C-NMR (100 MHz, CDCl_3_) *δ* 151.4, 149.9, 144.6, 130.9, 130.5, 130.3, 130.24, 130.20, 125.5, 123.3, 121.6, 119.0, 118.9, 117.7, 113.7, 113.0, 56.9; IR (neat) *ν* 3517 cm^−1^; HRMS (MALDI) *m*/*z* calcd for C_17_H_11_^79^Br^35^Cl_2_O_3_ [M]^+^: 411.9263, found: 411.9263. Its optical purity (96% ee) was determined by HPLC analysis at 20 °C using a CHIRALPAK IE column (hexanes/2-propanol = 95 : 5; flow rate: 1.0 mL min^−1^; retention times: 22.3 min (*R*), 26.8 min (*S*)).

##### (*S*)-6-Bromo-1-(2,5-dichloro-6-hydroxy-3-methoxyphenyl)naphthalen-2-ol (*S*)-1k

White solid; mp 65–68 °C; [*α*]^22^_D_ − 22.7 (*c* 0.81, CHCl_3_). Its spectroscopic data are in good agreement with those described above for (*R*)-1k. Its optical purity (99% ee) was determined by HPLC analysis at 20 °C using a CHIRALPAK IE column (hexanes/2-propanol = 95 : 5; flow rate: 1.0 mL min^−1^; retention times: 22.5 min (*R*), 24.7 min (*S*)).

### Kinetic resolution of biaryl methanol derivatives (±)-4a–b (Table 6)

#### Representative procedure C: Table 6, entry 3

To a stirred solution of (±)-4a (28 mg, 0.10 mmol) and vinyl acetate (93 μL, 1.0 mmol) in anhydrous PhCH_3_ (1.0 mL, 0.10 M) was added LIP301 (84 mg, 3 w/w). After being stirred for 48 h at 25 °C, the reaction mixture was filtered through a Celite pad. The Celite pad was washed with EtOAc and the combined filtrate was evaporated *in vacuo*. The residue was purified by flash column chromatography (hexanes/EtOAc = 3 : 1) to give ester (*R*)-5a (18.1 mg, 55% yield, 88% ee) and recovered (*S*)-4a (12.0 mg, 43% yield, 97% ee).

##### (*R*)-(1-(2-Hydroxy-4,6-dimethylphenyl)naphthalen-2-yl)methyl acetate (*R*)-5a

Colorless oil; [*α*]^23^_D_ − 22.9 (*c* 0.79, CHCl_3_); ^1^H-NMR (500 MHz, CDCl_3_) *δ* 7.95 (d, *J* = 8.5 Hz, 1H), 7.90 (d, *J* = 8.0 Hz, 1H), 7.62 (d, *J* = 8.5 Hz, 1H), 7.50–7.53 (m, 1H), 7.39–7.42 (m, 2H), 6.77 (s, 1H), 6.75 (s, 1H), 4.99 (d, *J* = 12.5 Hz, 1H), 4.96 (d, *J* = 12.5 Hz, 1H), 4.54 (s, 1H), 2.38 (s, 3H), 2.05 (s, 3H), 1.79 (s, 3H); ^13^C-NMR (125 MHz, CDCl_3_) *δ* 170.9, 153.1, 139.4, 137.9, 133.6, 133.3, 132.5, 132.1, 129.0, 128.2, 127.1, 126.6, 126.1, 125.5, 123.3, 120.3, 113.9, 64.8, 21.3, 20.8, 19.7; IR (CHCl_3_) *ν* 3548, 1737 cm^−1^; HRMS (MALDI) *m*/*z* calcd for C_21_H_20_O_3_Na [M + Na]^+^: 343.1310, found: 343.1305. Its optical purity (88% ee) was determined by HPLC analysis at 20 °C using a CHIRALPAK AD-3 column (hexanes/2-propanol = 95 : 5; flow rate: 1.0 mL min^−1^; retention times: 15.5 min (*R*), 17.5 min (*S*)).

##### (*S*)-2-(2-(Hydroxymethyl)naphthalen-1-yl)-3,5-dimethylphenol (*S*)-4a

White solid; mp 140–141 °C; [*α*]^23^_D_ + 32.6 (*c* 0.75, CHCl_3_) (lit.^14^ [*α*]^20^_D_ + 21.9 (*c* 0.8, CHCl_3_) for (*S*)-4a with 79% ee); ^1^H-NMR (500 MHz, CDCl_3_) *δ* 7.96 (d, *J* = 8.5 Hz, 1H), 7.90 (d, *J* = 8.0 Hz, 1H), 7.72 (d, *J* = 8.0 Hz, 1H), 7.48–7.51 (m, 1H), 7.38–7.39 (m, 2H), 6.79 (s, 1H), 6.75 (s, 1H), 4.53 (s, 2H), 2.38 (s, 3H), 1.80 (s, 3H). The spectroscopic data are in good agreement with those reported.^14^ Its optical purity (97% ee) was determined by HPLC analysis at 20 °C using a CHIRALPAK AD-3 column (hexanes/2-propanol = 85 : 15; flow rate: 1.0 mL min^−1^; retention times: 8.8 min (*R*), 11.2 min (*S*)).

#### Table 6, entry 4

Following procedure C described above, (±)-4b (27.8 mg, 0.10 mmol) was converted to (*R*)-5b (15.7 mg, 49% yield, 93% ee) and (*S*)-4b (14.2 mg, 51% yield, 98% ee), after purification of the crude reaction mixture by flash column chromatography (hexanes : EtOAc = 8 : 1).

##### (*R*)-(1-(2-Hydroxy-3,6-dimethylphenyl)naphthalen-2-yl)methyl acetate (*R*)-5b

Colorless oil; [*α*]^21^_D_ − 13.7 (*c* 0.51, CHCl_3_); ^1^H-NMR (400 MHz, CDCl_3_) *δ* 7.96 (d, *J* = 8.5 Hz, 1H), 7.91 (d, *J* = 8.0 Hz, 1H), 7.63 (d, *J* = 8.0 Hz, 1H), 7.50–7.54 (m, 1H), 7.35–7.42 (m, 2H), 7.14 (d, *J* = 7.5 Hz, 1H), 6.84 (d, *J* = 7.5 Hz, 1H), 4.99 (d, *J* = 12.5 Hz, 1H), 4.94 (d, *J* = 12.5 Hz, 1H), 4.58 (s, 1H), 2.28 (s, 3H), 2.04 (s, 3H), 1.78 (s, 3H); ^13^C-NMR (100 MHz, CDCl_3_) *δ* 170.8, 151.3, 135.3, 133.6, 133.2, 132.4, 132.3, 130.6, 129.1, 128.2, 127.1, 126.7, 126.2, 125.5, 122.7, 121.9, 121.7, 64.8, 20.8, 19.6, 16.0; IR (CHCl_3_) *ν* 3553, 1737 cm^−1^; HRMS (MALDI) *m*/*z* calcd for C_21_H_20_O_3_Na [M + Na]^+^: 343.1310, found: 343.1305. Its optical purity (93% ee) was determined by HPLC analysis at 20 °C using a CHIRALPAK AD-3 column (hexanes/2-propanol = 95 : 5; flow rate: 1.0 mL min^−1^; retention times: 8.5 min (*S*), 10.6 min (*R*)).

##### (*S*)-2-(2-(Hydroxymethyl)naphthalen-1-yl)-3,6-dimethylphenol (*S*)-4b

Colorless oil; [*α*]^21^_D_ + 21.7 (*c* 0.6, CHCl_3_) (lit.^14^ [*α*]^20^_D_ + 27.2 (*c* 0.4, CHCl_3_) for (*S*)-4b with 87% ee); ^1^H-NMR (500 MHz, CDCl_3_) *δ* 7.98 (d, *J* = 8.5 Hz, 1H), 7.91 (d, *J* = 8.0 Hz, 1H), 7.75 (d, *J* = 8.5 Hz, 1H), 7.49–7.52 (m, 1H), 7.34–7.41 (m, 2H), 7.16 (d, *J* = 7.5 Hz, 1H), 6.86 (d, *J* = 7.5 Hz, 1H), 4.52 (s, 2H), 2.29 (s, 3H), 1.79 (s, 3H). The spectroscopic data are in good agreement with those reported.^14^ Its optical purity (98% ee) was determined by HPLC analysis at 20 °C using a CHIRALPAK AD-3 column (hexanes/2-propanol = 90 : 10; flow rate: 1.0 mL min^−1^; retention times: 12.9 min (*R*), 29.8 min (*S*)).

## Conflicts of interest

There are no conflicts to declare.

## Supplementary Material

RA-009-C8RA09070J-s001
