# Supplementary material for: Base-promoted lipase-catalyzed kinetic resolution of atropisomeric 1,1′-biaryl-2,2′-diols
Source: RSC Adv. 2019 Jan 9;9(3):1165–75. doi: 10.1039/c8ra09070j (PMC9059663; doi:10.1039/c8ra09070j)
Supplement: RA-009-C8RA09070J-s001 [file RA-009-C8RA09070J-s001.pdf]

Electronic Supplementary Material (ESI) for RSC Advances.
This journal is © The Royal Society of Chemistry 2018

Supporting Information for:

Base-promoted lipase-catalyzed kinetic resolution of atropisomeric 1,1'-biaryl-2,2'-diols

Gamal A. I. Moustafa,[a,b] Kengo Kasama,[a] Koichi Higashio,[a] and Shuji Akai*[a]

[a]Graduate School of Pharmaceutical Sciences, Osaka University, 1-6 Yamadaoka, Suita, Osaka 565-0871, Japan.

[b]Department of Medicinal Chemistry, Faculty of Pharmacy, Minia University, Minia 61519, Egypt.

E-mail: akai@phs.osaka-u.ac.jp.

Contents

NMR spectra and HPLC charts of racemic and enantioenriched compounds………………S2-S59

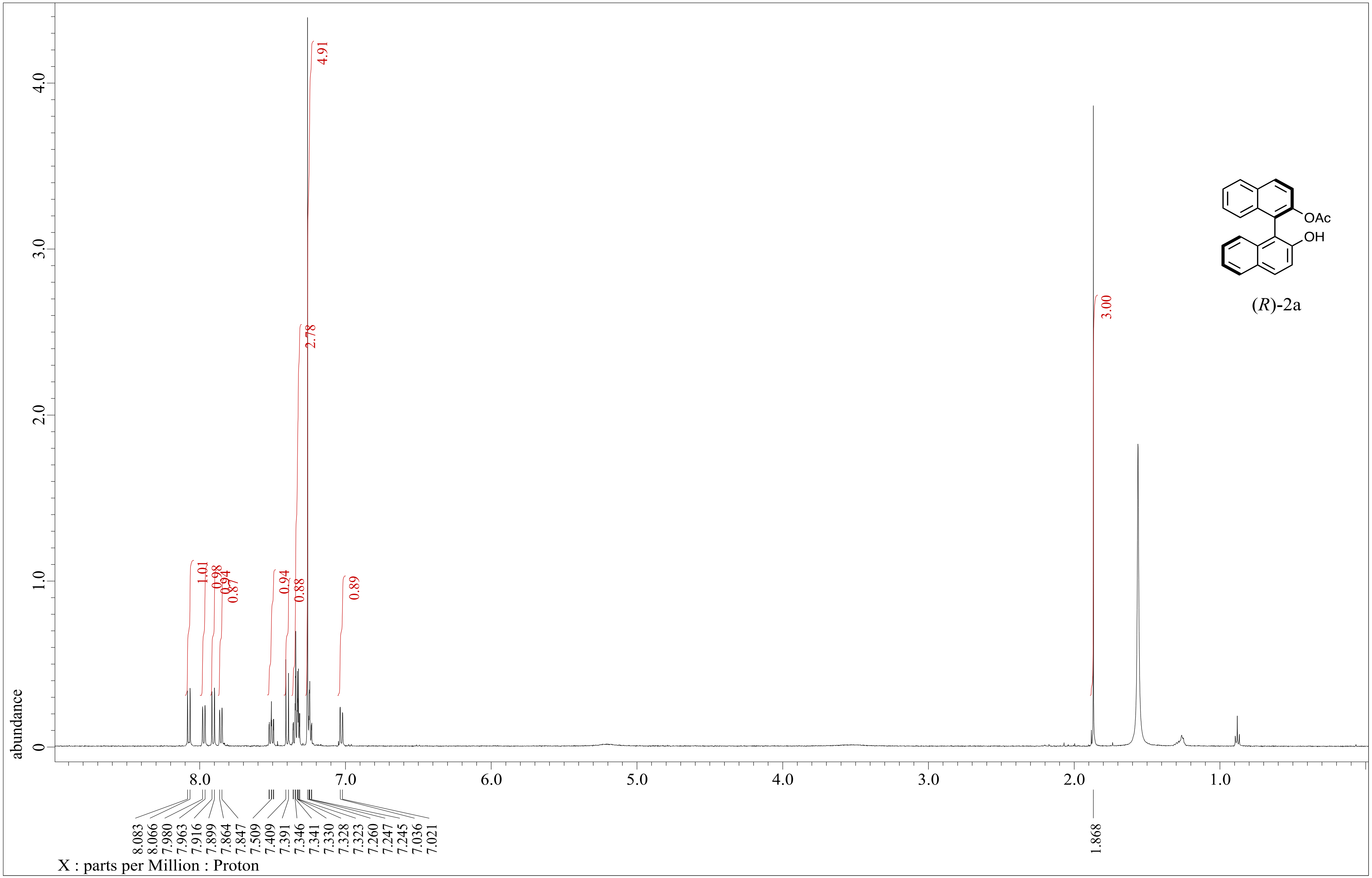

^{1}H NMR spectrum (500 MHz, $CDCl_3$) of (*R*)-**2a**

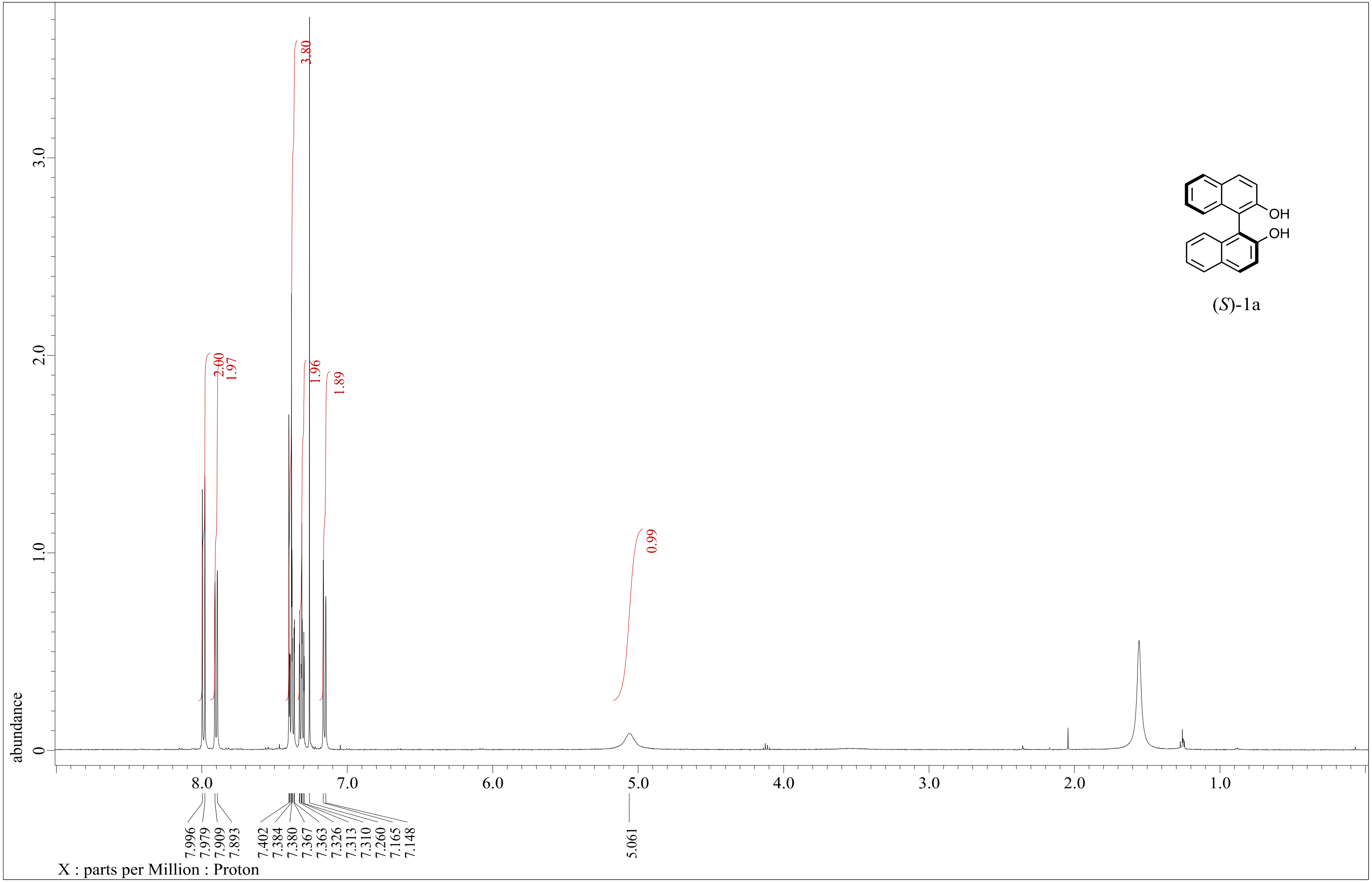

^{1}H NMR spectrum (500 MHz, $CDCl_3$) of (*S*)-**1a**

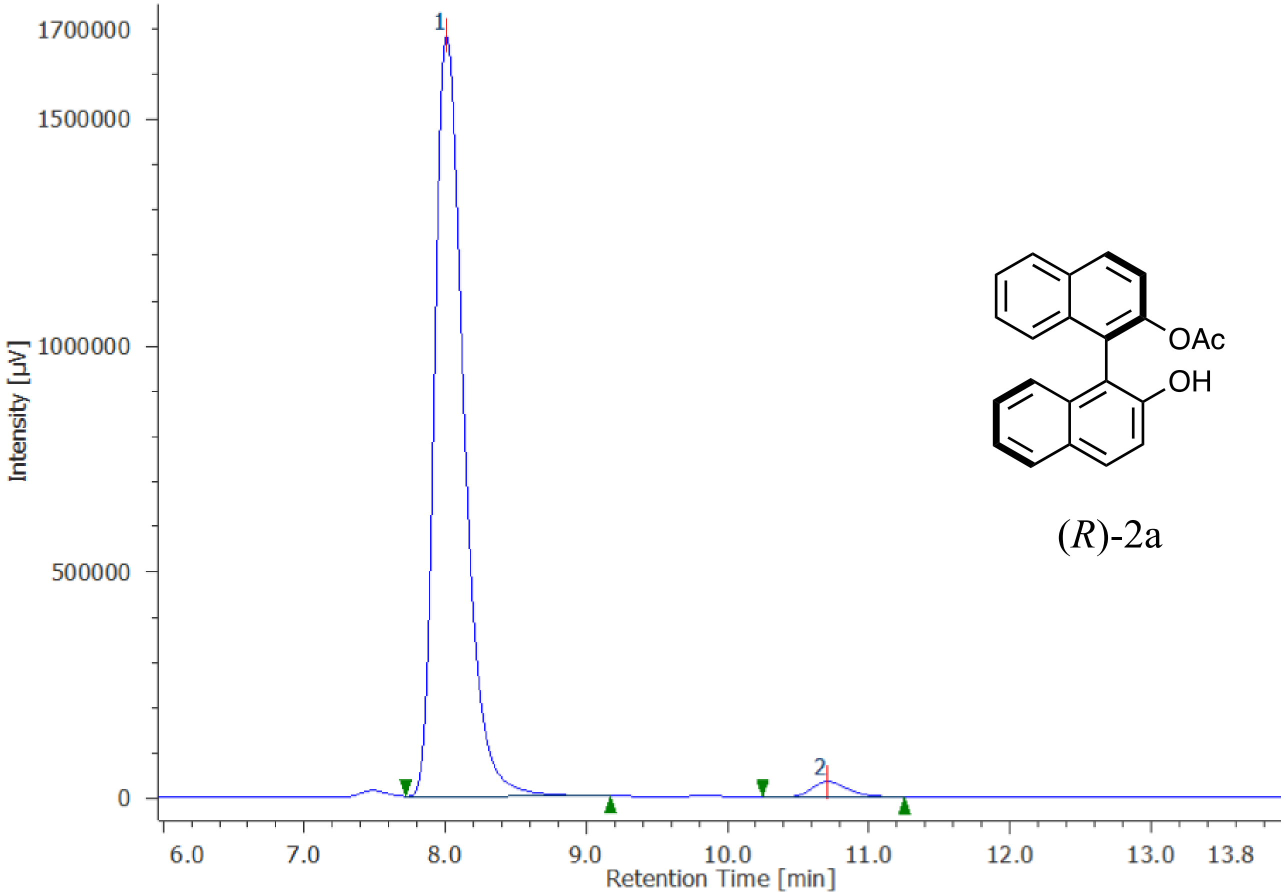

#	ピーク名	CH	tR [min]	面積 [µV·sec]	高さ [µV]	面積%	高さ%	定量値	NTP	分離度	シンメトリー係数	警告
1	Unknown	9	8.010	24409638	1682764	97.585	98.001	N/A	7577	6.583	1.402	
2	Unknown	9	10.710	604182	34328	2.415	1.999	N/A	8887	N/A	1.251	

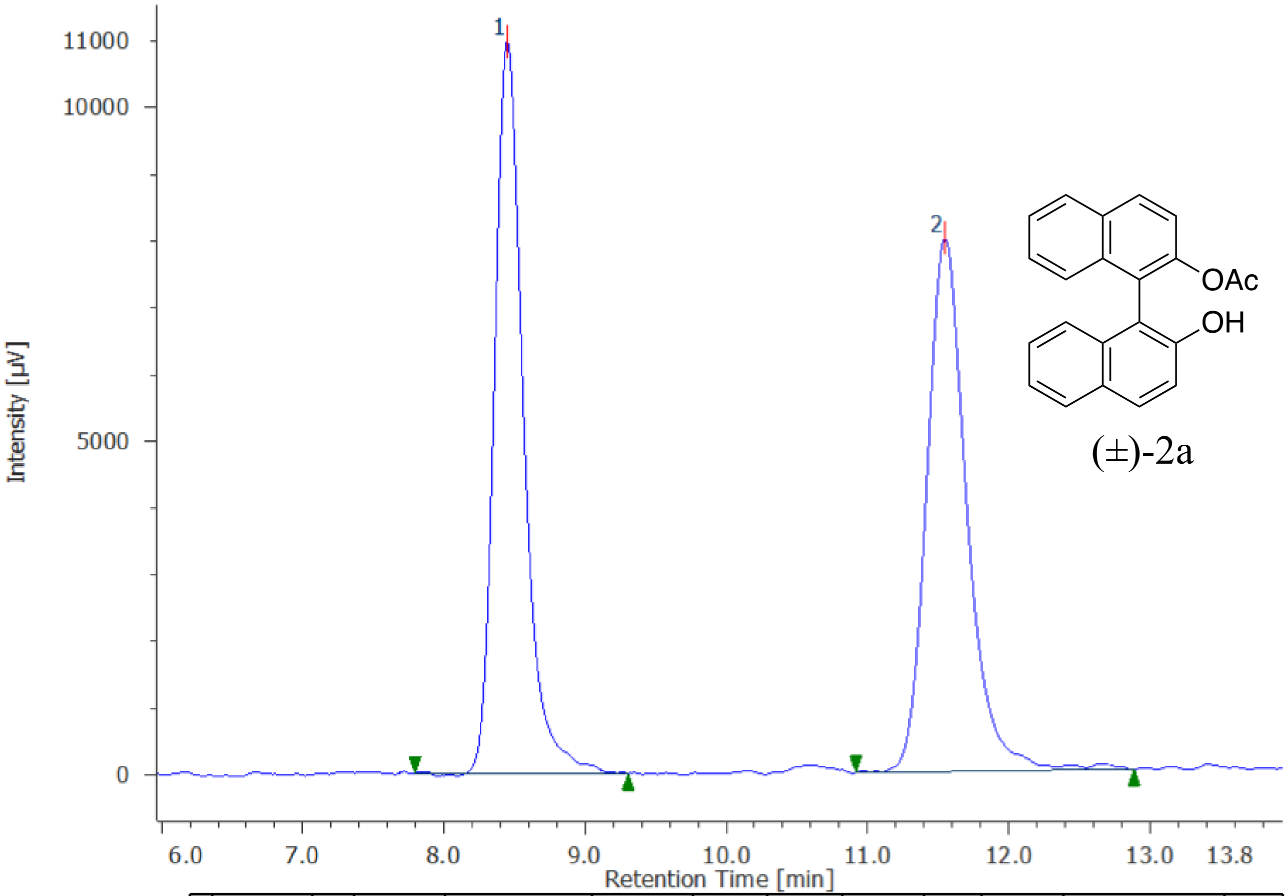

#	ピーク名	CH	tR [min]	面積 [µV·sec]	高さ [µV]	面積%	高さ%	定量値	NTP	分離度	シンメトリー係数	警告
1	Unknown	9	8.447	157049	10972	49.694	57.866	N/A	8929	7.271	1.320	
2	Unknown	9	11.543	158983	7989	50.306	42.134	N/A	8650	N/A	1.258	

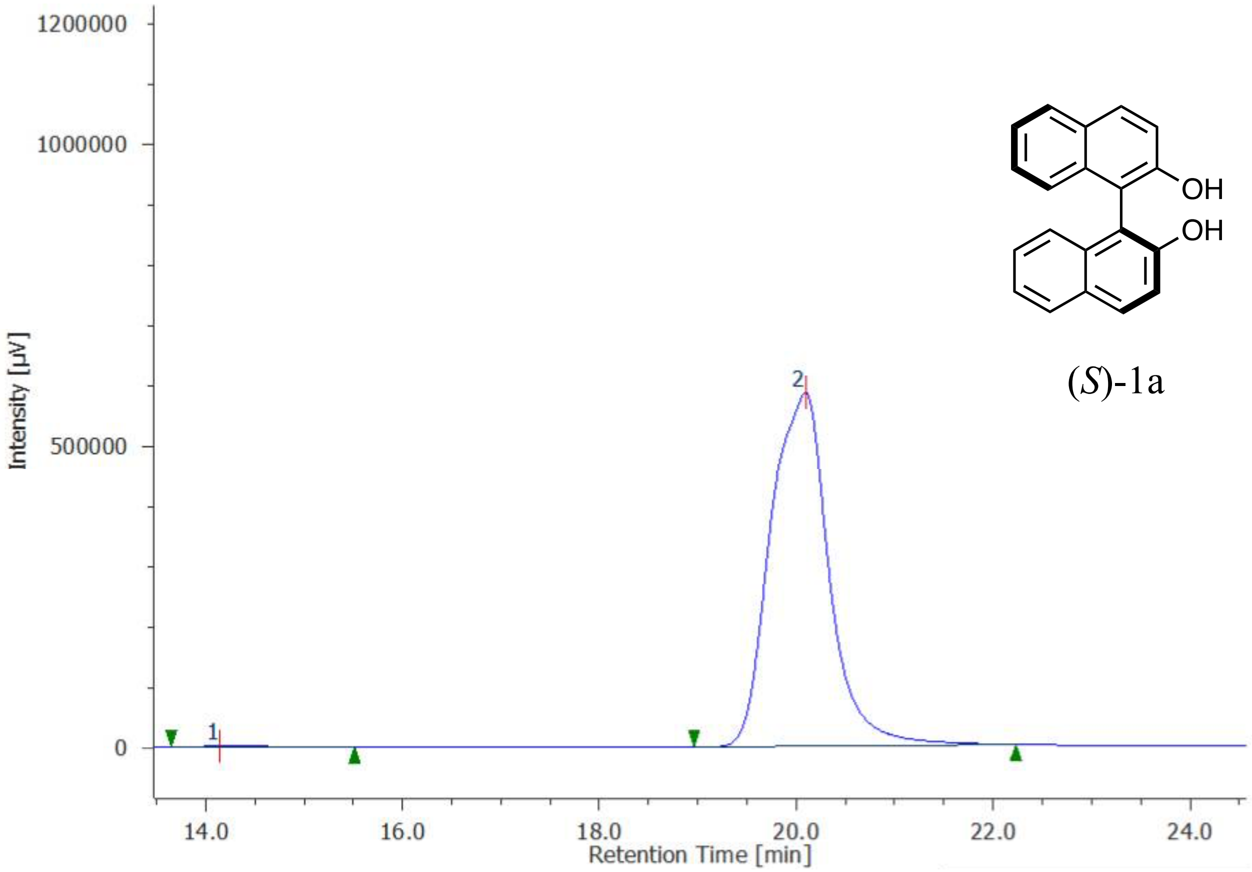

#	ピーク名	CH	tR [min]	面積 [µV·sec]	高さ [µV]	面積%	高さ%	定量値	NTP	分離度	シンメトリー係数	警告
1	Unknown	9	14.147	44389	998	0.184	0.170	N/A	2006	5.074	2.060	
2	Unknown	9	20.093	24142267	585458	99.816	99.830	N/A	5469	N/A	1.048	

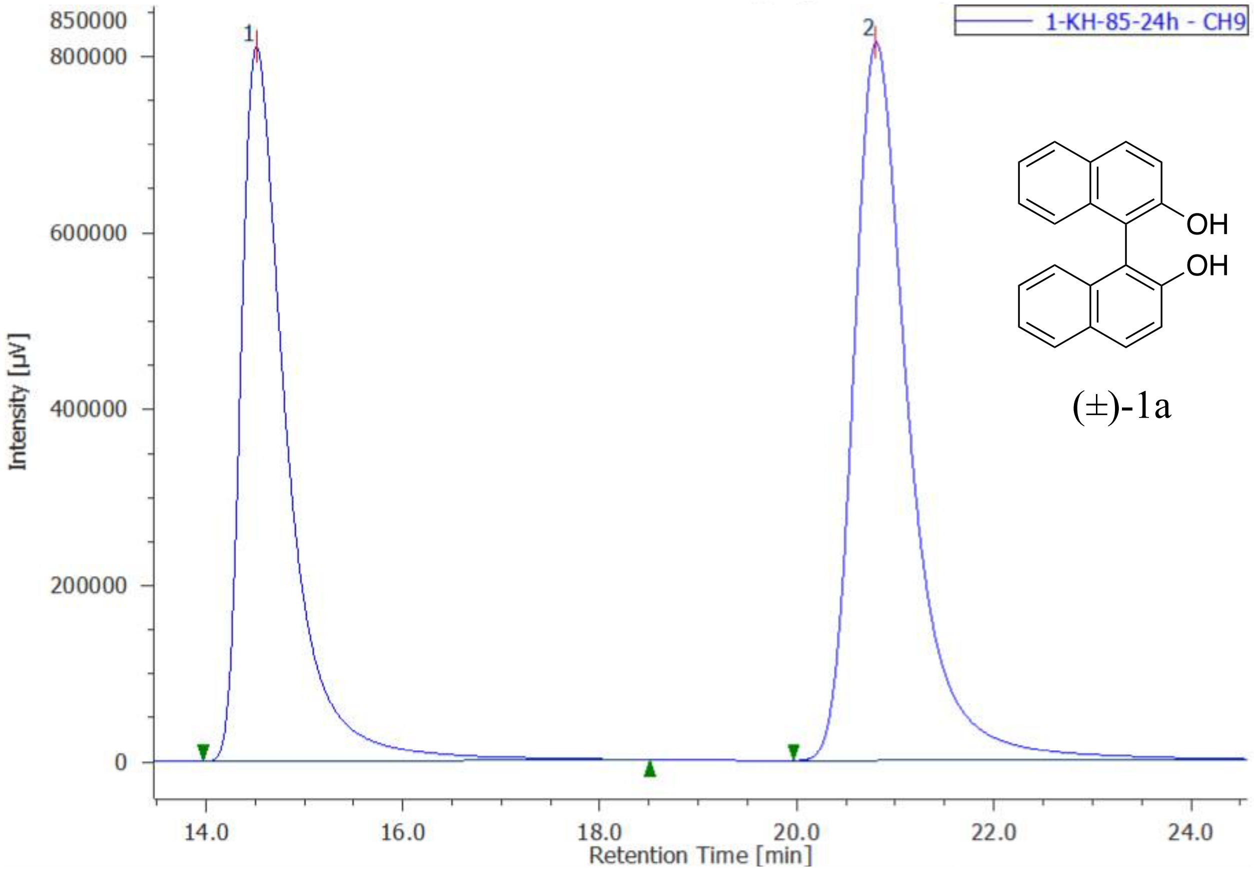

#	ピーク名	CH	tR [min]	面積 [µV·sec]	高さ [µV]	面積%	高さ%	定量値	NTP	分離度	シンメトリー係数	警告
1	Unknown	9	14.513	26492163	810738	44.923	49.888	N/A	5630	7.282	1.951	
2	Unknown	9	20.803	32480328	814371	55.077	50.112	N/A	7538	N/A	1.512	

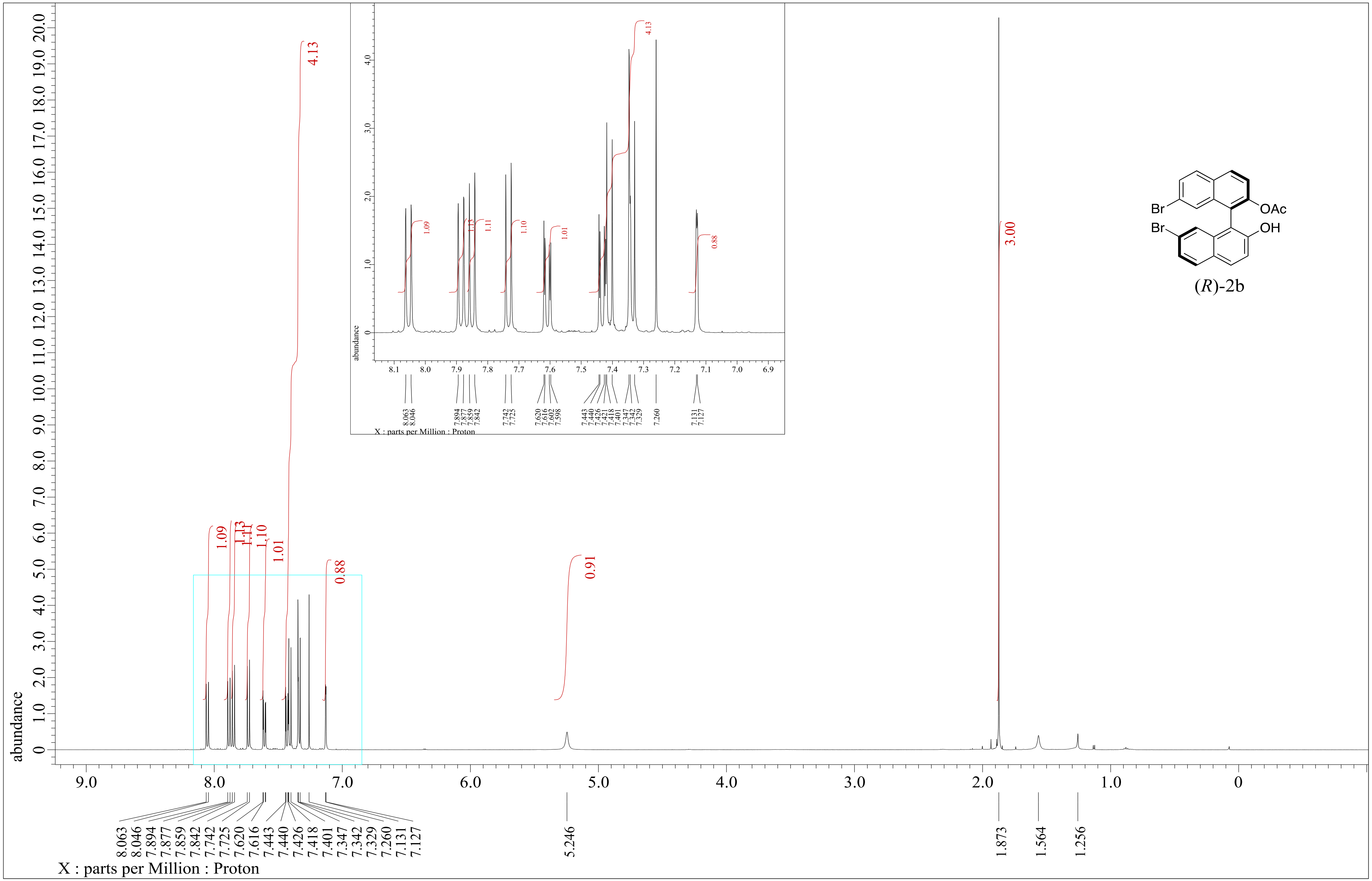

^{1}H NMR spectrum (500 MHz, $CDCl_3$) of (*R*)-**2b**

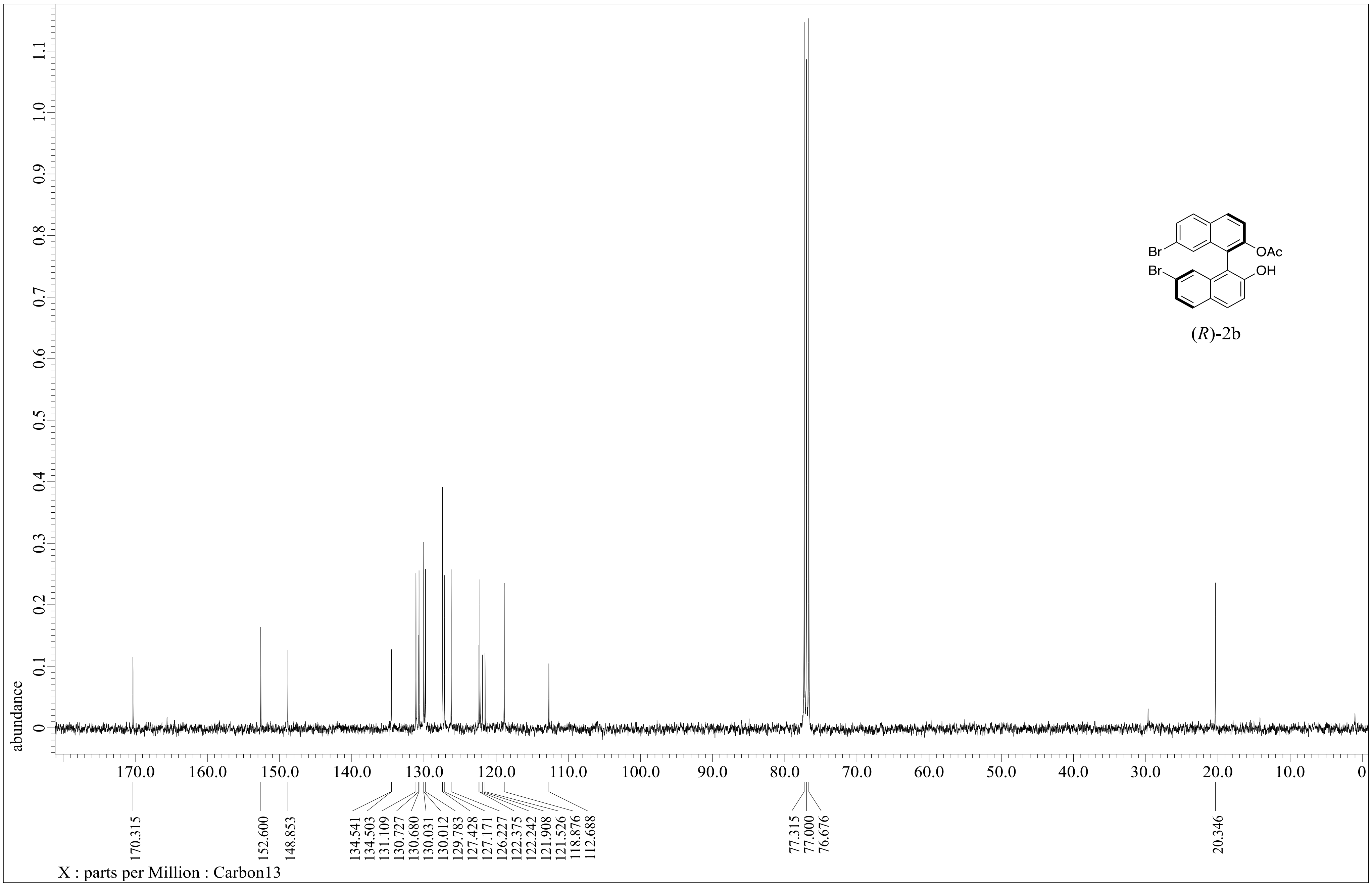

^{13}C NMR spectrum (100 MHz, $CDCl_3$) of (*R*)-**2b**

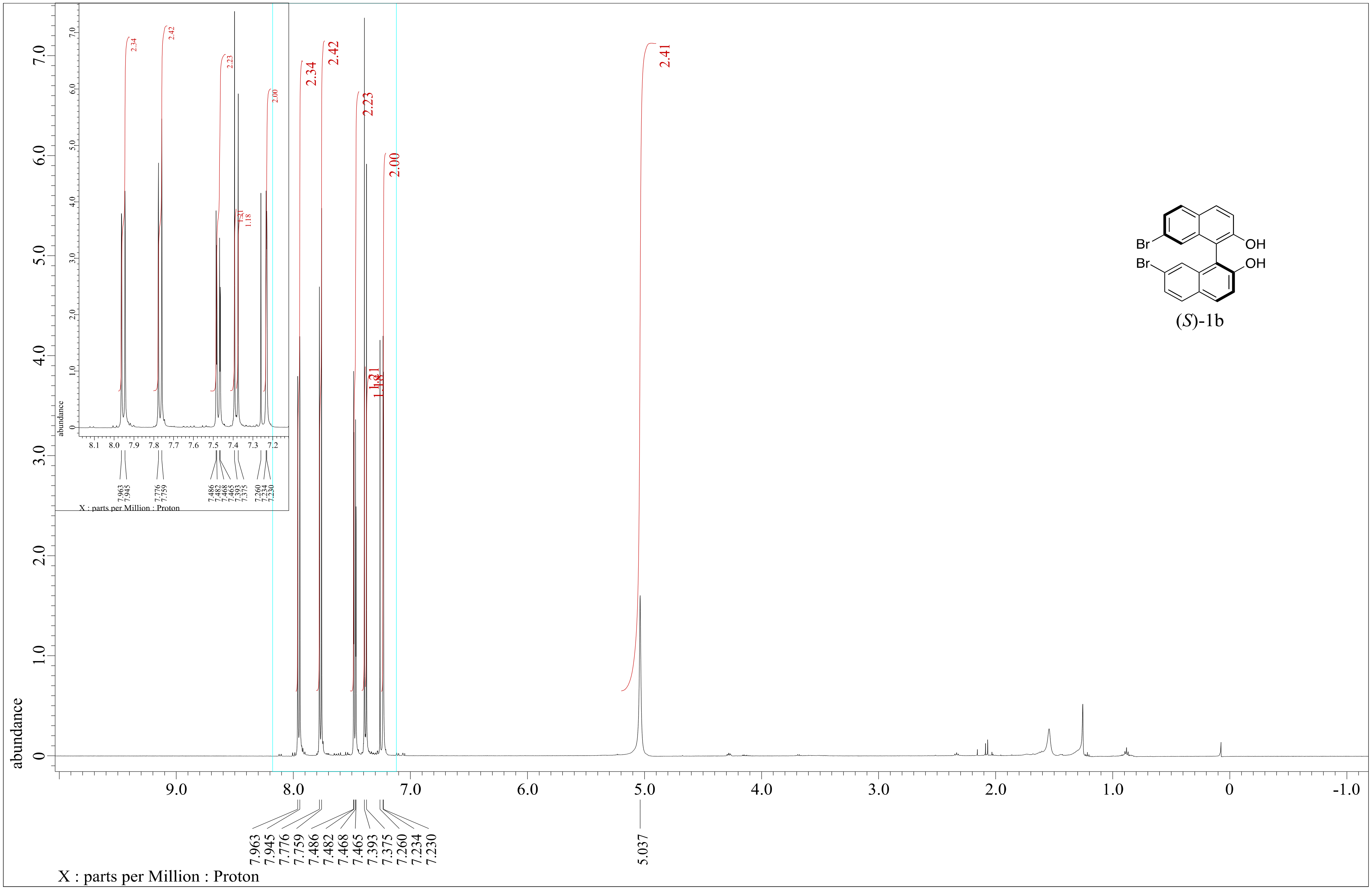

^{1}H NMR spectrum (500 MHz, $CDCl_3$) of (*S*)-**1b**

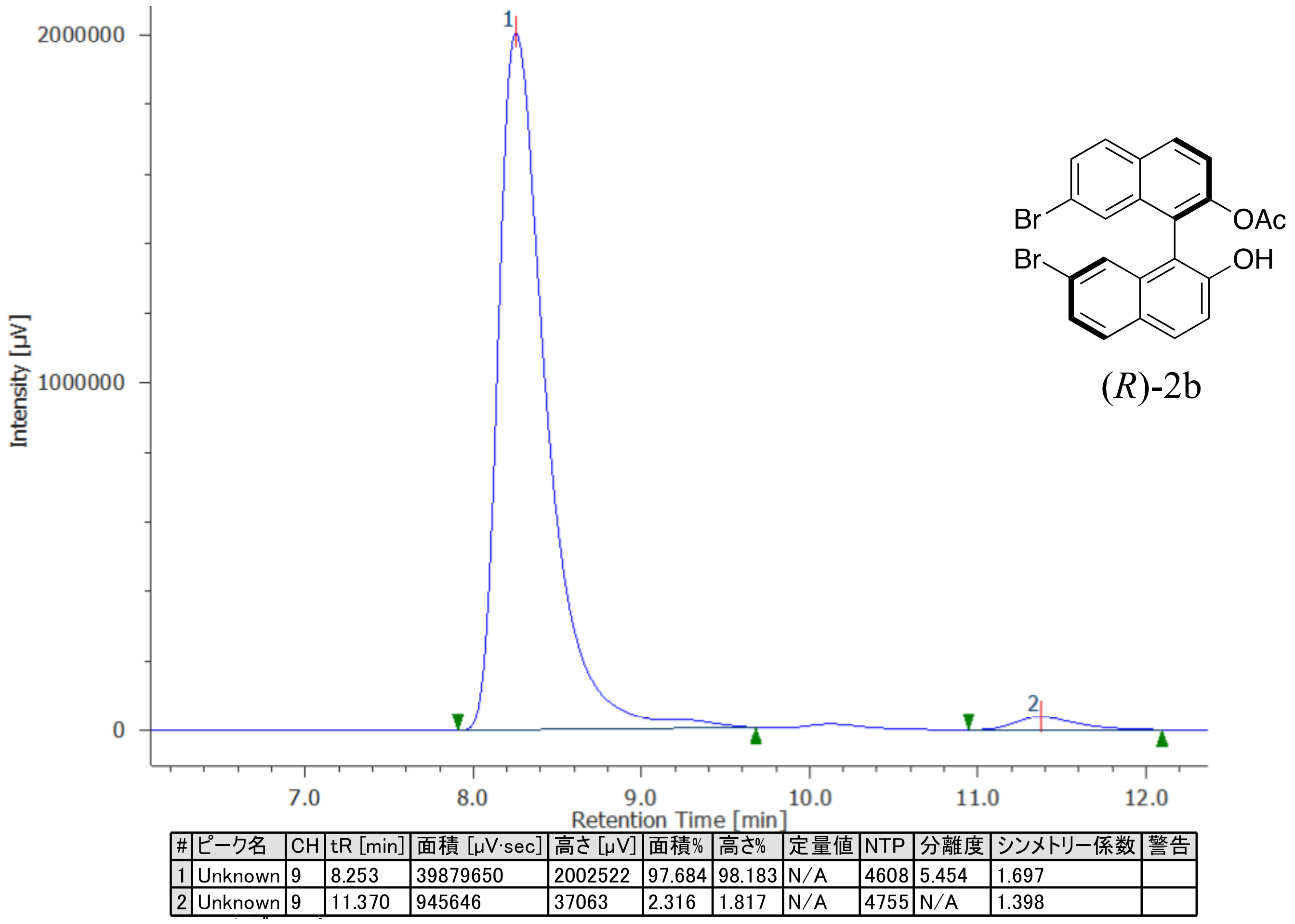

#	ピーク名	CH	tR [min]	面積 [µV·sec]	高さ [µV]	面積%	高さ%	定量値	NTP	分離度	シンメトリー係数	警告
1	Unknown	9	8.253	39879650	2002522	97.684	98.183	N/A	4608	5.454	1.697	
2	Unknown	9	11.370	945646	37063	2.316	1.817	N/A	4755	N/A	1.398	

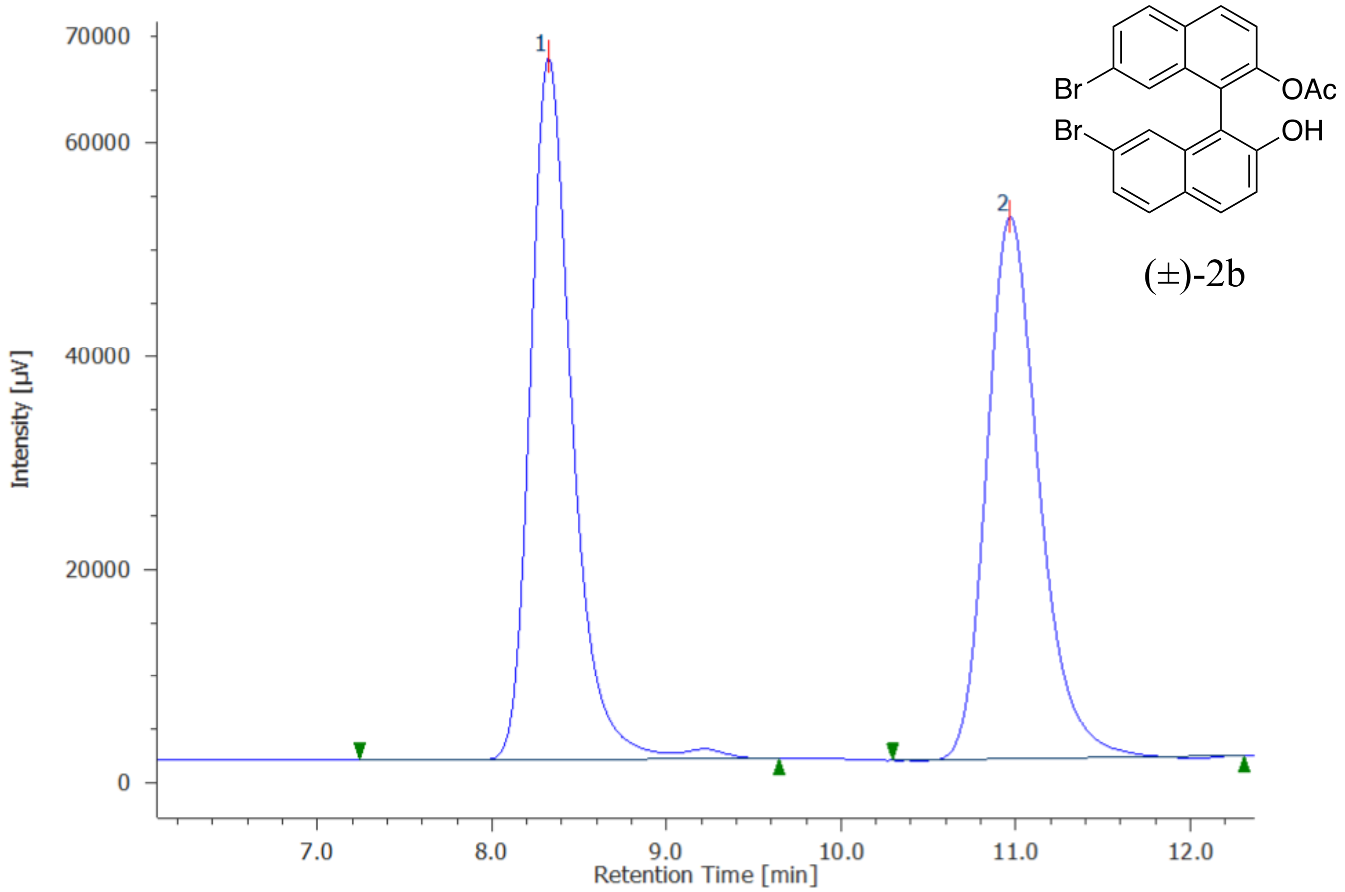

#	ピーク名	CH	tR [min]	面積 [µV·sec]	高さ [µV]	面積%	高さ%	定量値	NTP	分離度	シンメトリー係数	警告
1	Unknown	9	8.323	1101313	65835	51.011	56.412	N/A	6419	5.598	1.315	
2	Unknown	9	10.967	1057641	50870	48.989	43.588	N/A	6815	N/A	1.263	

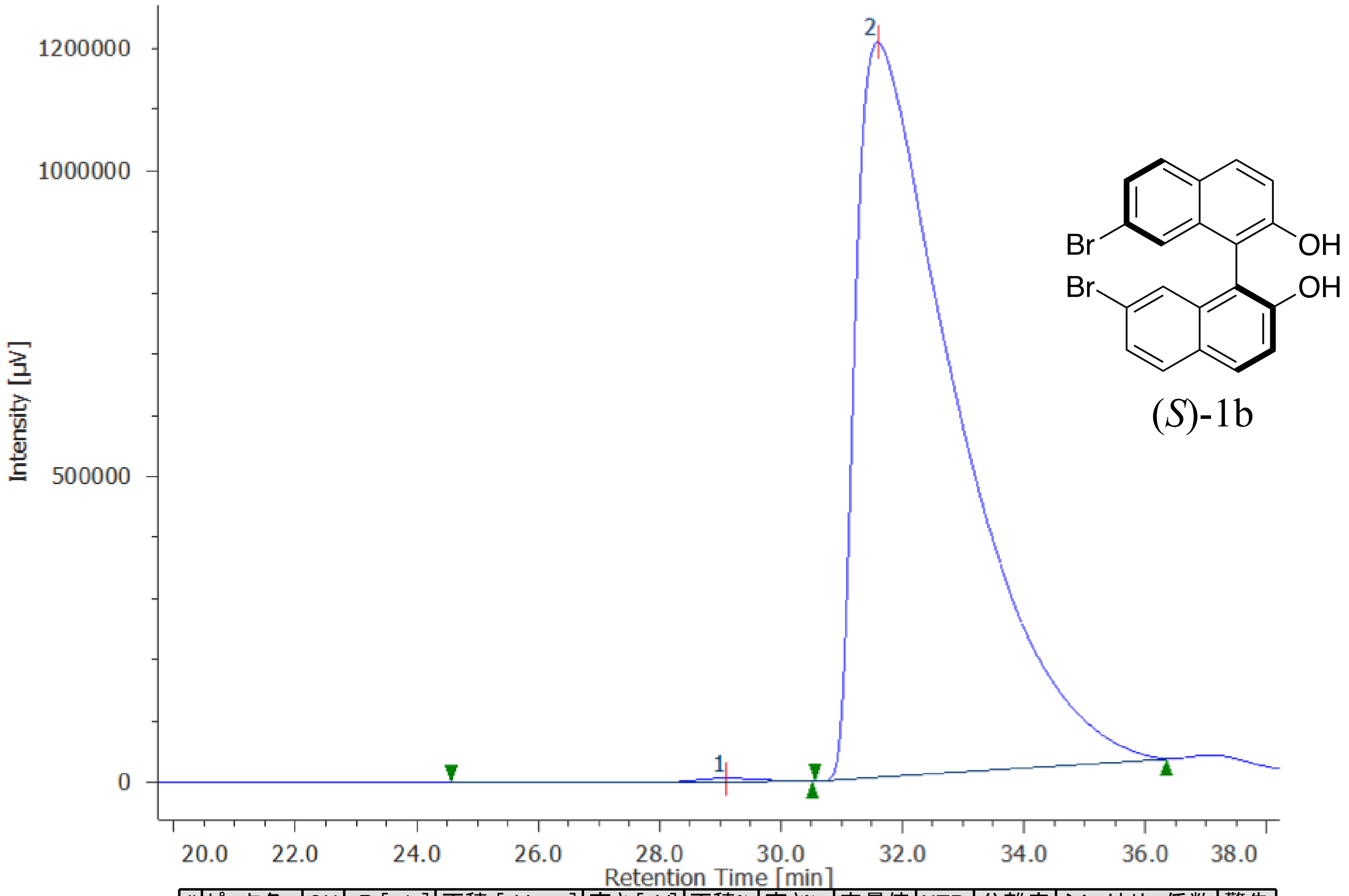

(*S*)-1b

#	ピーク名	CH	tR [min]	面積 [µV·sec]	高さ [µV]	面積%	高さ%	定量値	NTP	分離度	シンメトリー係数	警告
1	Unknown	9	29.103	381629	5410	0.273	0.448	N/A	3447	1.013	1.285	
2	Unknown	9	31.597	139264406	1201835	99.727	99.552	N/A	1832	N/A	3.265	

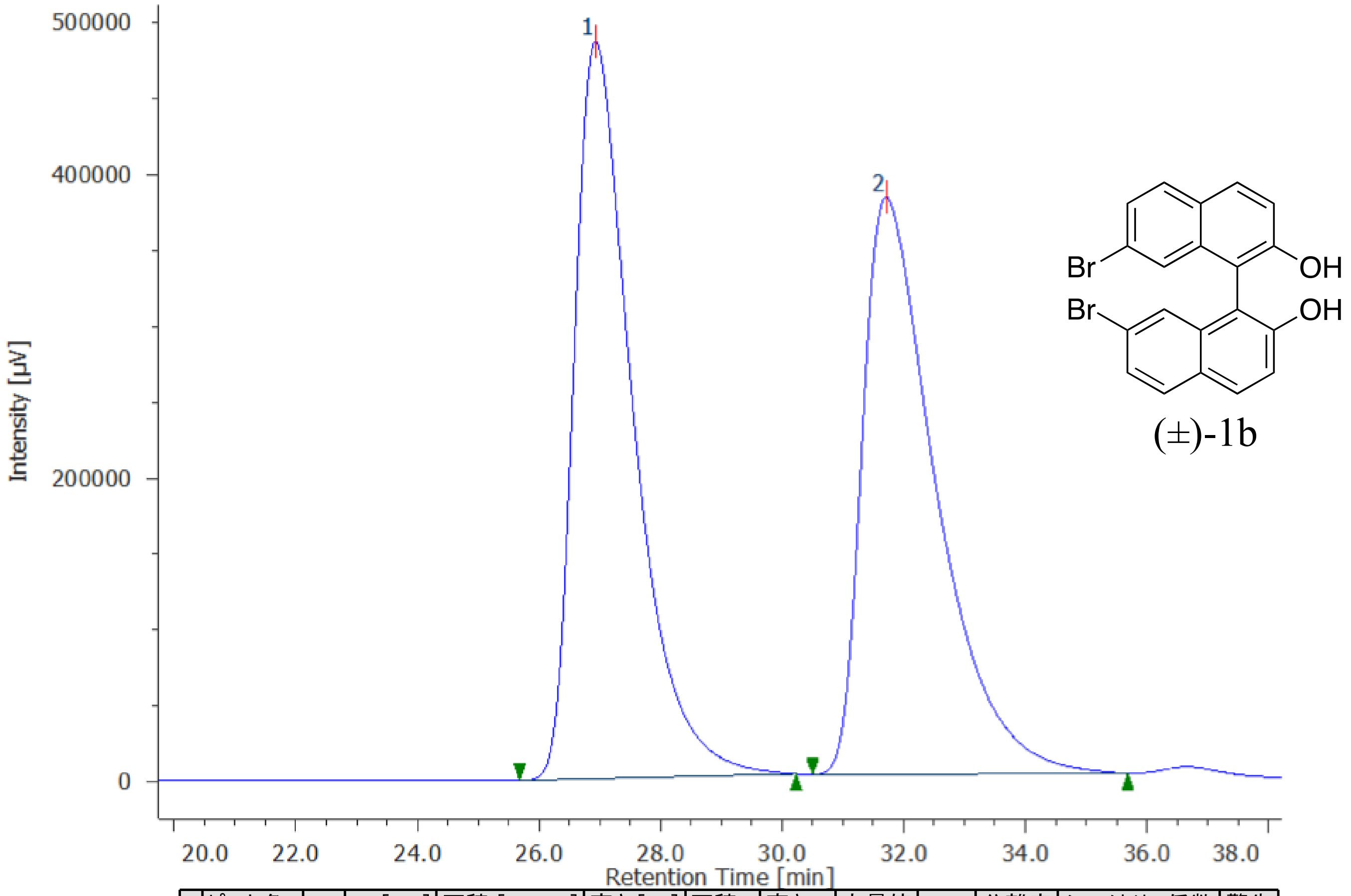

(±)-1b

#	ピーク名	CH	tR [min]	面積 [µV·sec]	高さ [µV]	面積%	高さ%	定量値	NTP	分離度	シンメトリー係数	警告
1	Unknown	9	26.930	32735045	484797	50.574	56.087	N/A	3988	2.501	1.689	
2	Unknown	9	31.713	31991547	379567	49.426	43.913	N/A	3548	N/A	1.924	

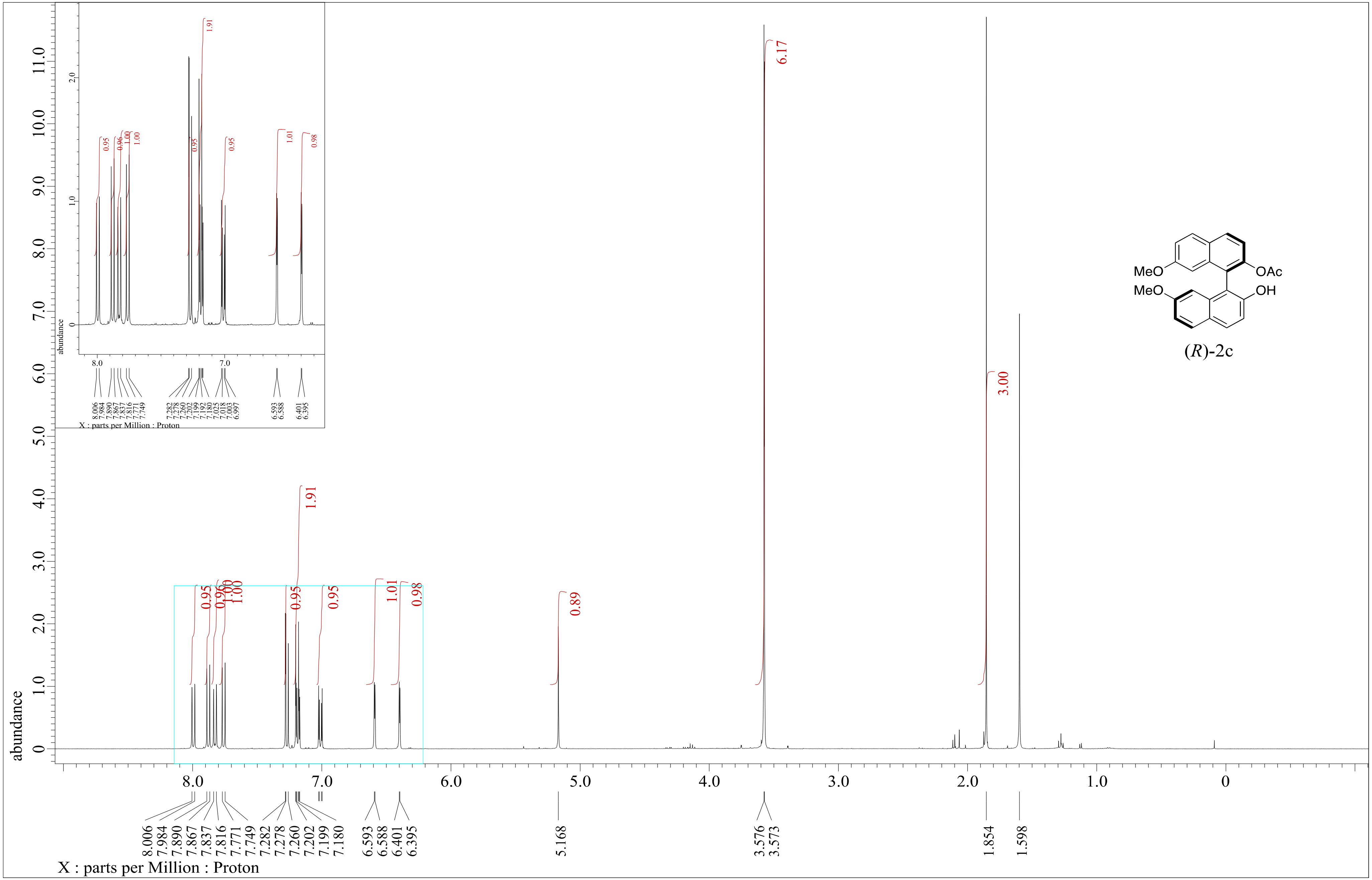

^{1}H NMR spectrum (400 MHz, $CDCl_3$) of (*R*)-**2c**

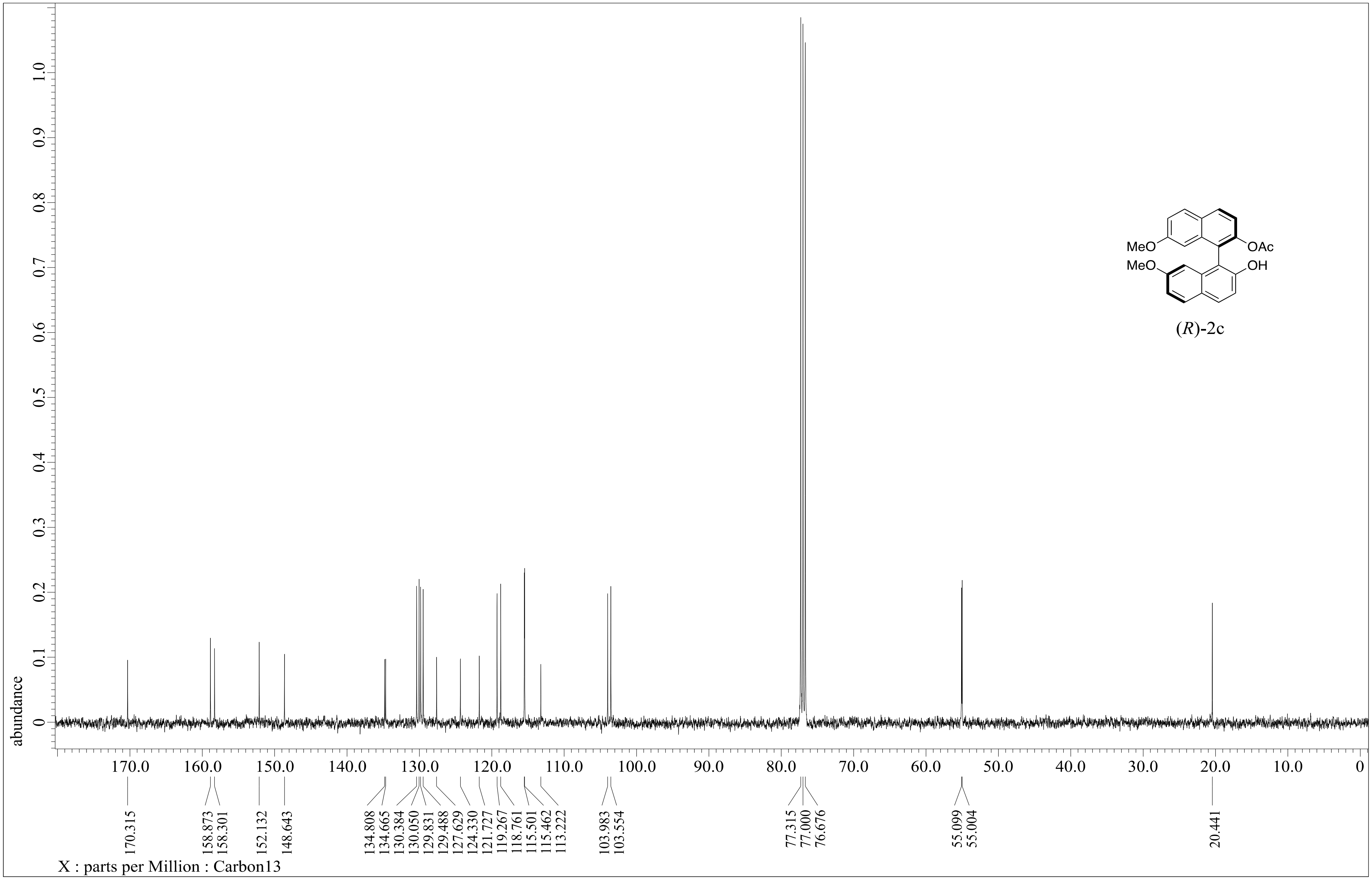

^{13}C NMR spectrum (100 MHz, $CDCl_3$) of (*R*)-**2c**

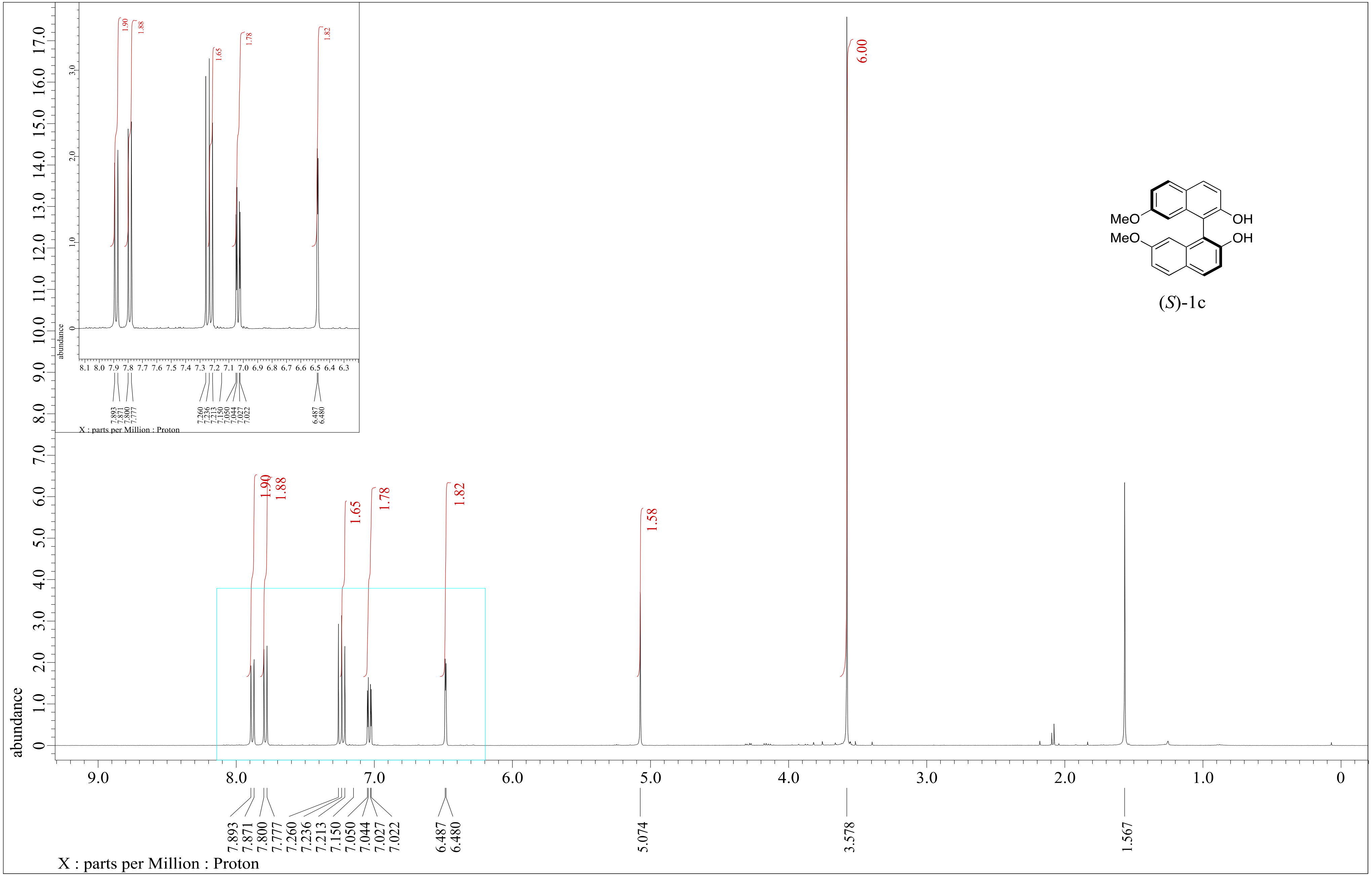

^{1}H NMR spectrum (400 MHz, $CDCl_3$) of (*S*)-**1c**

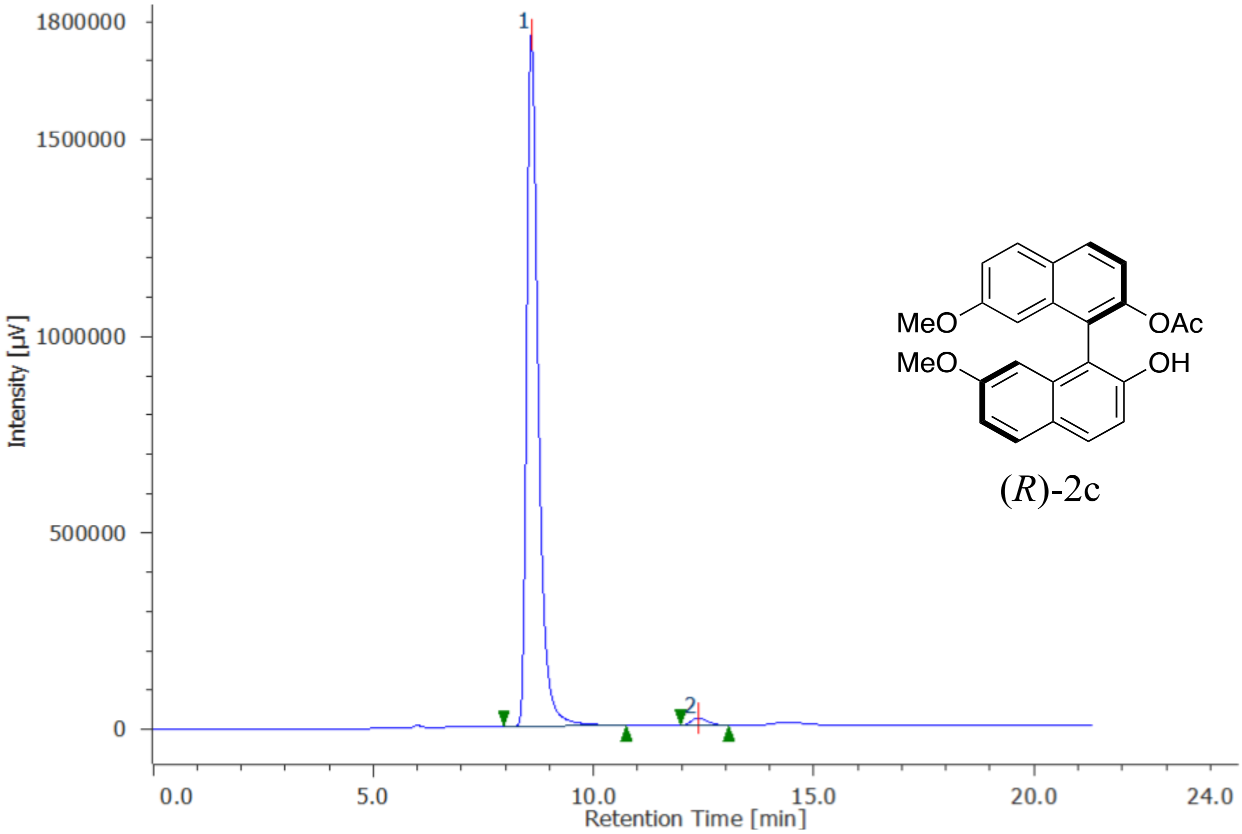

#	ピーク名	CH	tR [min]	面積 [µV·sec]	高さ [µV]	面積%	高さ%	定量値	NTP	分離度	シンメトリー係数	警告
1	Unknown	9	8.587	32434818	1758278	98.533	98.943	N/A	5669	6.721	1.527	
2	Unknown	9	12.380	482909	18788	1.467	1.057	N/A	5371	N/A	1.279	

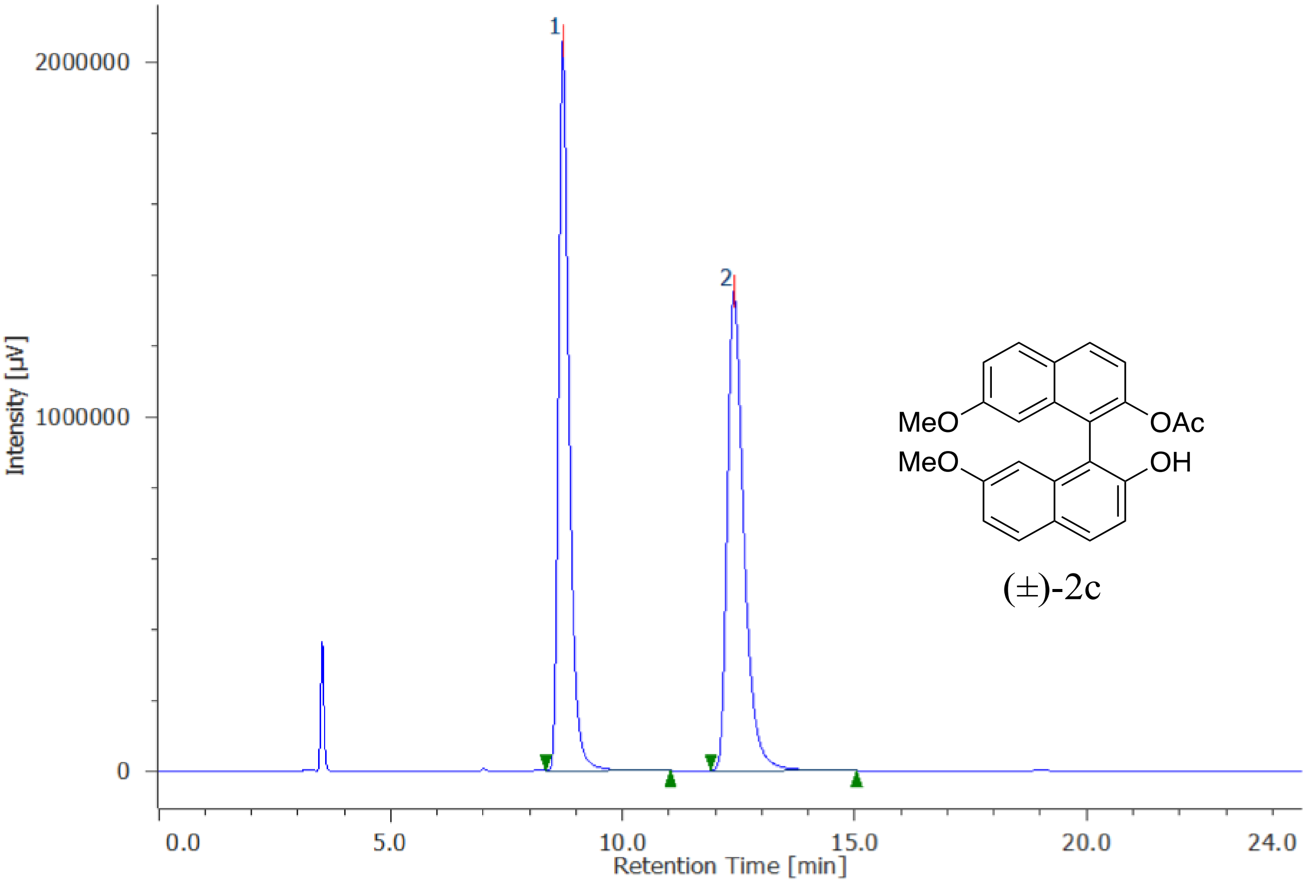

#	ピーク名	CH	tR [min]	面積 [µV·sec]	高さ [µV]	面積%	高さ%	定量値	NTP	分離度	シンメトリー係数	警告
1	Unknown	9	8.710	32896703	2056299	49.457	60.358	N/A	7646	7.240	1.449	
2	Unknown	9	12.397	33619093	1350533	50.543	39.642	N/A	6340	N/A	1.496	

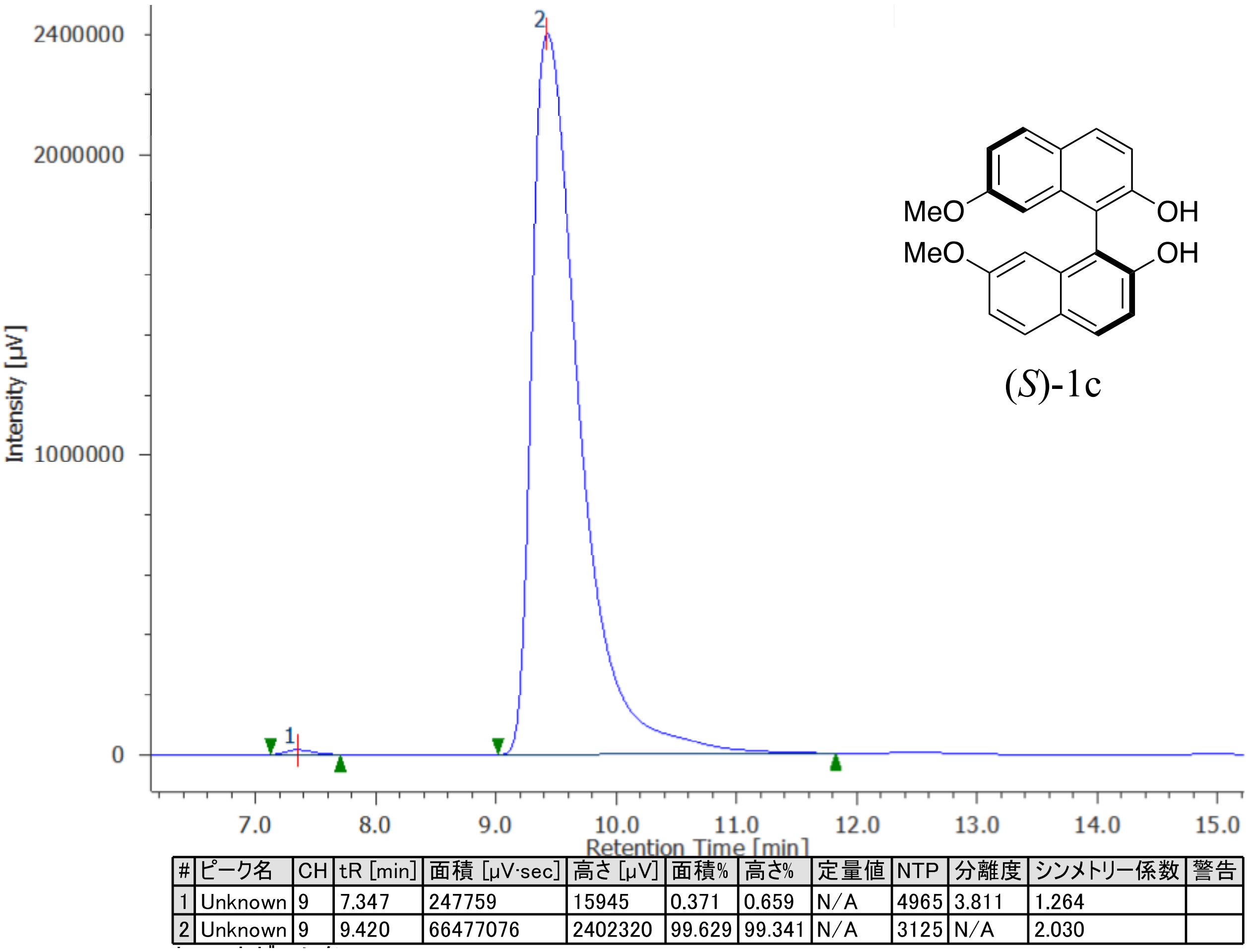

#	ピーク名	CH	tR [min]	面積 [µV·sec]	高さ [µV]	面積%	高さ%	定量値	NTP	分離度	シンメトリー係数	警告
1	Unknown	9	7.347	247759	15945	0.371	0.659	N/A	4965	3.811	1.264	
2	Unknown	9	9.420	66477076	2402320	99.629	99.341	N/A	3125	N/A	2.030	

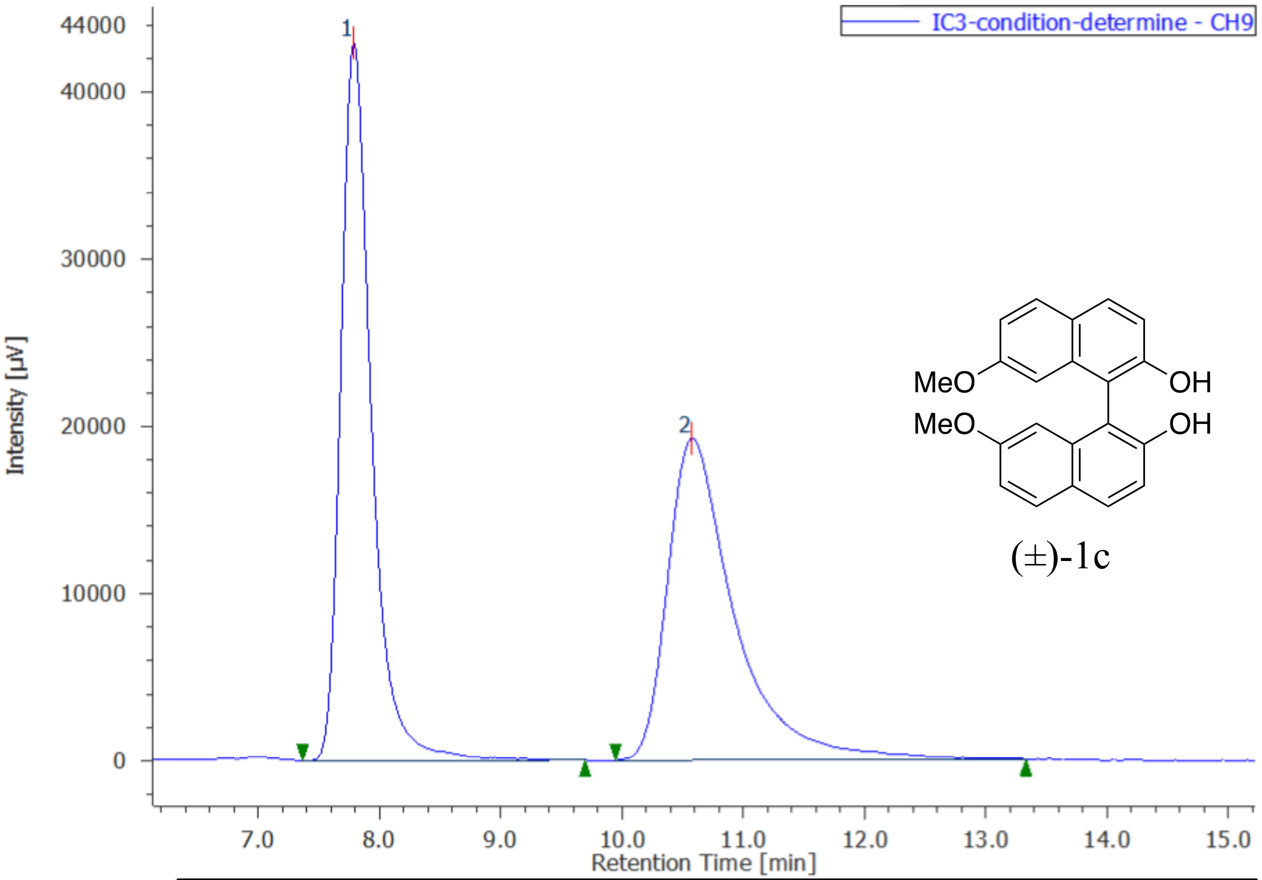

#	ピーク名	CH	tR [min]	面積 [µV·sec]	高さ [µV]	面積%	高さ%	定量値	NTP	分離度	シンメトリー係数	警告
1	Unknown	9	7.787	754884	42931	50.542	69.048	N/A	5170	4.160	1.379	
2	Unknown	9	10.573	738702	19245	49.458	30.952	N/A	2159	N/A	1.794	

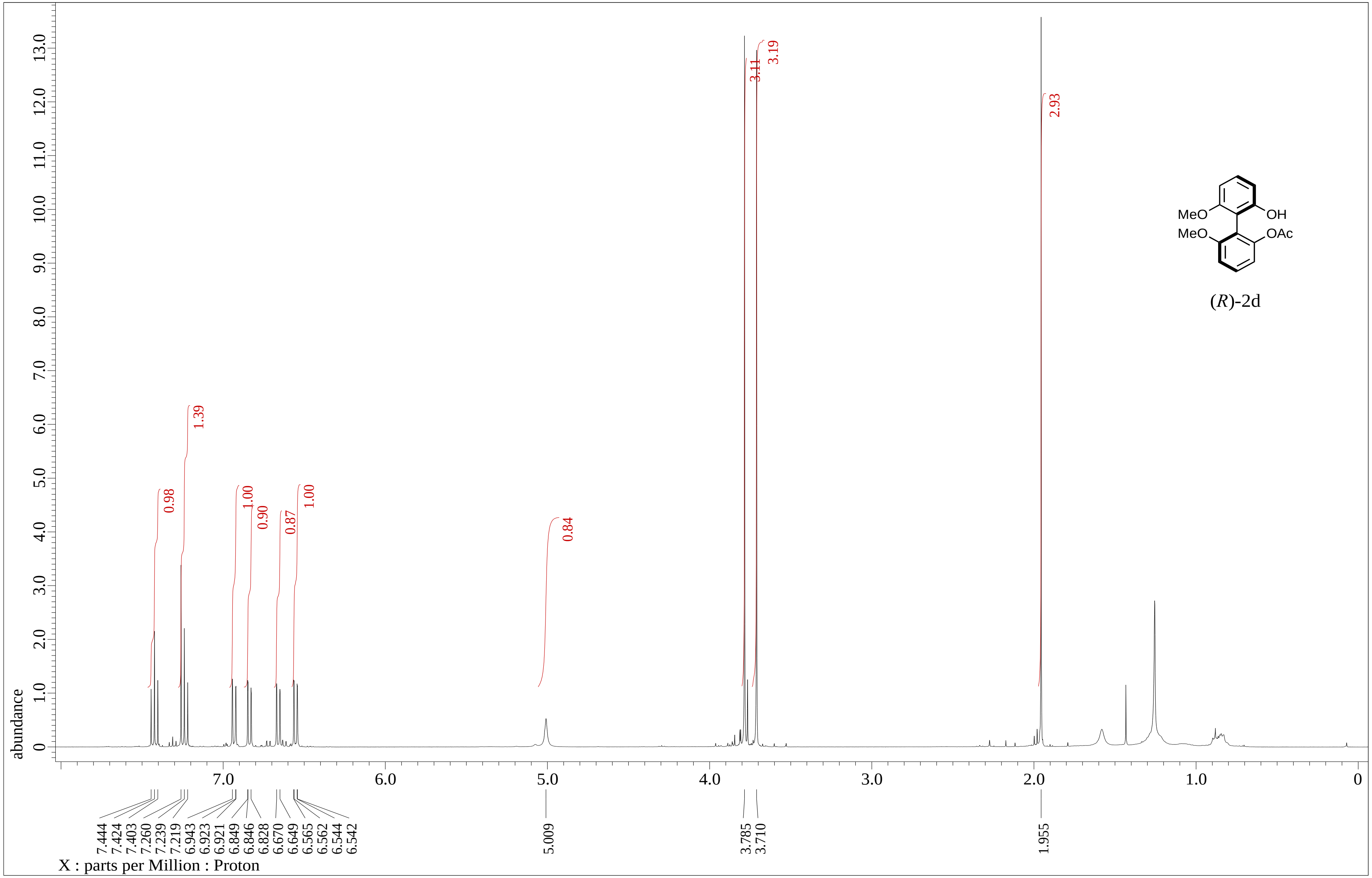

^{1}H NMR spectrum (400 MHz, $CDCl_3$) of (*R*)-**2d**

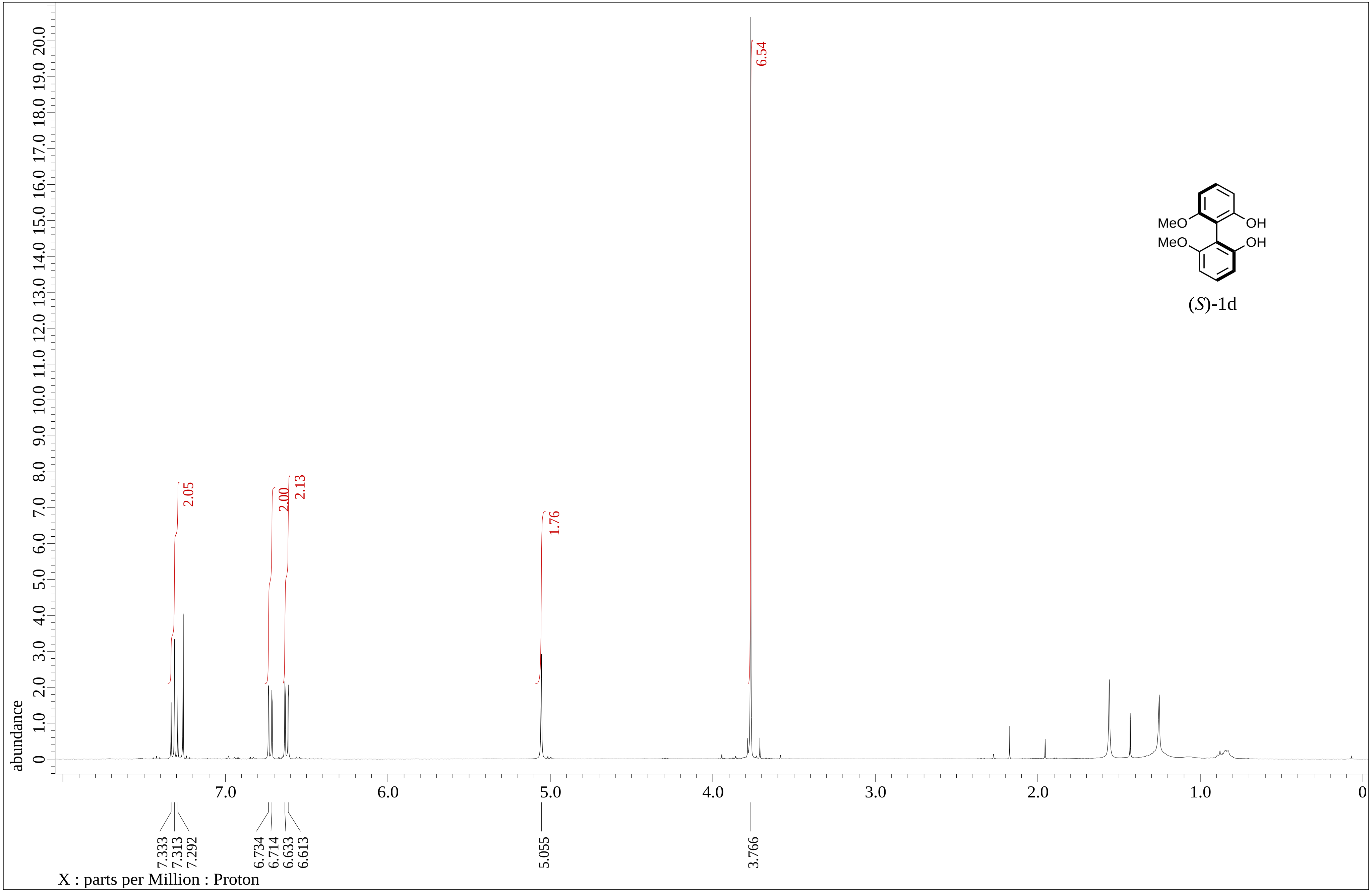

^{1}H NMR spectra (400 MHz, $CDCl_3$) of (*S*)-**1d**

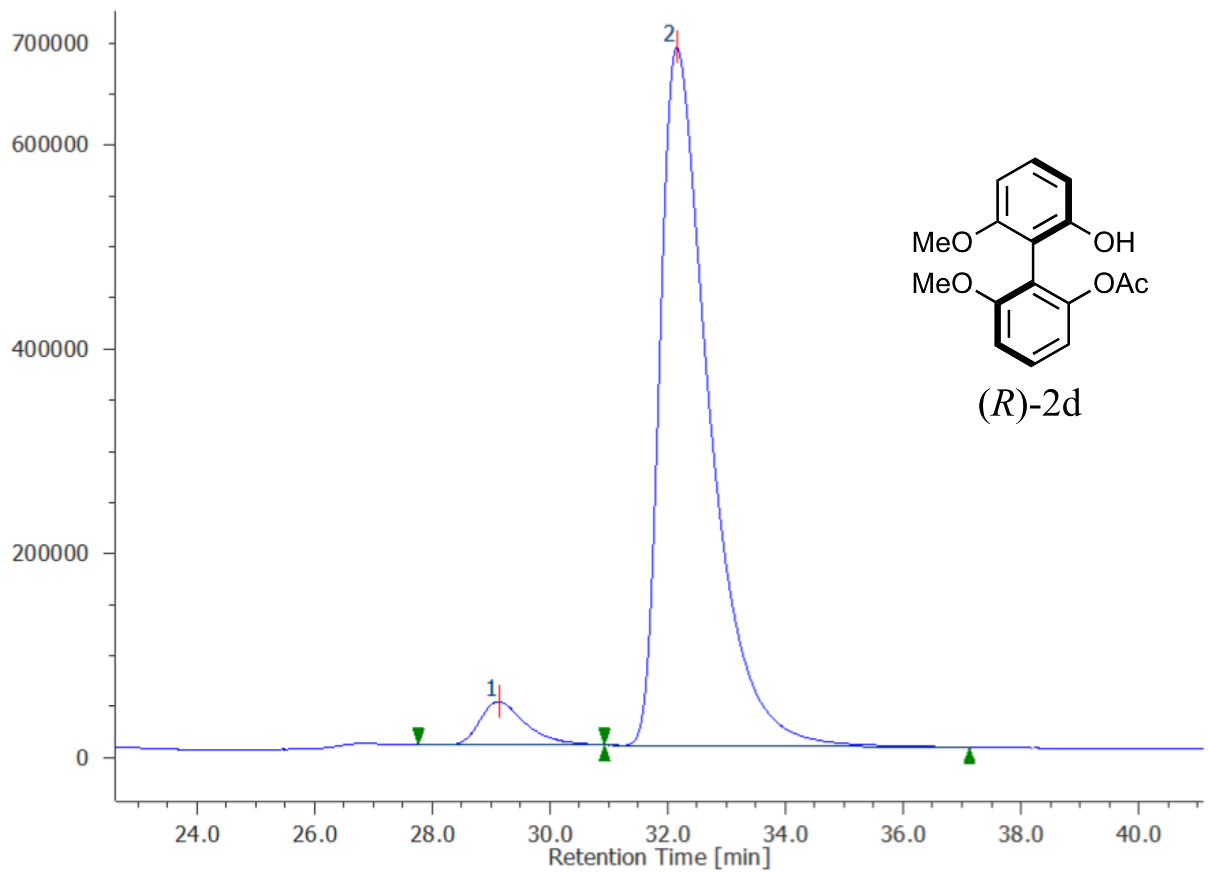

#	ピーク名	CH	tR [min]	面積 [μV·sec]	高さ [μV]	面積%	高さ%	定量値	NTP	分離度	シンメトリー係数	警告
1	Unknown	9	29.127	2278319	42397	5.427	5.843	N/A	7390	2.161	1.456	
2	Unknown	9	32.150	39700334	683229	94.573	94.157	N/A	7867	N/A	1.834	

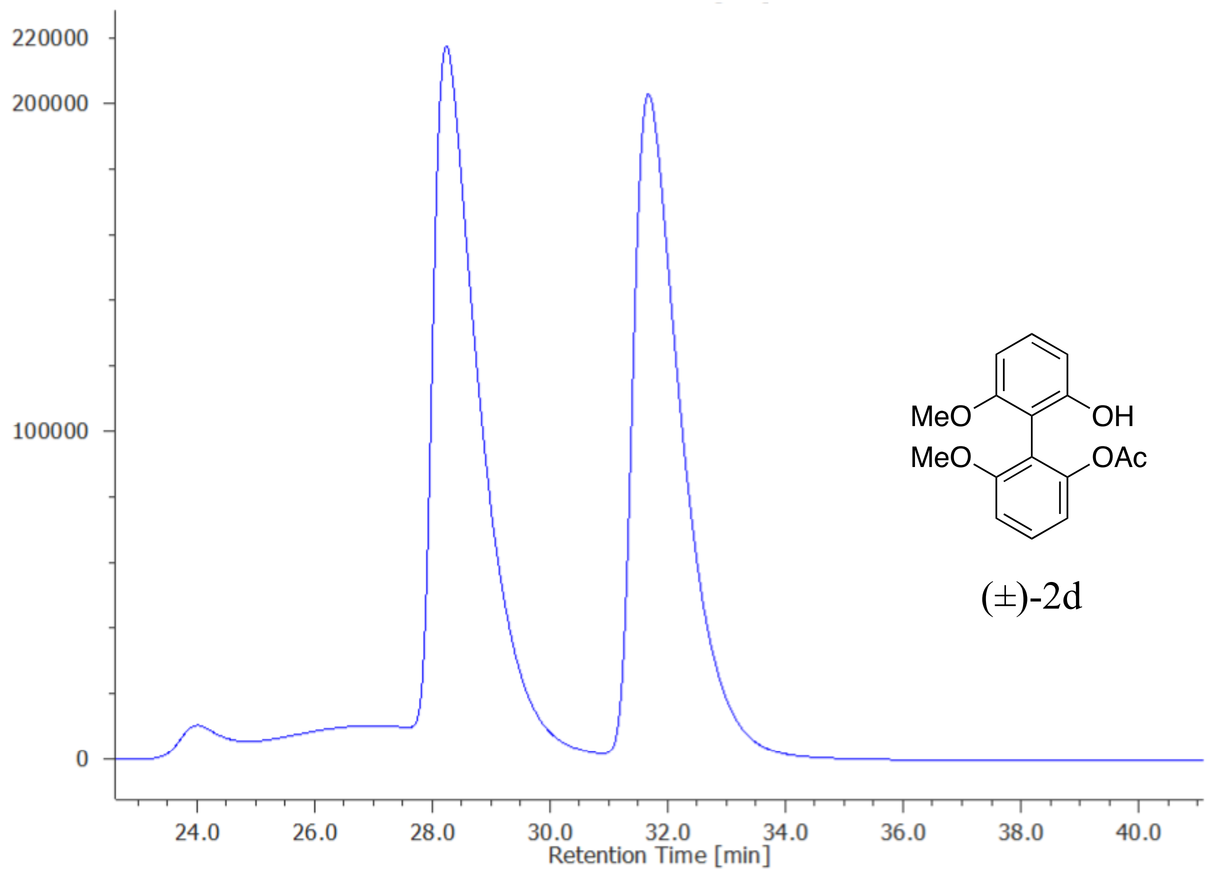

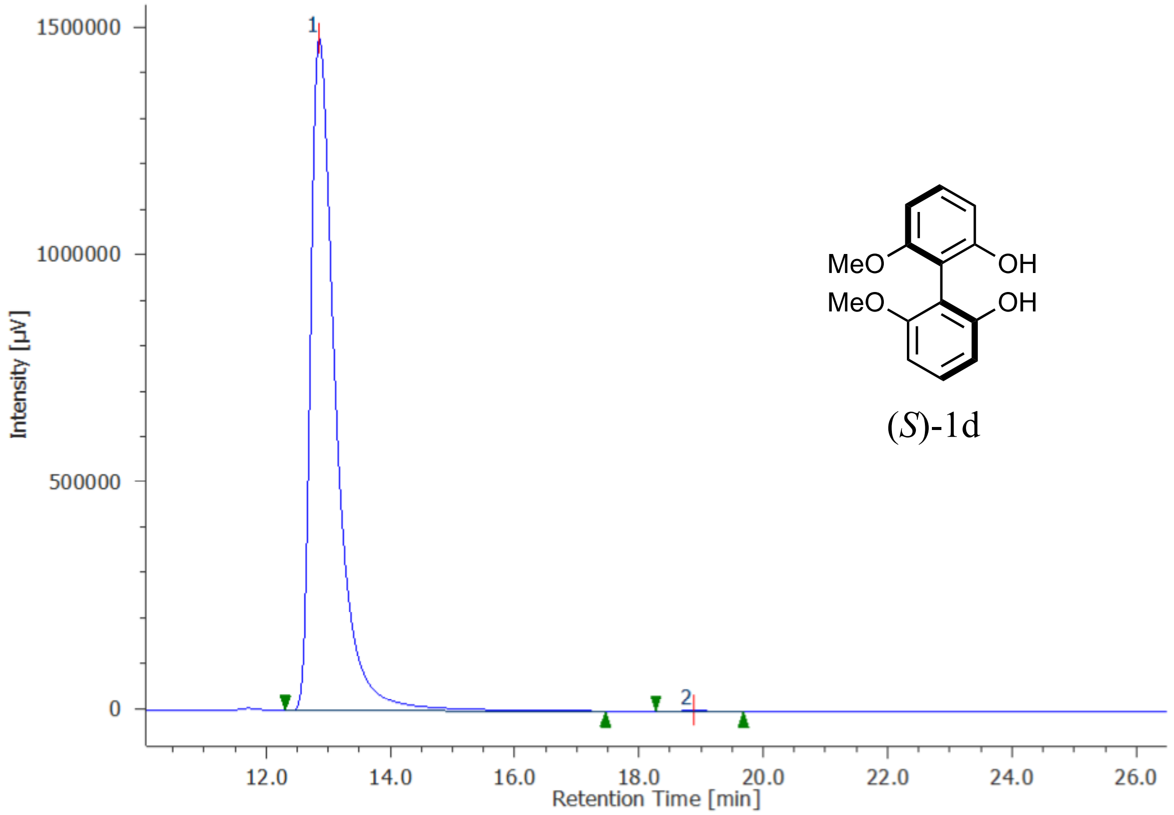

#	ピーク名	CH	tR [min]	面積 [µV·sec]	高さ [µV]	面積%	高さ%	定量値	NTP	分離度	シンメトリー係数	警告
1	Unknown	9	12.857	41595458	1480102	99.899	99.923	N/A	5713	7.239	1.825	
2	Unknown	9	18.877	42121	1140	0.101	0.077	N/A	5850	N/A	1.169	

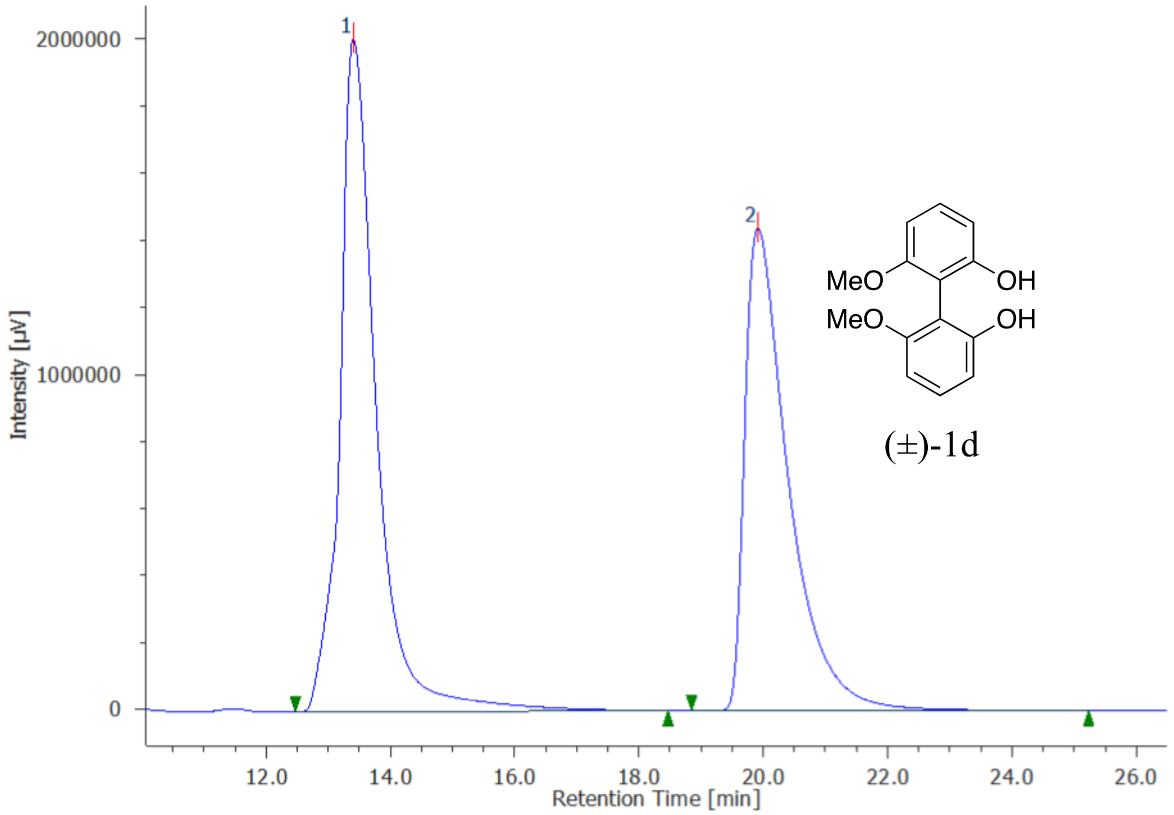

#	ピーク名	CH	tR [min]	面積 [µV·sec]	高さ [µV]	面積%	高さ%	定量値	NTP	分離度	シンメトリー係数	警告
1	Unknown	9	13.403	81456823	2006547	53.731	58.230	N/A	3502	6.203	1.312	
2	Unknown	9	19.913	70143878	1439324	46.269	41.770	N/A	4416	N/A	2.236	

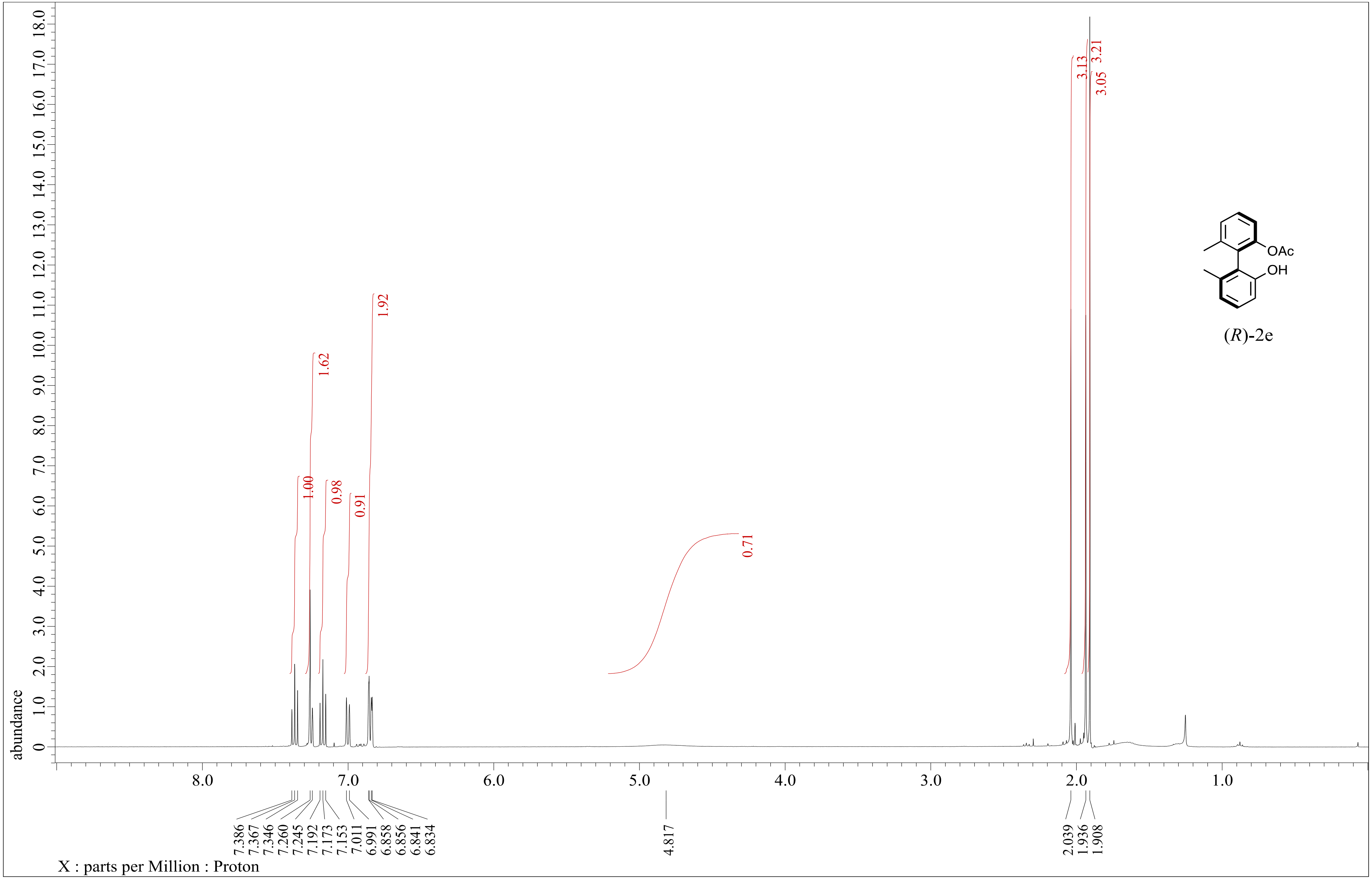

(*R*)-2e

^{1}H NMR spectrum (400 MHz, $CDCl_3$) of (*R*)-**2e**

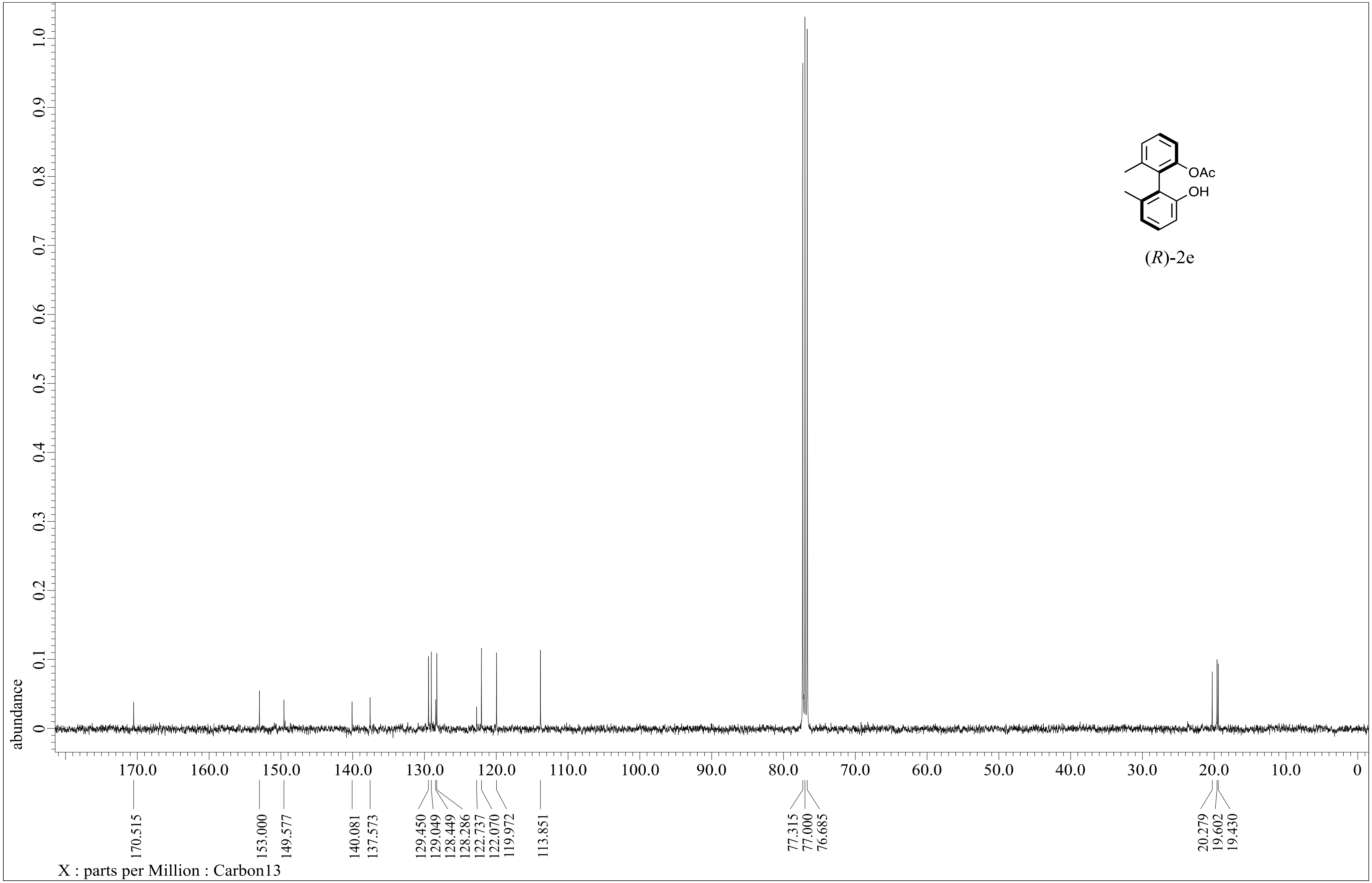

^{13}C NMR spectrum (100 MHz, $CDCl_3$) of (*R*)-**2e**

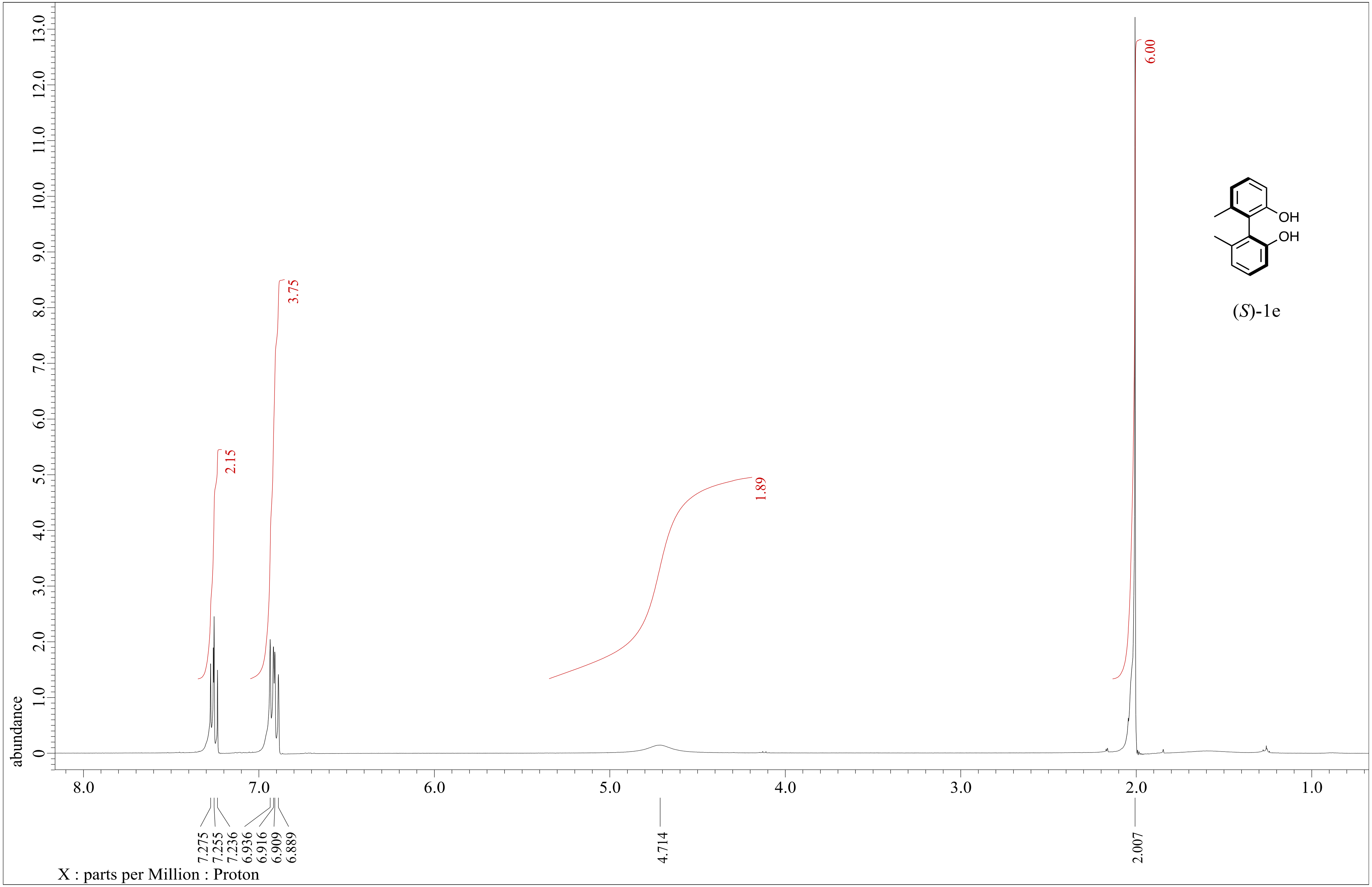

^{1}H NMR spectrum (400 MHz, $CDCl_3$) of (*S*)-**1e**

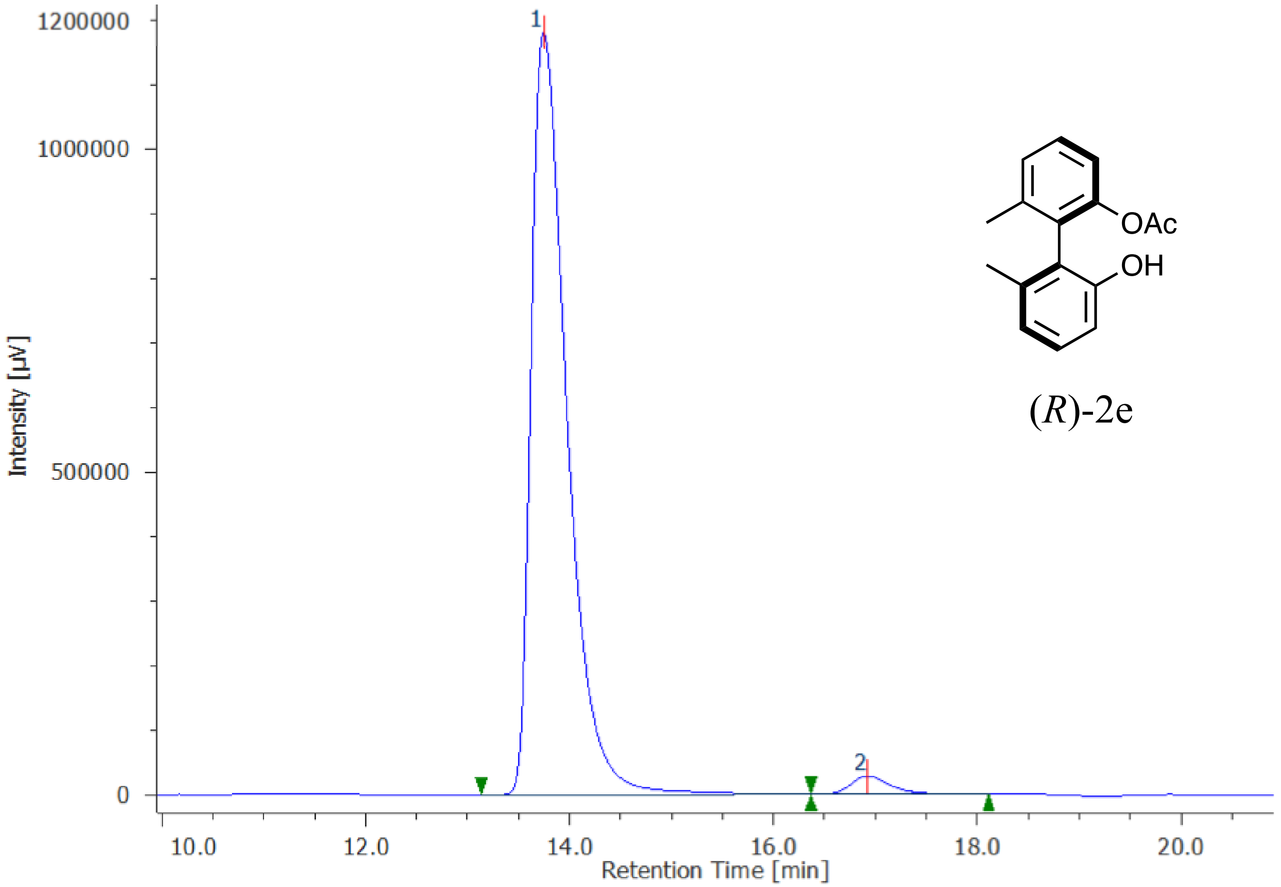

#	ピーク名	CH	tR [min]	面積 [μV·sec]	高さ [μV]	面積%	高さ%	定量値	NTP	分離度	シンメトリー係数	警告
1	Unknown	9	13.743	28930516	1180747	97.465	97.713	N/A	7870	4.904	1.753	
2	Unknown	9	16.923	752519	27641	2.535	2.287	N/A	9887	N/A	1.355	

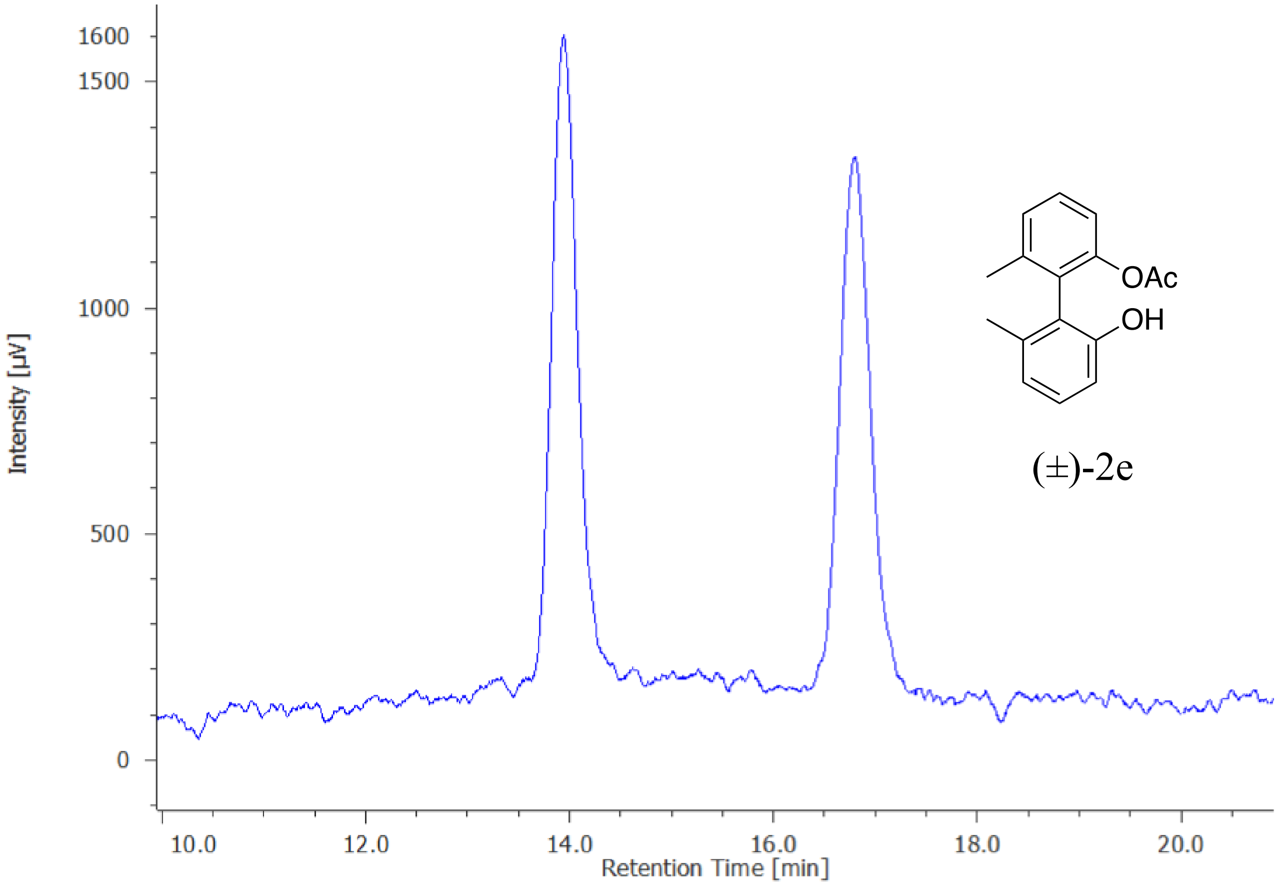

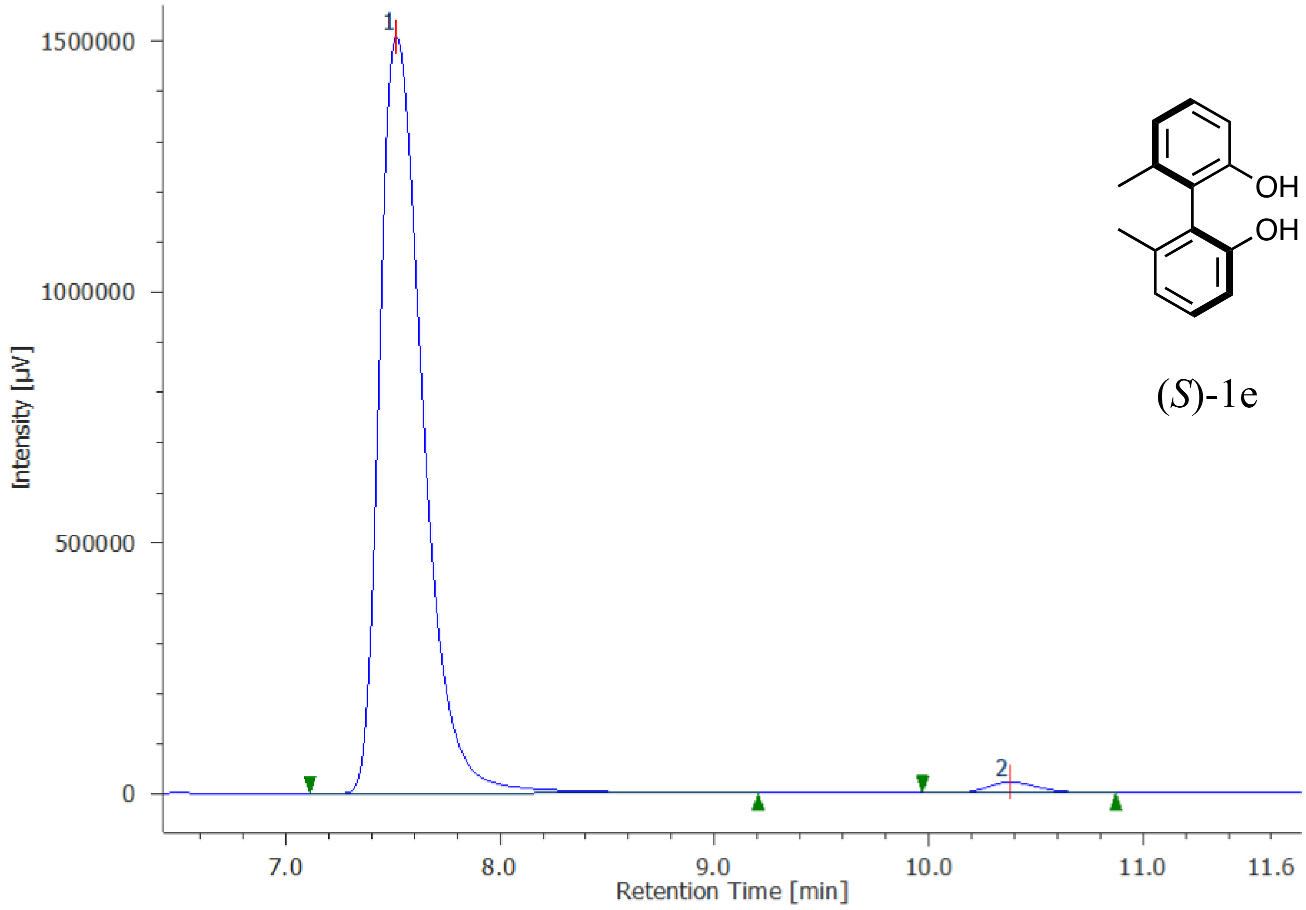

#	ピーク名	CH	tR [min]	面積 [µV·sec]	高さ [µV]	面積%	高さ%	定量値	NTP	分離度	シンメトリー係数	警告
1	Unknown	9	7.517	21189661	1507116	98.353	98.588	N/A	7035	7.304	1.470	
2	Unknown	9	10.380	354942	21582	1.647	1.412	N/A	9427	N/A	1.239	

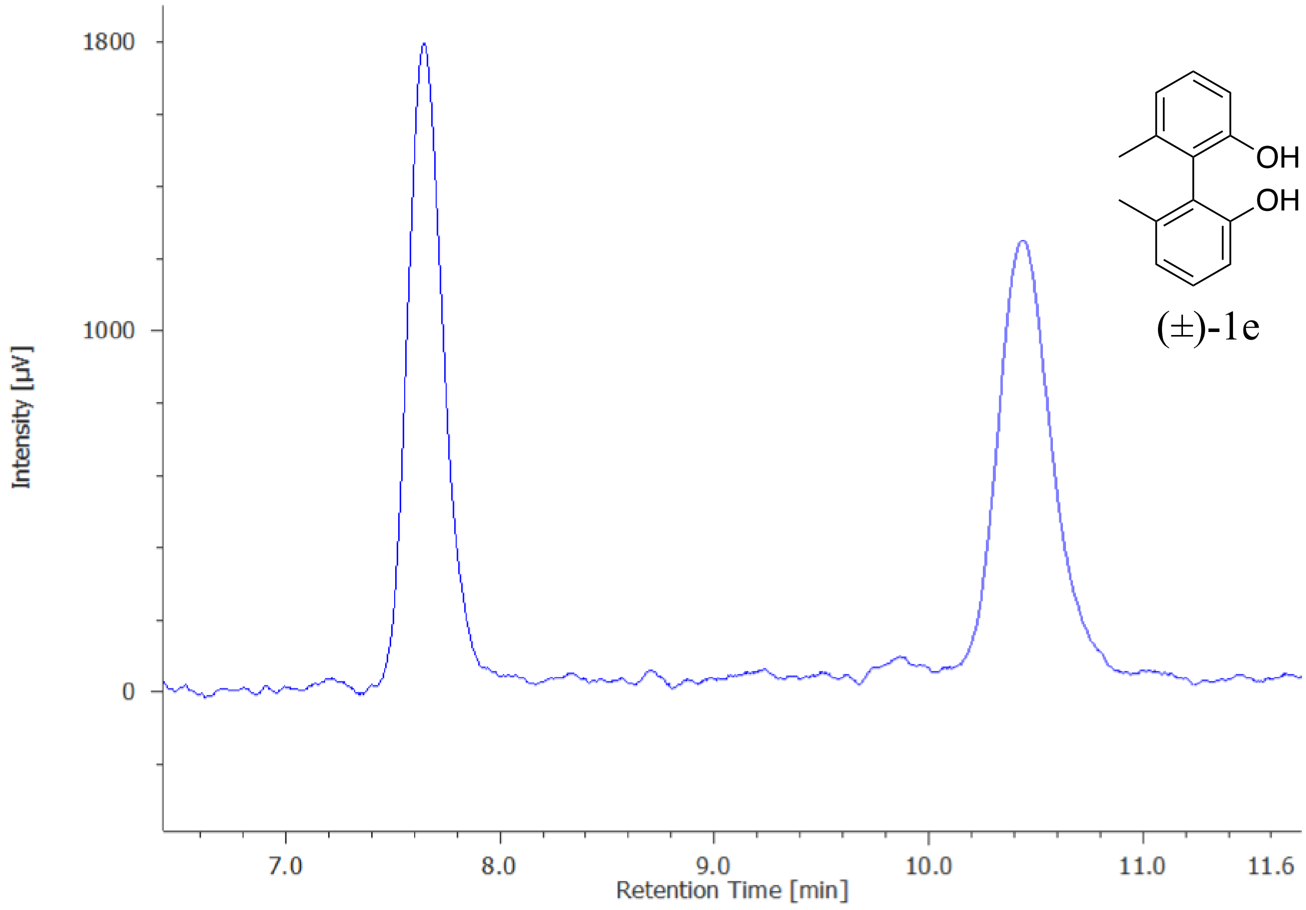

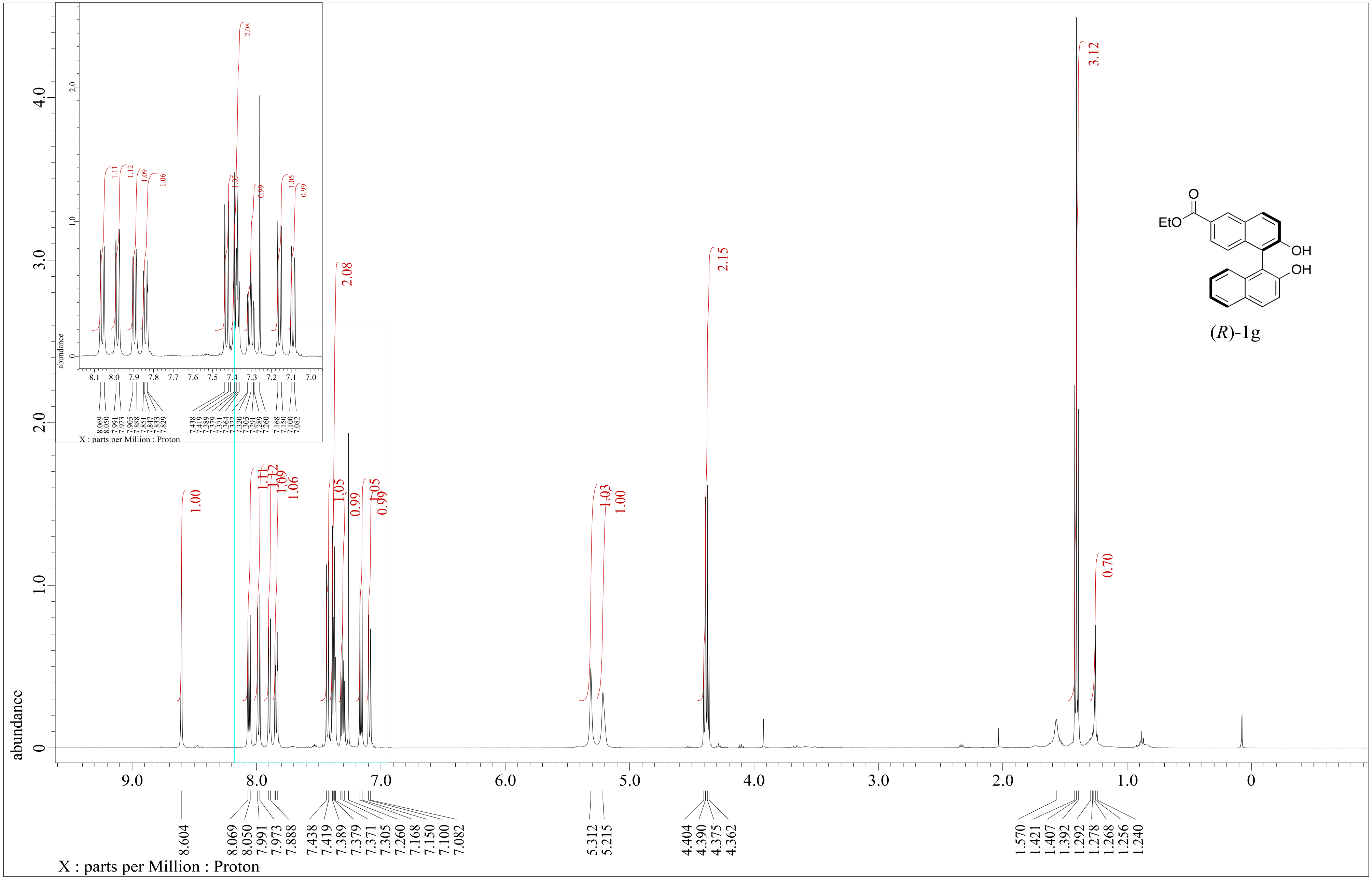

^{1}H NMR spectrum (500 MHz, $CDCl_3$) of (*R*)-**1g**

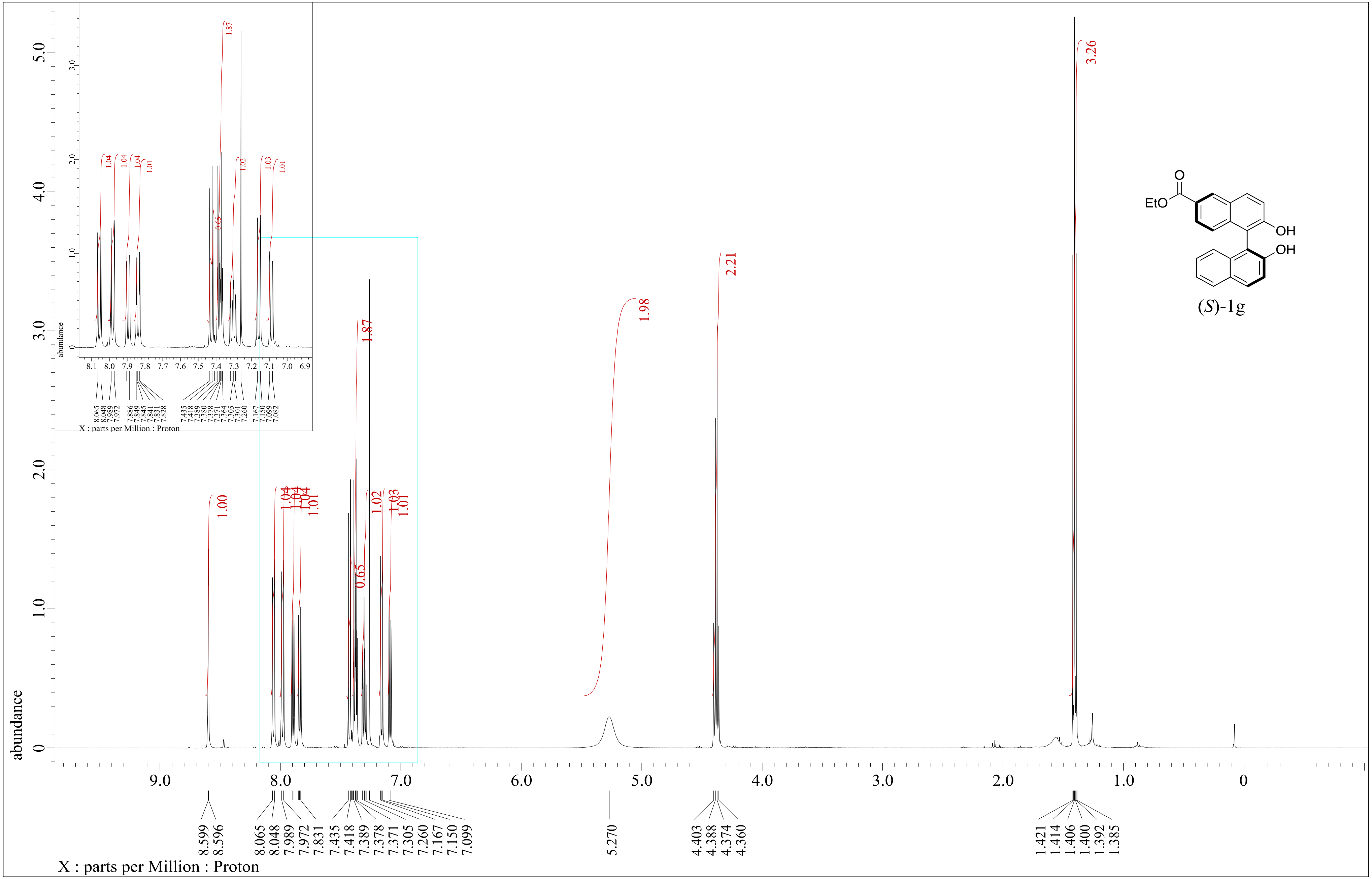

^{1}H NMR spectrum (500 MHz, $CDCl_3$) of (*S*)-**1g**

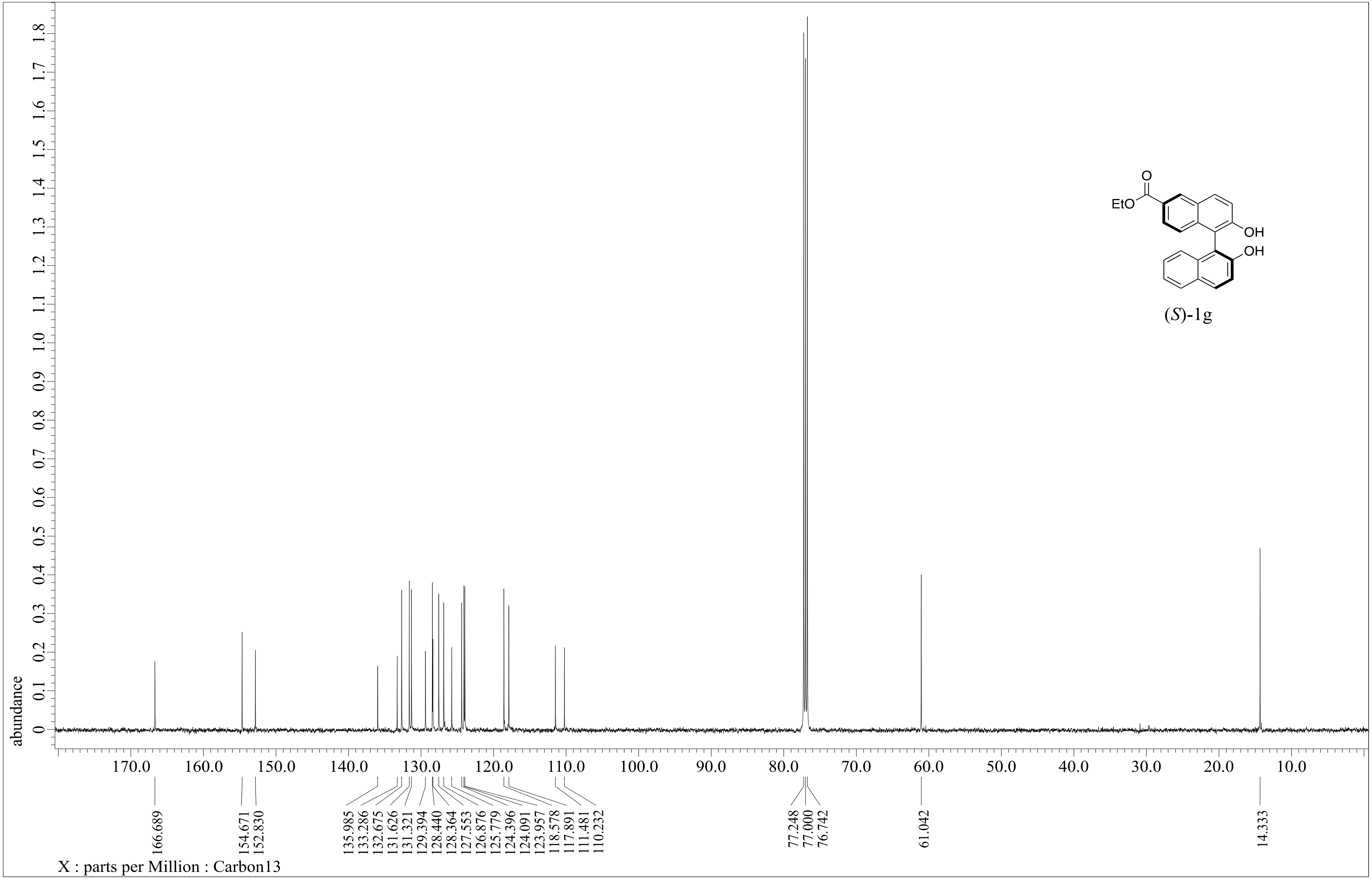

^{13}C NMR spectrum (125 MHz, $CDCl_3$) of (*S*)-**1g**

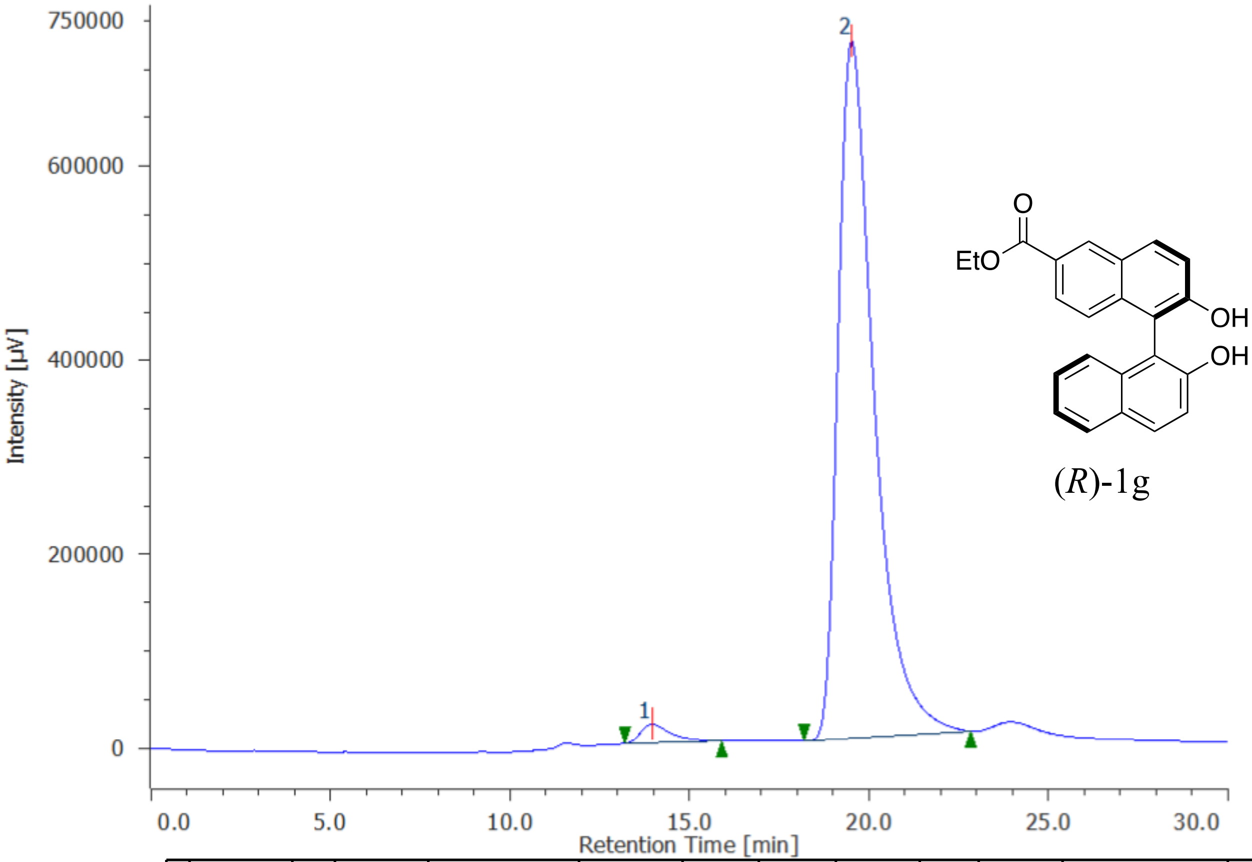

(*R*)-1g

#	ピーク名	CH	tR [min]	面積 [μV·sec]	高さ [μV]	面積%	高さ%	定量値	NTP	分離度	シンメトリー係数	警告
1	Unknown	9	13.967	1106405	19053	2.155	2.580	N/A	1477	3.484	1.638	
2	Unknown	9	19.520	50245907	719391	97.845	97.420	N/A	2006	N/A	1.678	

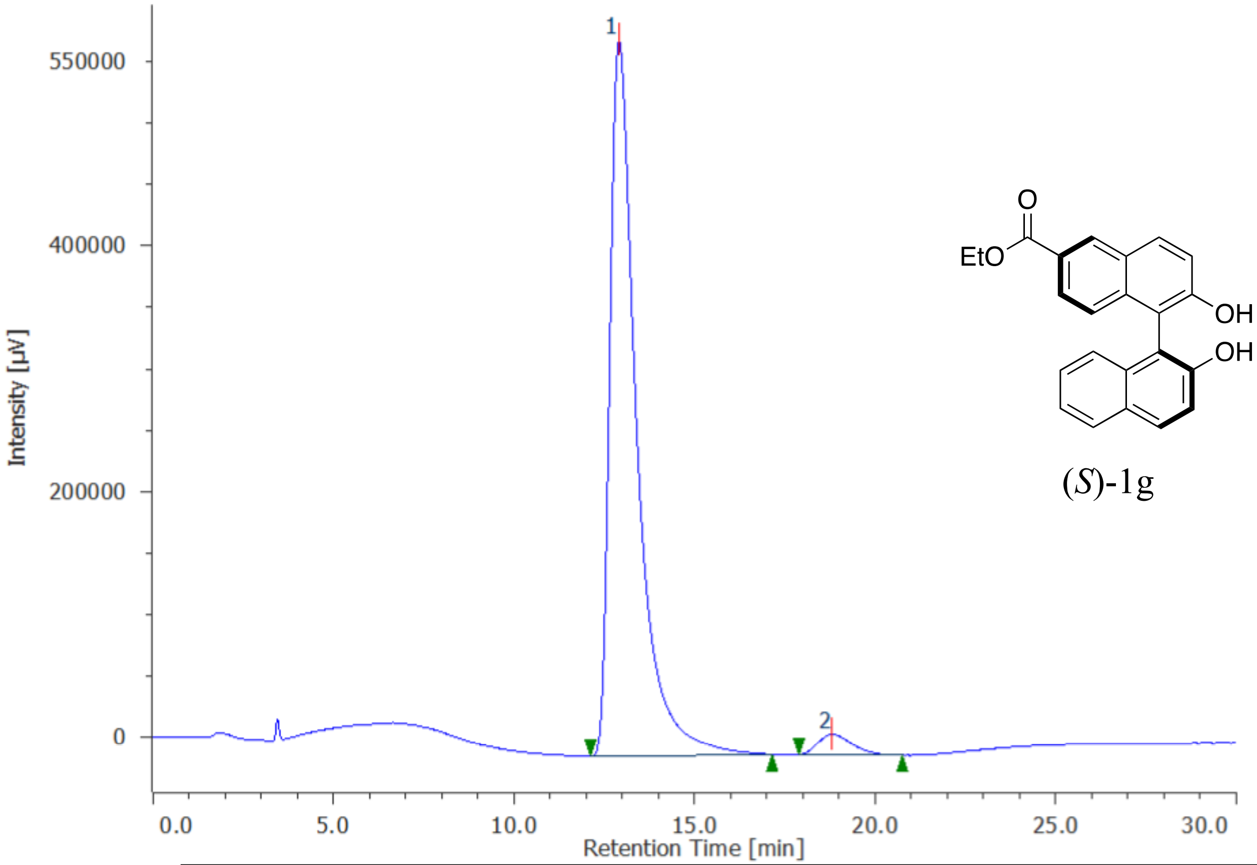

(*S*)-1g

#	ピーク名	CH	tR [min]	面積 [μV·sec]	高さ [μV]	面積%	高さ%	定量値	NTP	分離度	シンメトリー係数	警告
1	Unknown	9	12.913	30325295	581986	96.485	97.178	N/A	1739	3.989	1.977	
2	Unknown	9	18.810	1104769	16898	3.515	2.822	N/A	1902	N/A	1.369	

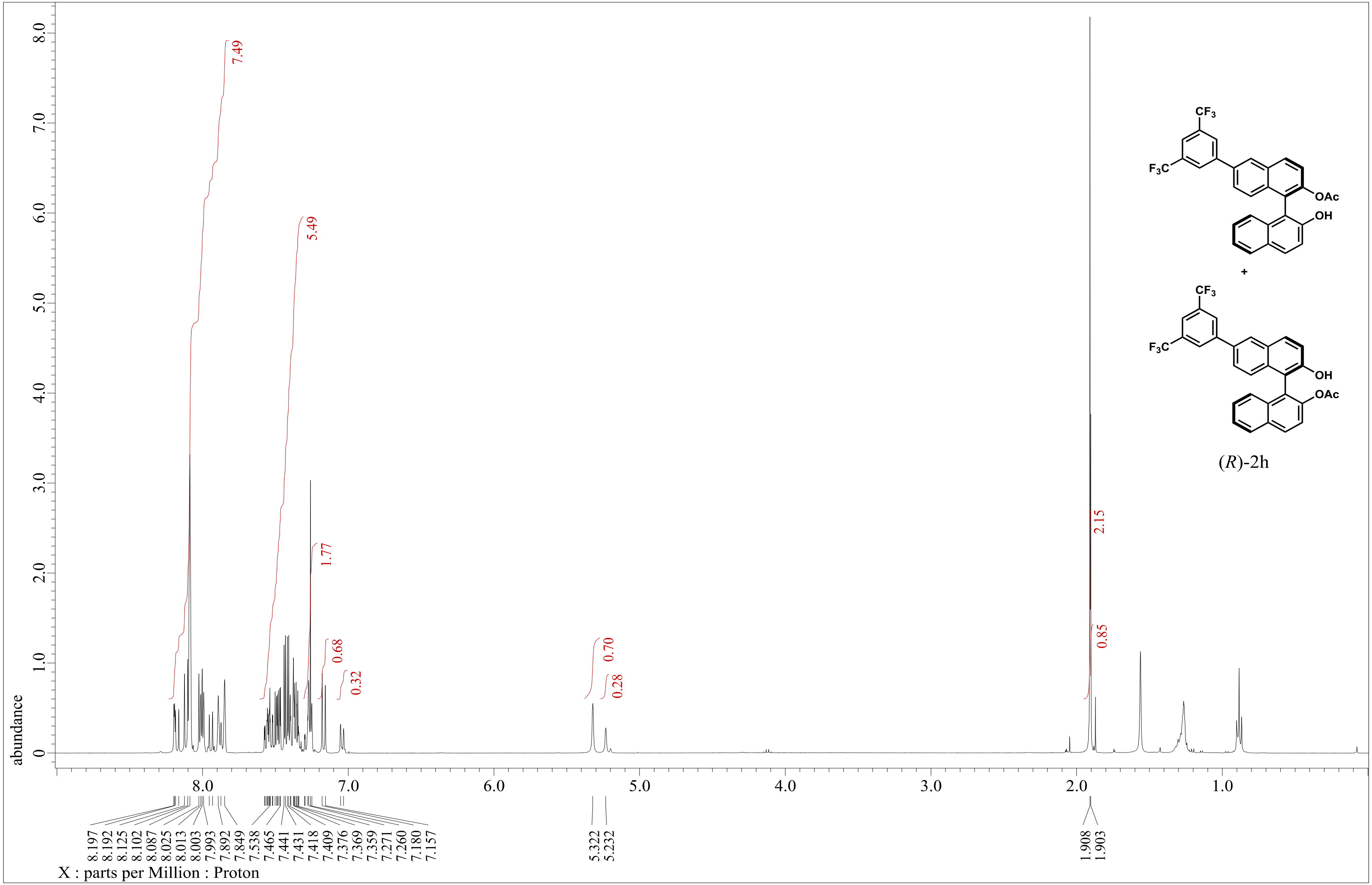

^{1}H NMR spectra (400 MHz, CDCl3) of (*R*)-**2h**

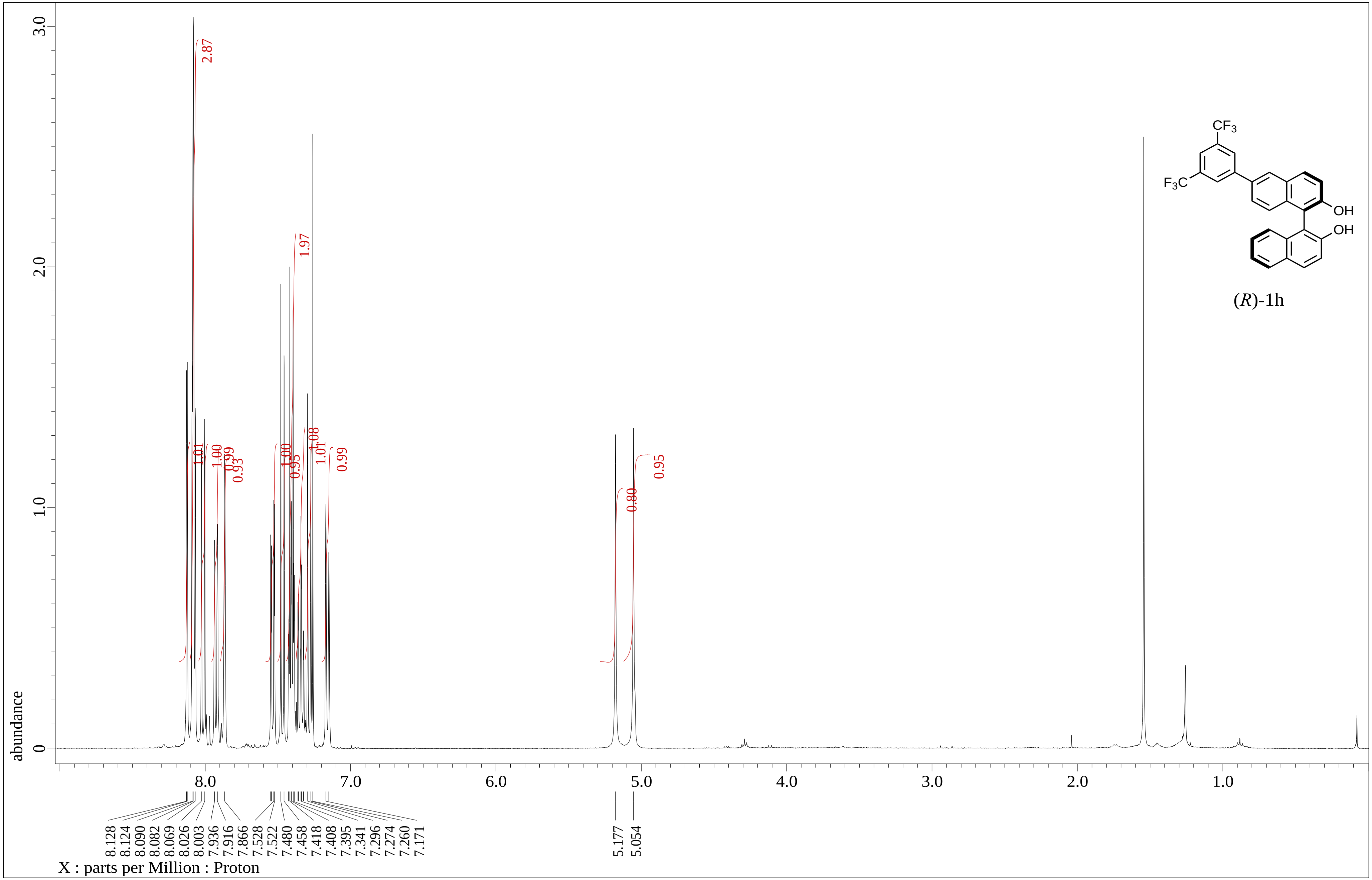

^{1}H NMR spectrum (400 MHz, $CDCl_3$) of (*R*)-**1h**

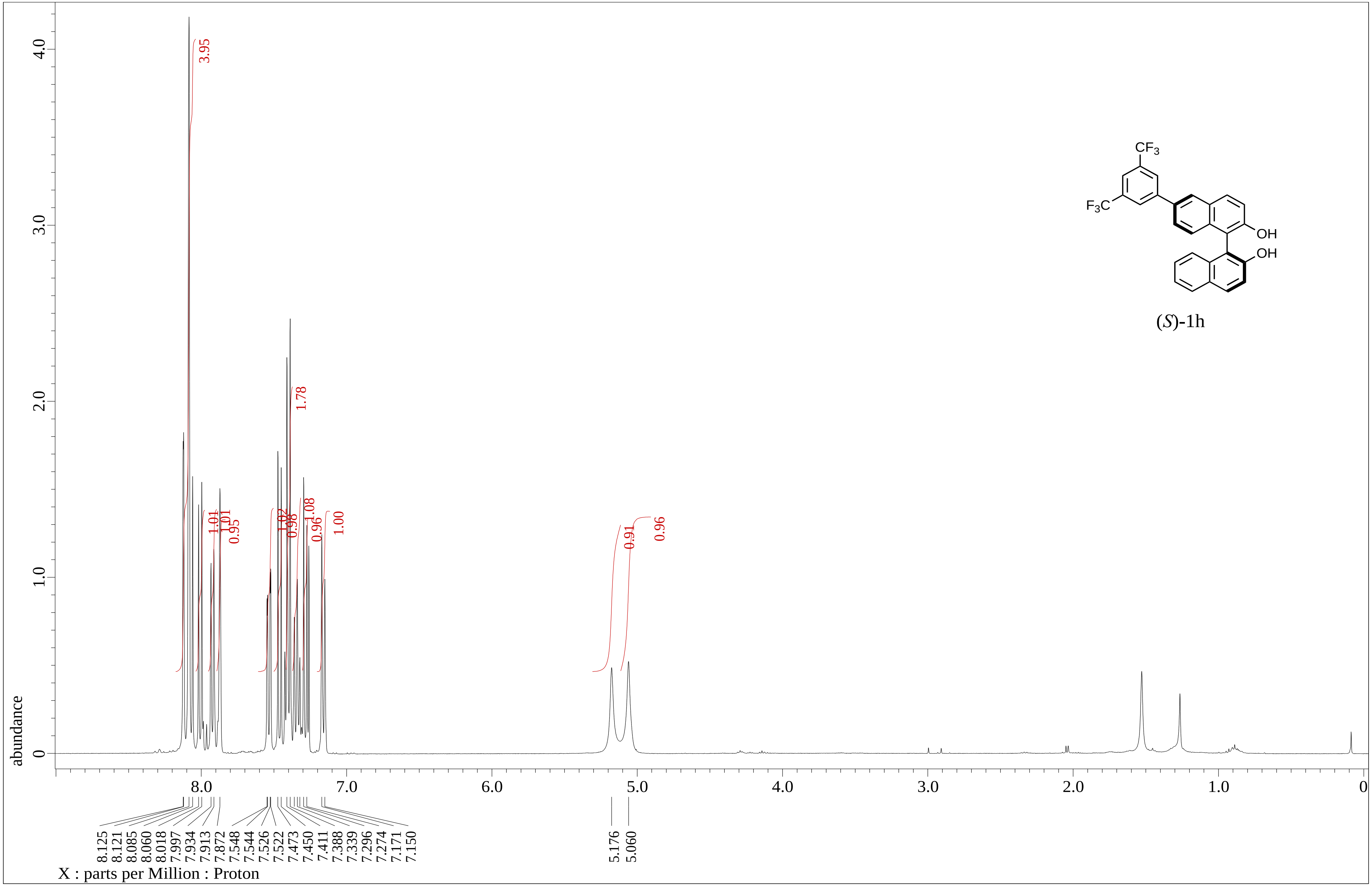

^{1}H NMR spectrum (400 MHz, $CDCl_3$) of (*S*)-**1**

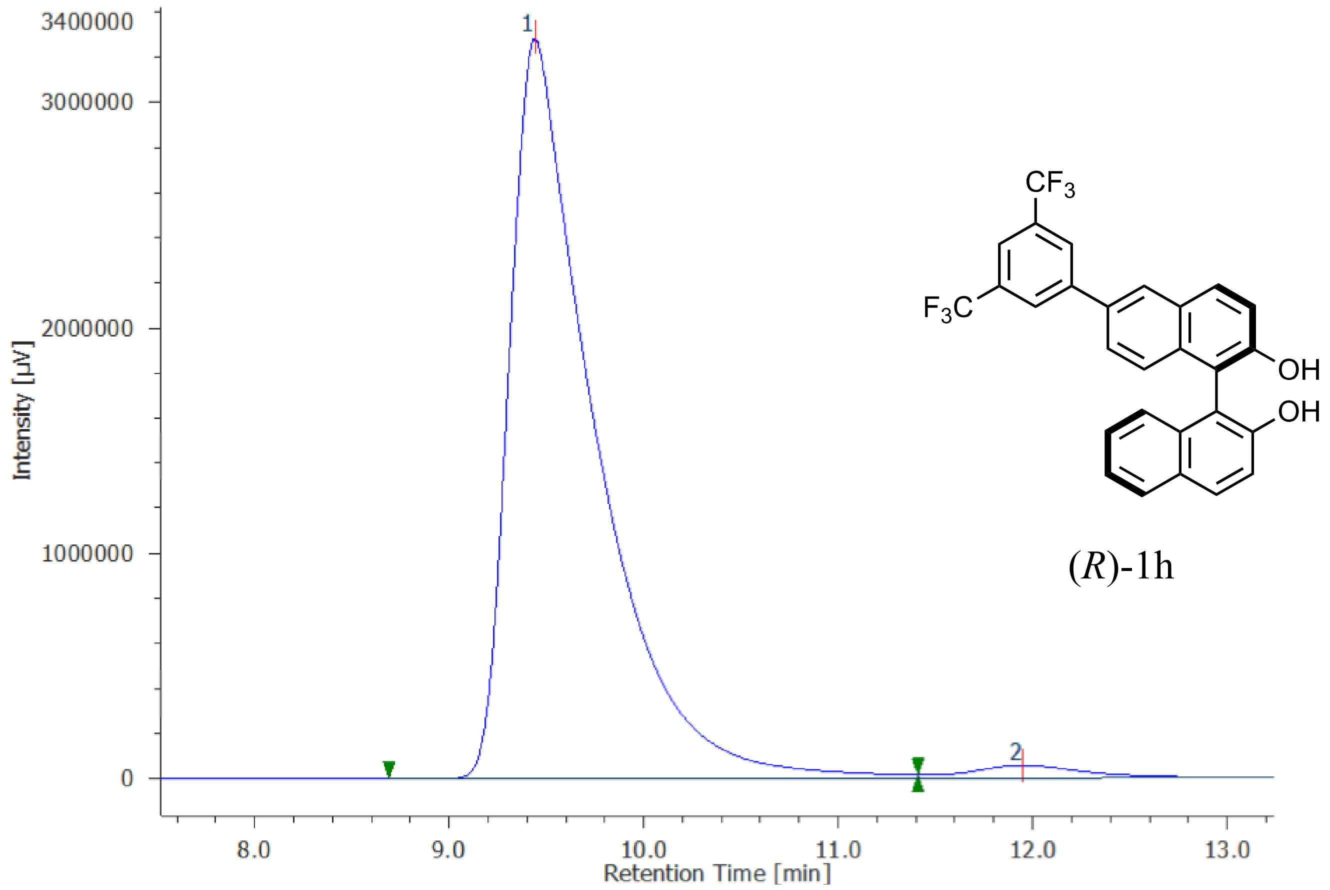

#	ピーク名	CH	tR [min]	面積 [μV·sec]	高さ [μV]	面積%	高さ%	定量値	NTP	分離度	シンメトリー係数	警告
1	Unknown	9	9.443	100343899	3284082	97.730	98.338	N/A	2578	2.894	2.121	
2	Unknown	9	11.950	2331205	55487	2.270	1.662	N/A	2317	N/A	N/A	

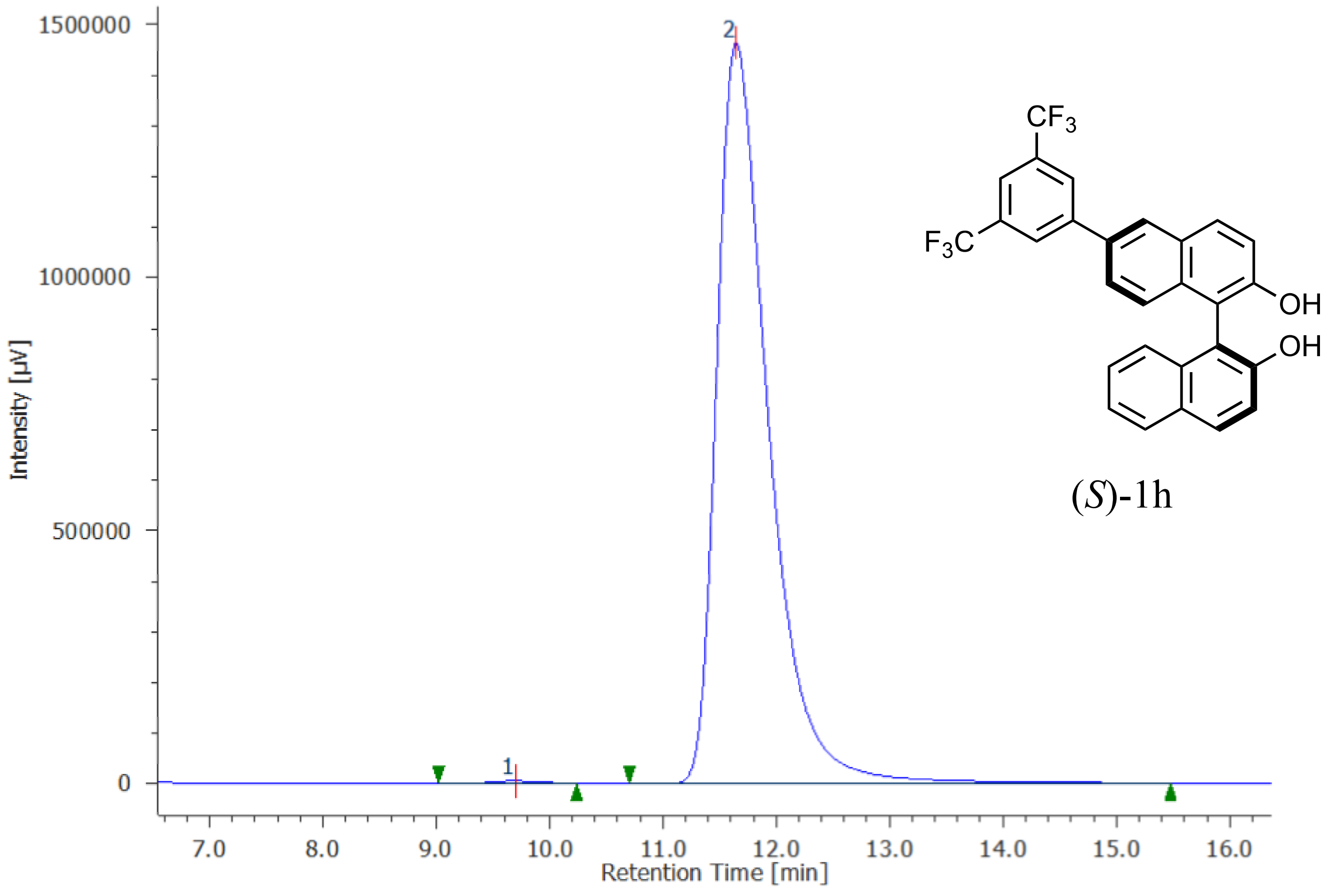

#	ピーク名	CH	tR [min]	面積 [μV·sec]	高さ [μV]	面積%	高さ%	定量値	NTP	分離度	シンメトリー係数	警告
1	Unknown	10	9.700	126056	4489	0.271	0.306	N/A	2501	2.491	1.133	
2	Unknown	10	11.647	46324946	1462624	99.729	99.694	N/A	3467	N/A	1.589	

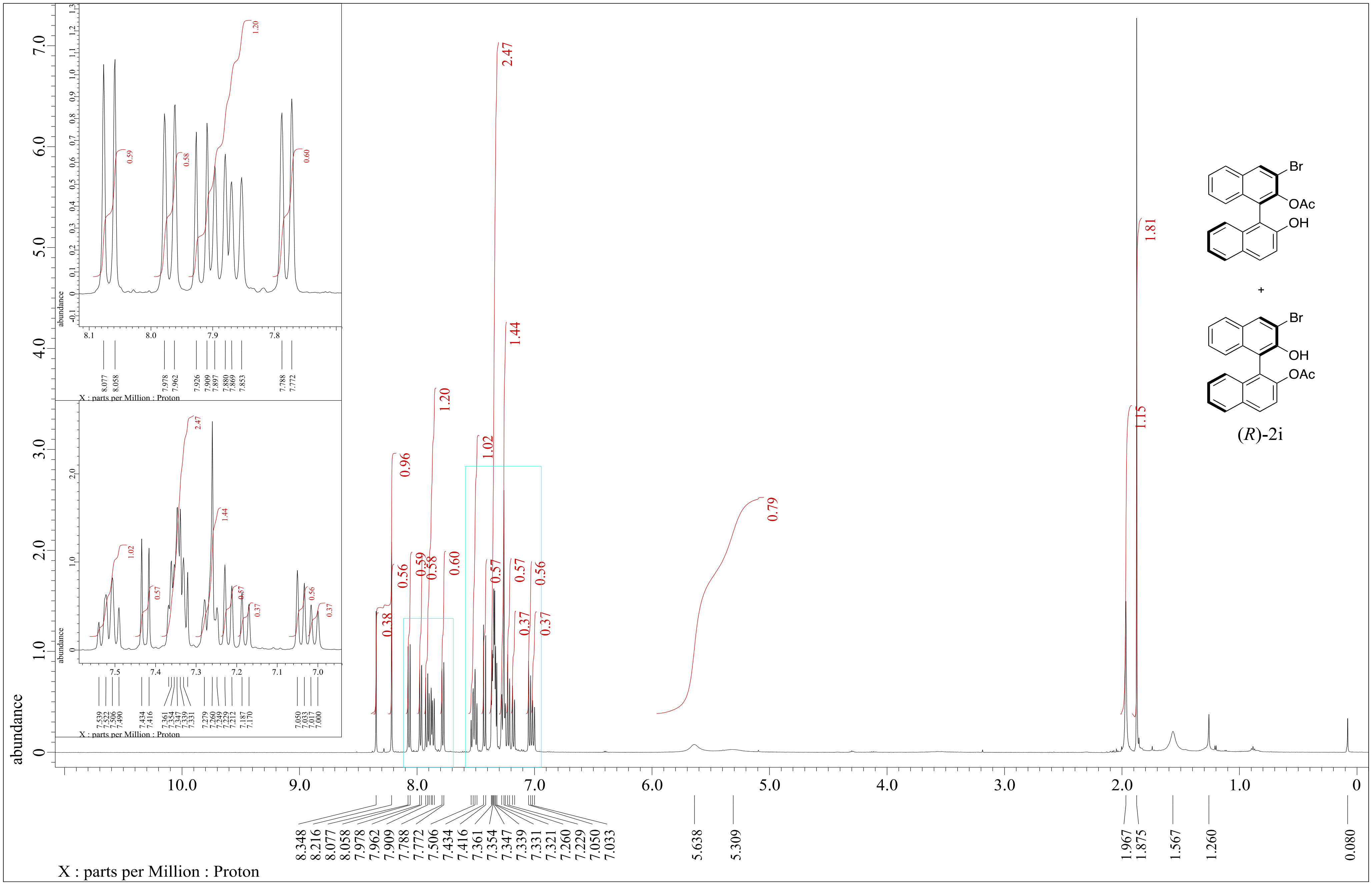

^{1}H NMR spectrum (500 MHz, $CDCl_3$) of (*R*)-**2i**

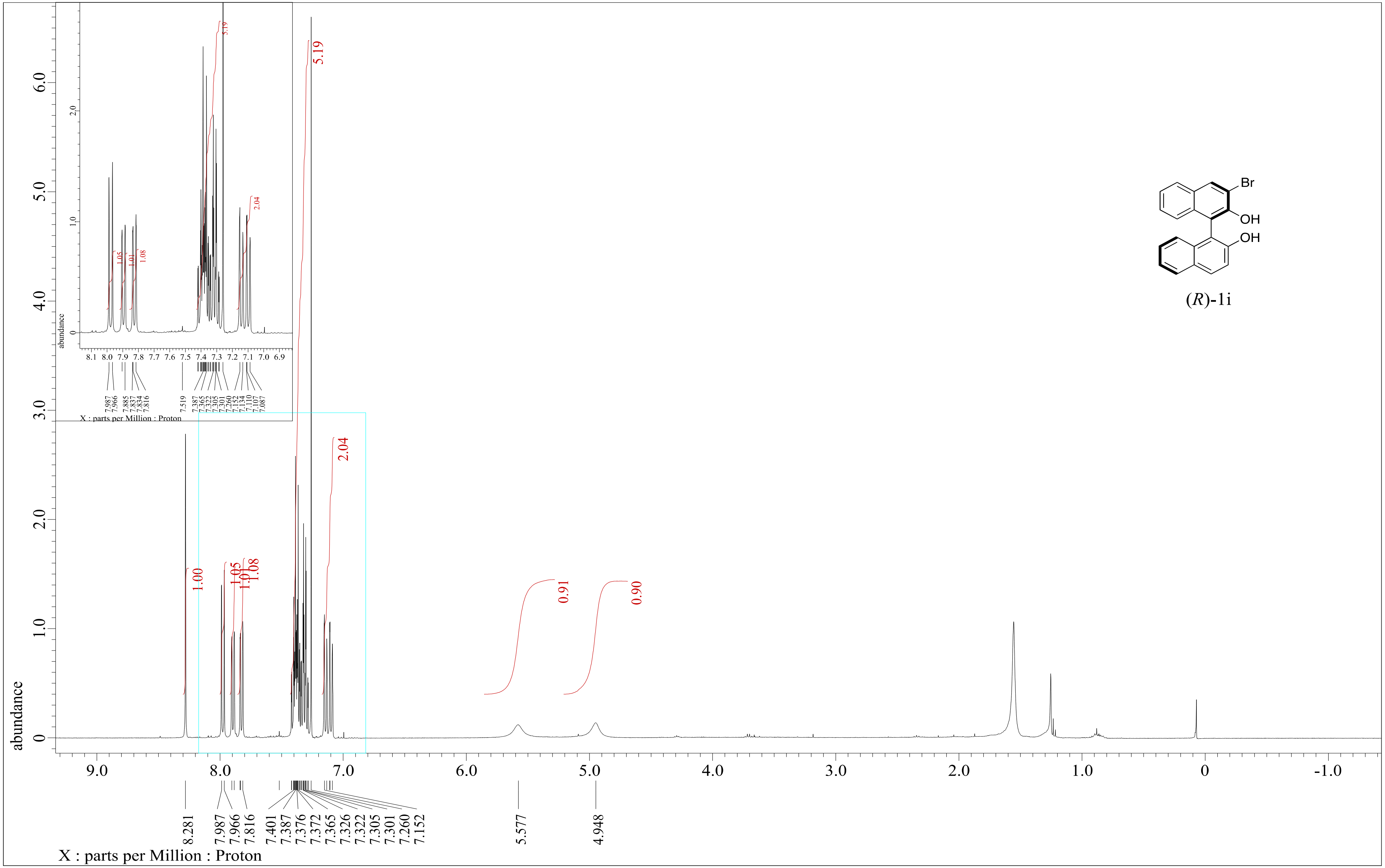

^{1}H NMR spectrum (400 MHz, $CDCl_3$) of (*R*)-**1i**

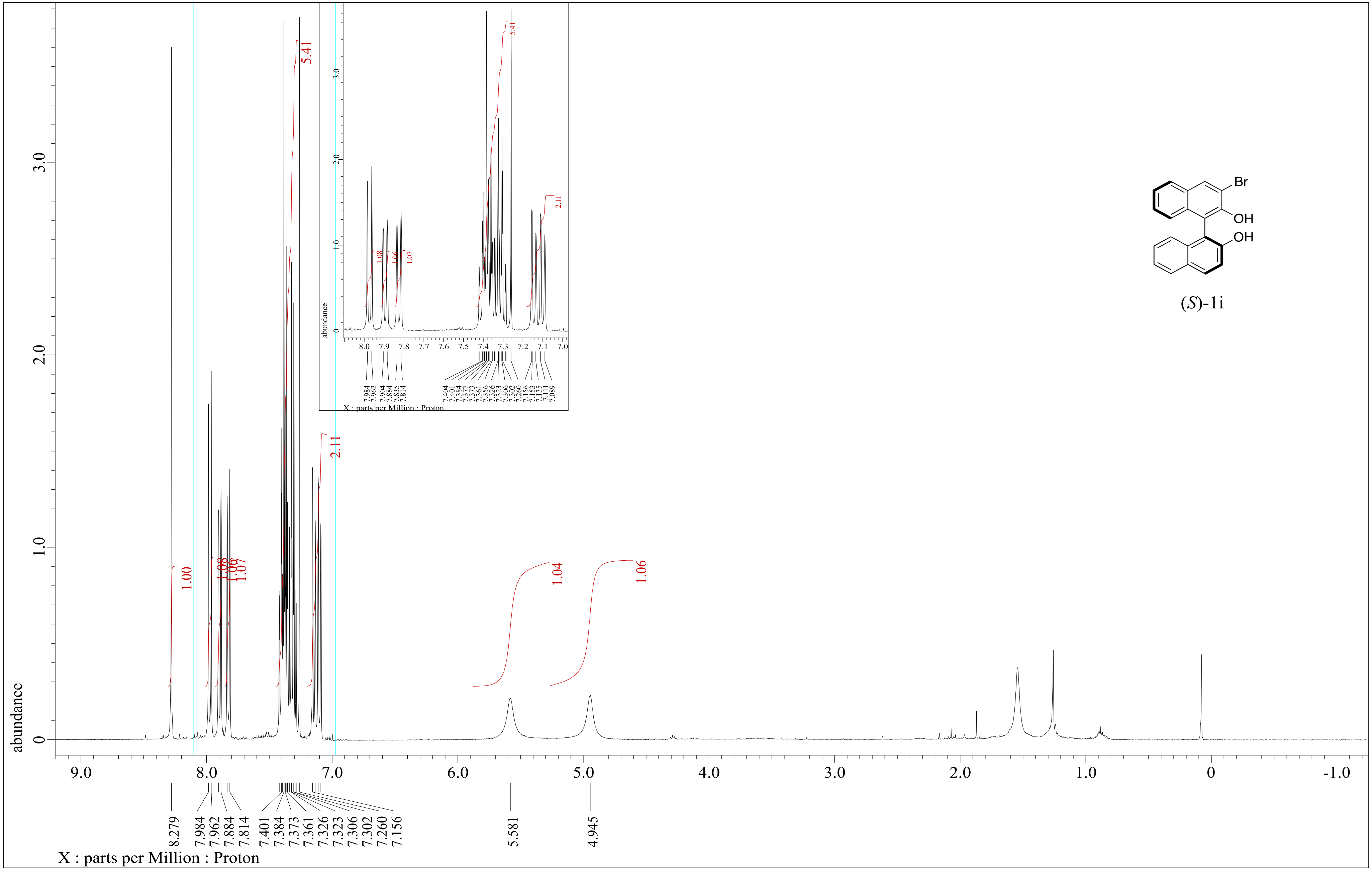

^{1}H NMR spectrum (400 MHz, $CDCl_3$) of (*S*)-**1i**

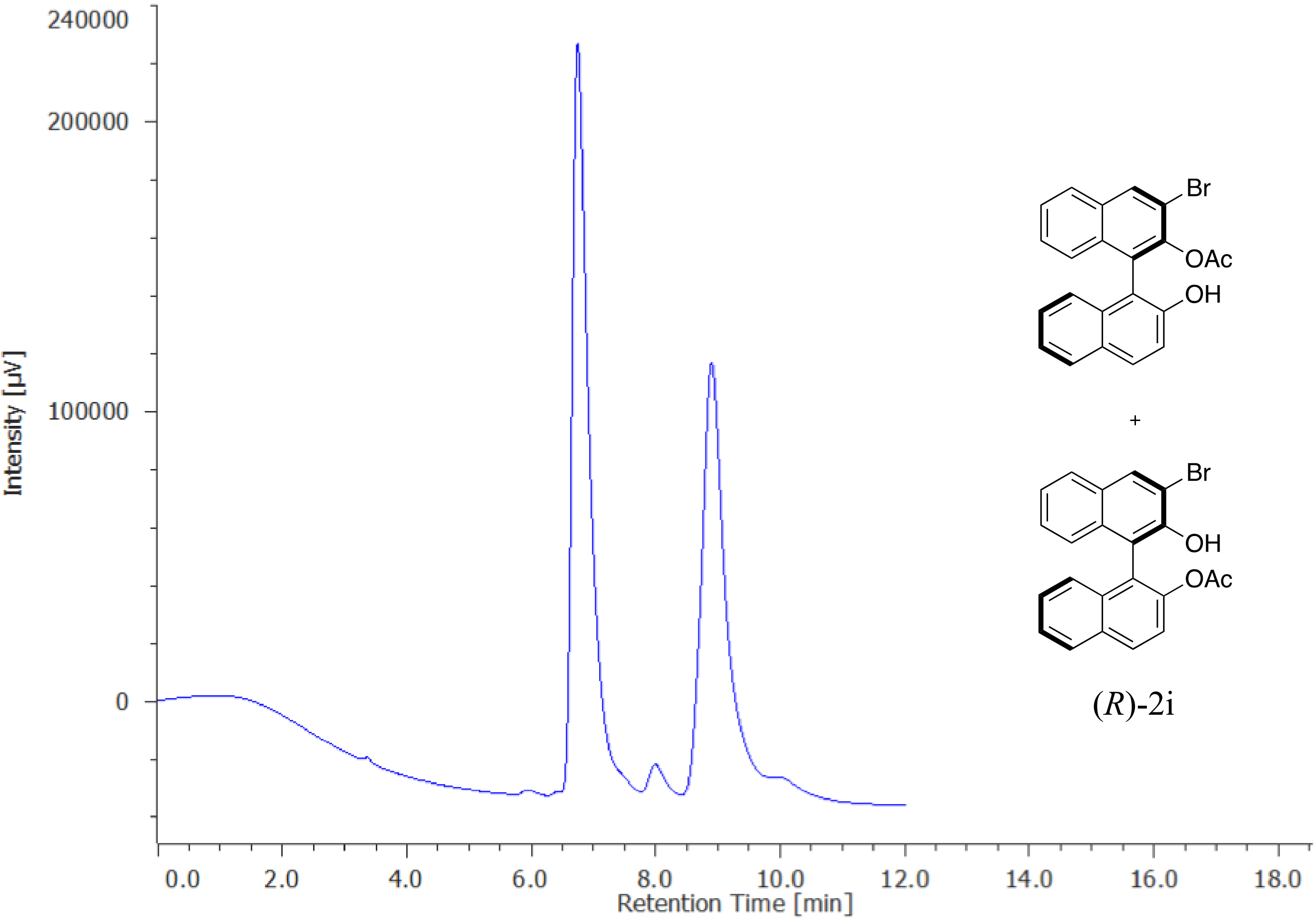
240000
200000
100000
0
Intensity [µV]
0.0
2.0
4.0
6.0
8.0
10.0
12.0
14.0
16.0
18.0
Retention Time [min]
Br
OAc
OH
+
Br
OH
OAc
(R)-2i

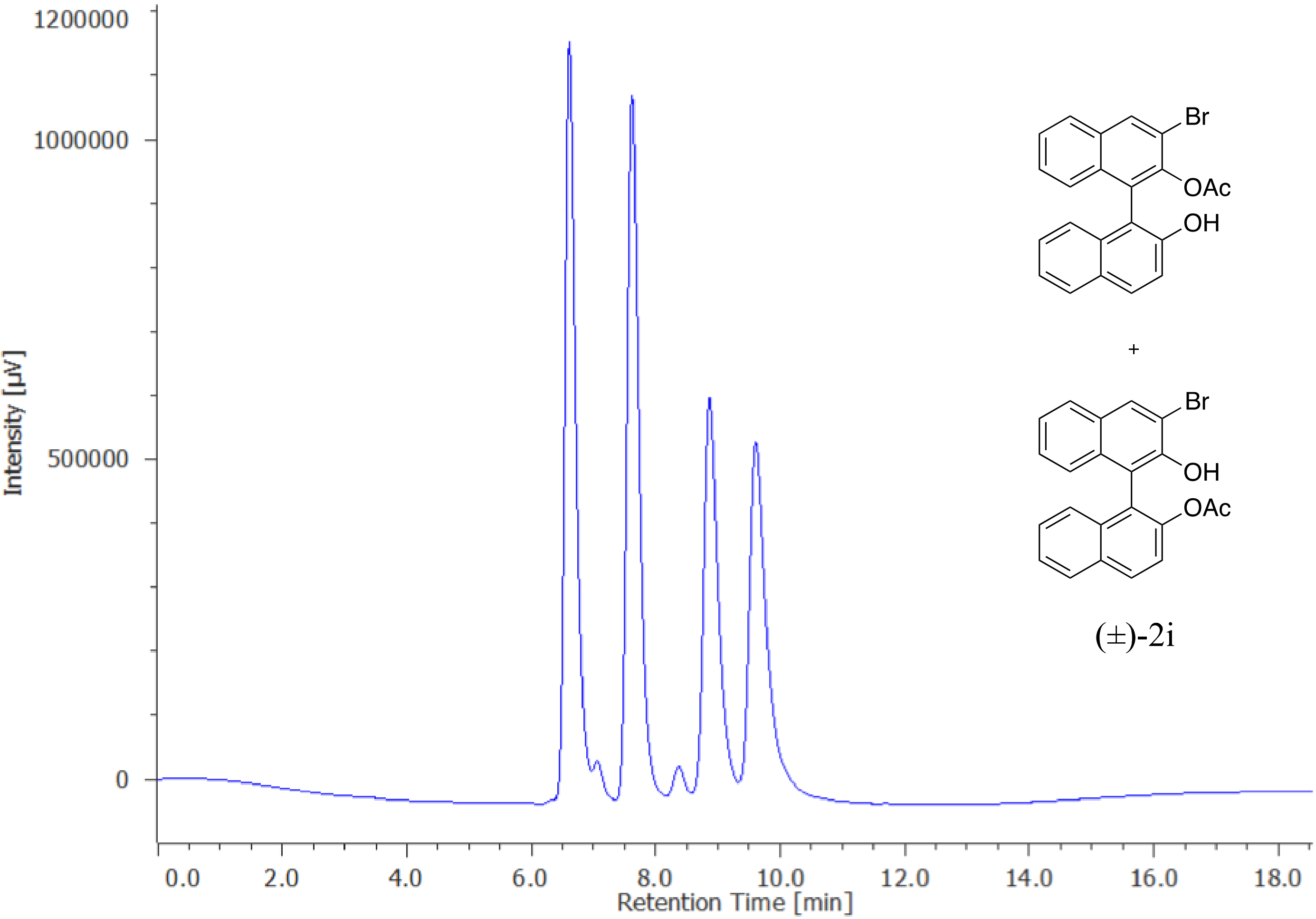
1200000
1000000
500000
0
Intensity [µV]
0.0
2.0
4.0
6.0
8.0
10.0
12.0
14.0
16.0
18.0
Retention Time [min]
Br
OAc
OH
+
Br
OH
OAc
(±)-2i

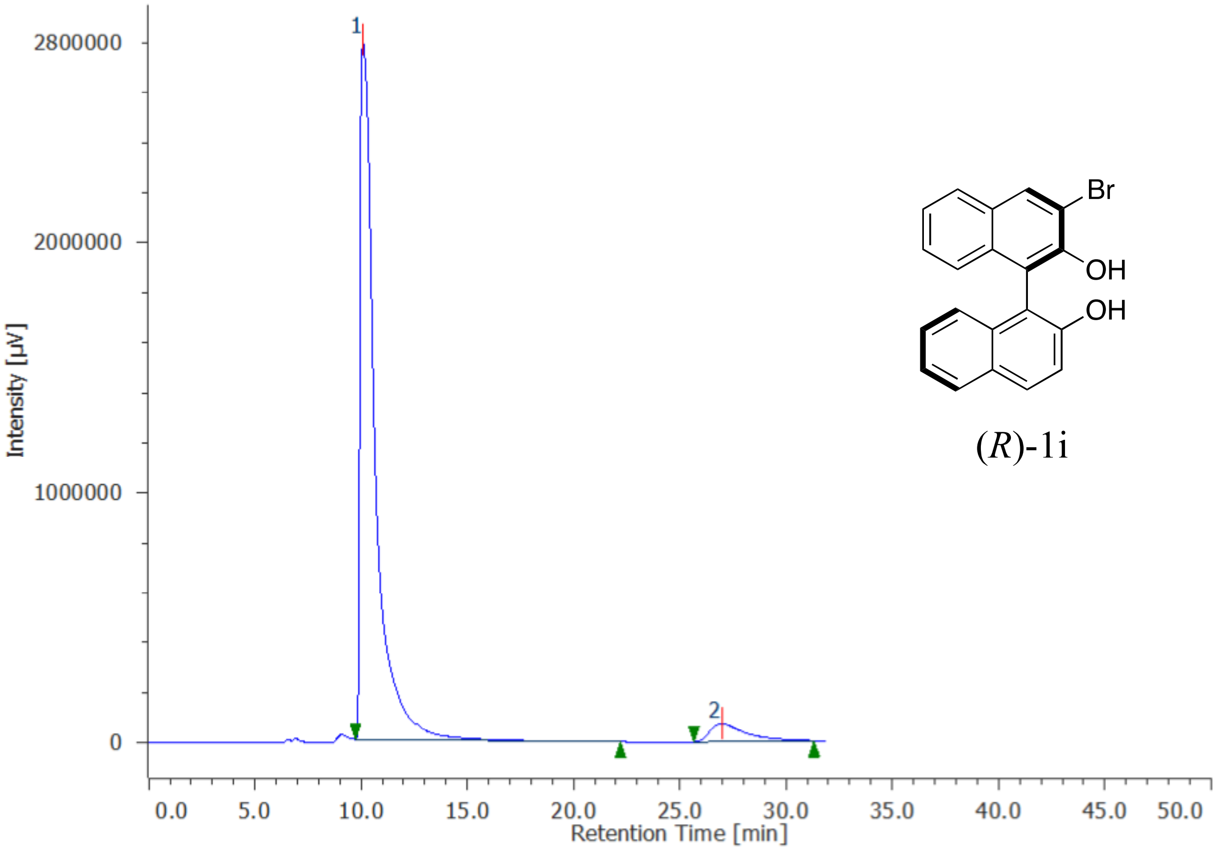

#	ピーク名	CH	tR [min]	面積 [μV·sec]	高さ [μV]	面積%	高さ%	定量値	NTP	分離度	シンメトリー係数	警告
1	Unknown	6	10.087	121213244	2991984	95.605	98.280	N/A	2146	9.502	3.979	
2	Unknown	6	26.980	5571902	52363	4.395	1.720	N/A	1605	N/A	1.870	

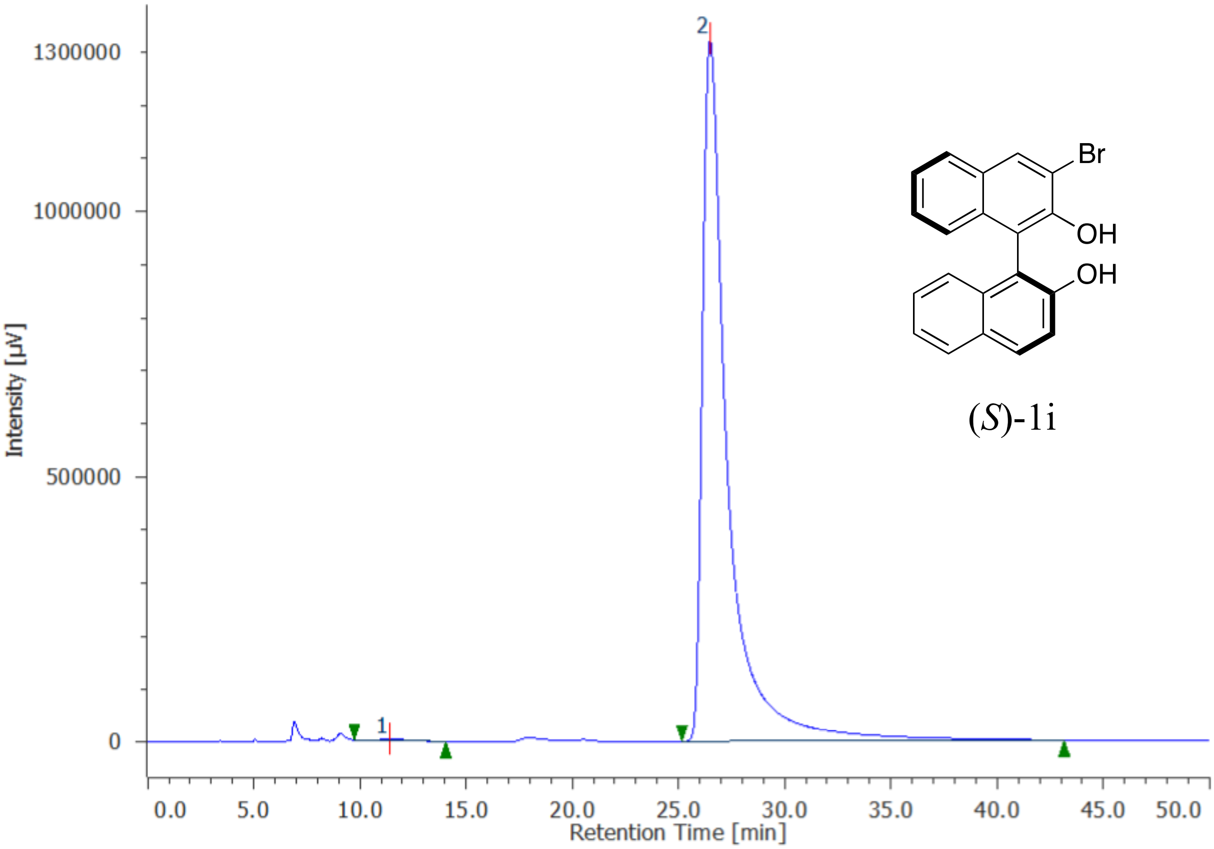

#	ピーク名	CH	tR [min]	面積 [μV·sec]	高さ [μV]	面積%	高さ%	定量値	NTP	分離度	シンメトリー係数	警告
1	Unknown	9	11.373	235037	2783	0.213	0.210	N/A	415	7.566	1.221	
2	Unknown	9	26.487	109925291	1322223	99.787	99.790	N/A	3576	N/A	2.508	

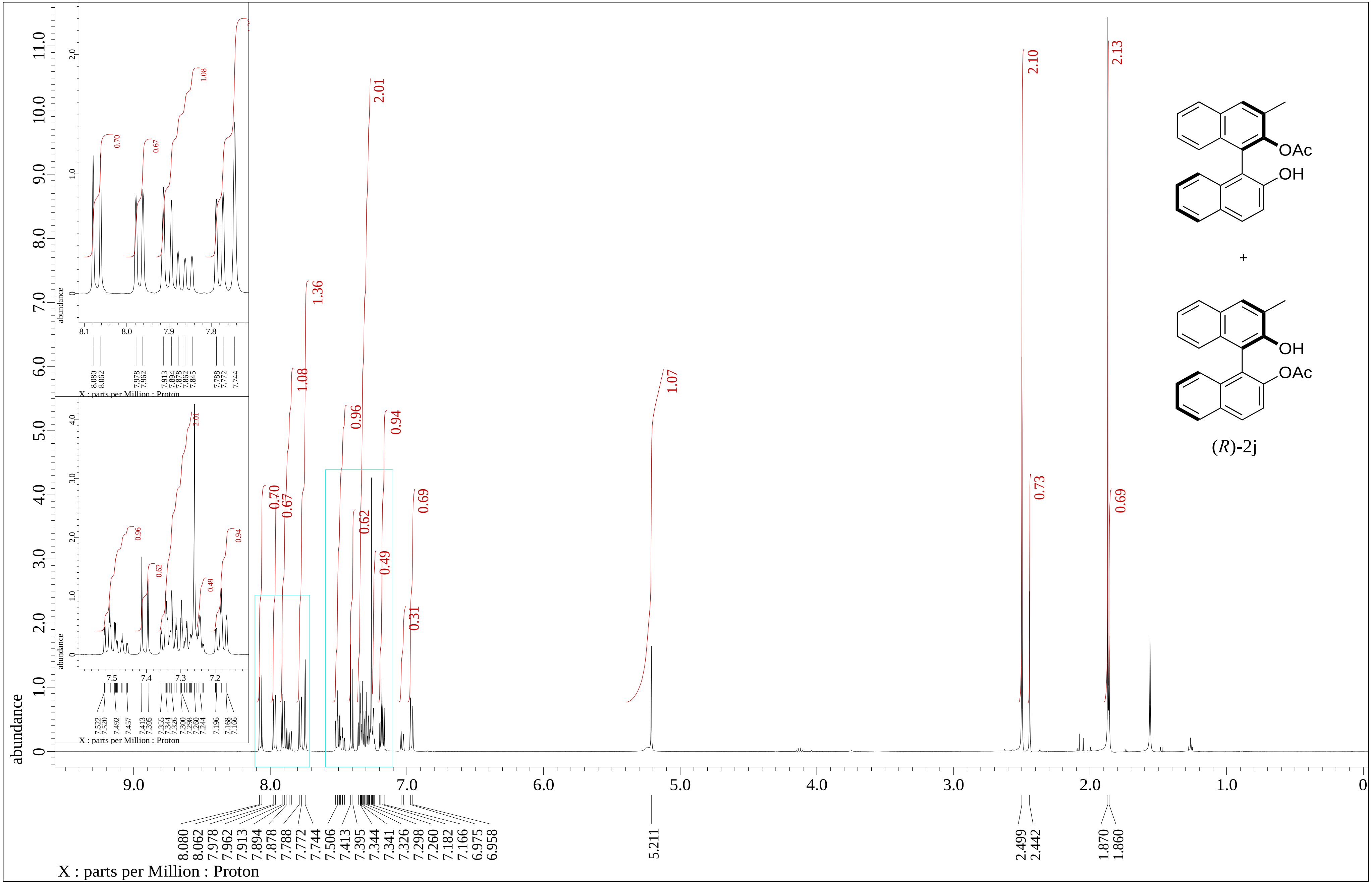

^{1}H NMR spectrum (500 MHz, CDCl3) of (*R*)-**2j**

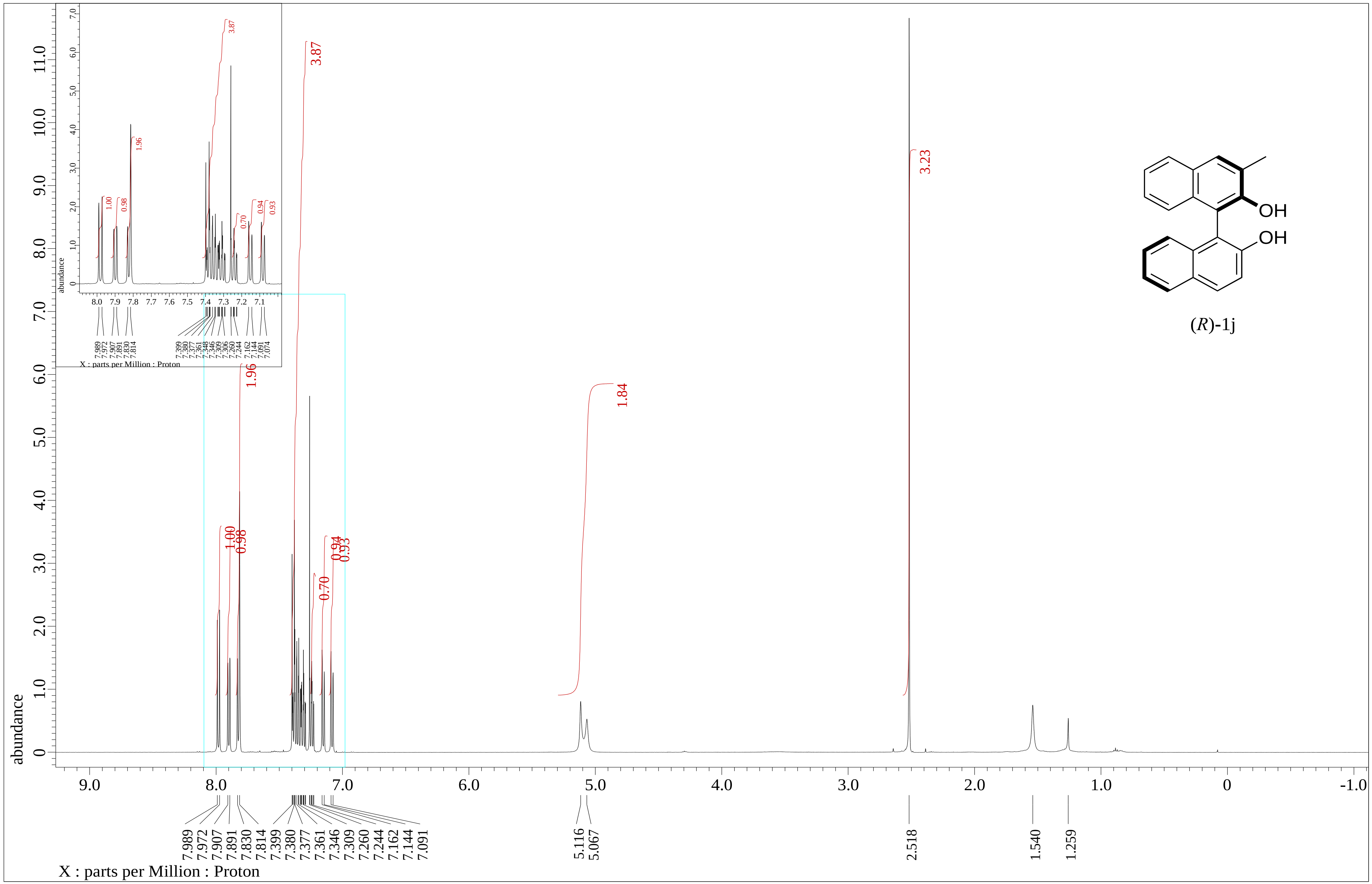

^{1}H NMR spectrum (500 MHz, $CDCl_3$) of (*R*)-**1j**

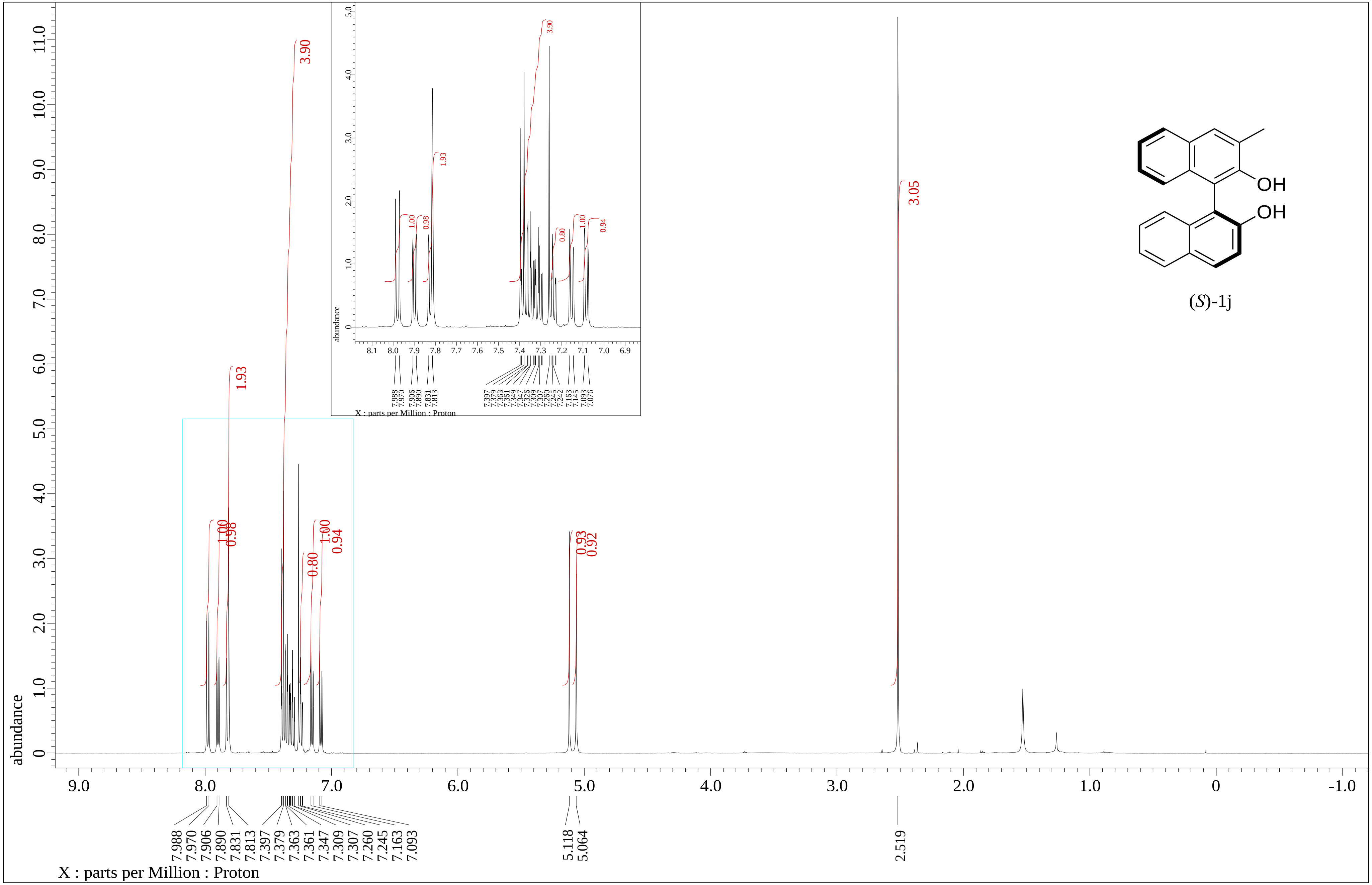

^{1}H NMR spectrum (500 MHz, $CDCl_3$) of (*S*)-**1j**

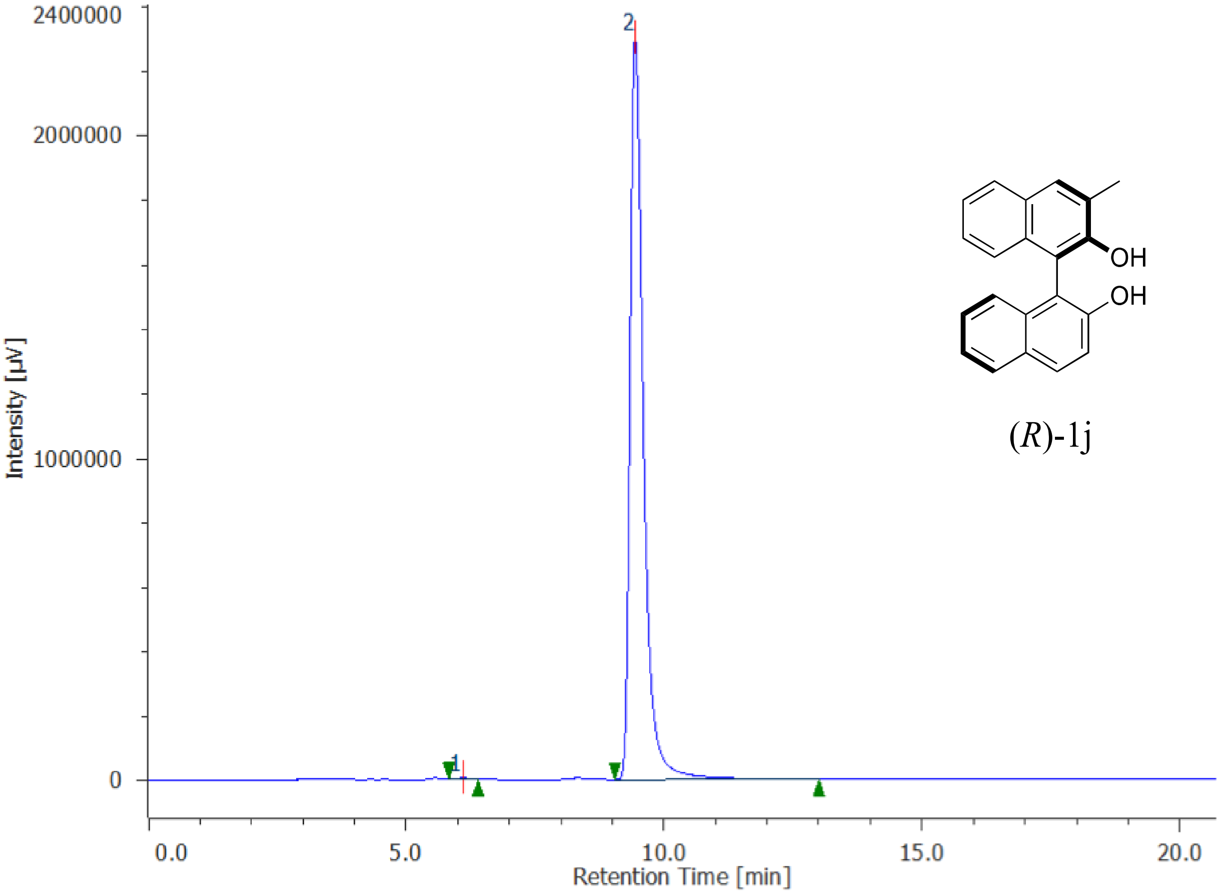

(*R*)-1j

#	ピーク名	CH	tR [min]	面積 [µV·sec]	高さ [µV]	面積%	高さ%	定量値	NTP	分離度	シンメトリー係数	警告
1	Unknown	9	5.957	12489285	997407	95.780	96.955	N/A	6626	9.067	1.754	
2	Unknown	9	9.297	550302	31322	4.220	3.045	N/A	6952	N/A	1.514	

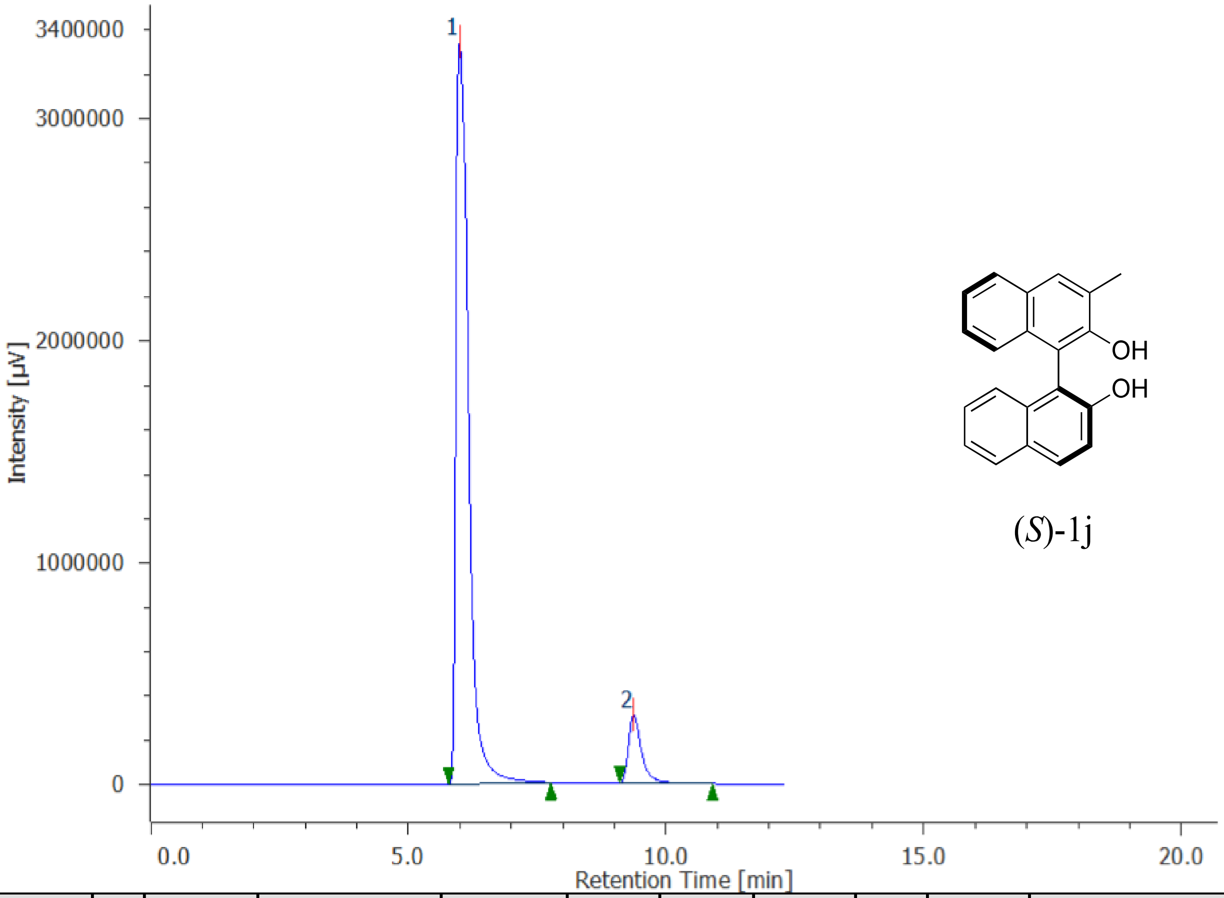

(*S*)-1j

#	ピーク名	CH	tR [min]	面積 [µV·sec]	高さ [µV]	面積%	高さ%	定量値	NTP	分離度	シンメトリー係数	警告
1	Unknown	9	6.093	94943	7427	0.203	0.316	N/A	5401	8.181	1.490	
2	Unknown	9	9.437	46635200	2345576	99.797	99.684	N/A	5985	N/A	1.652	

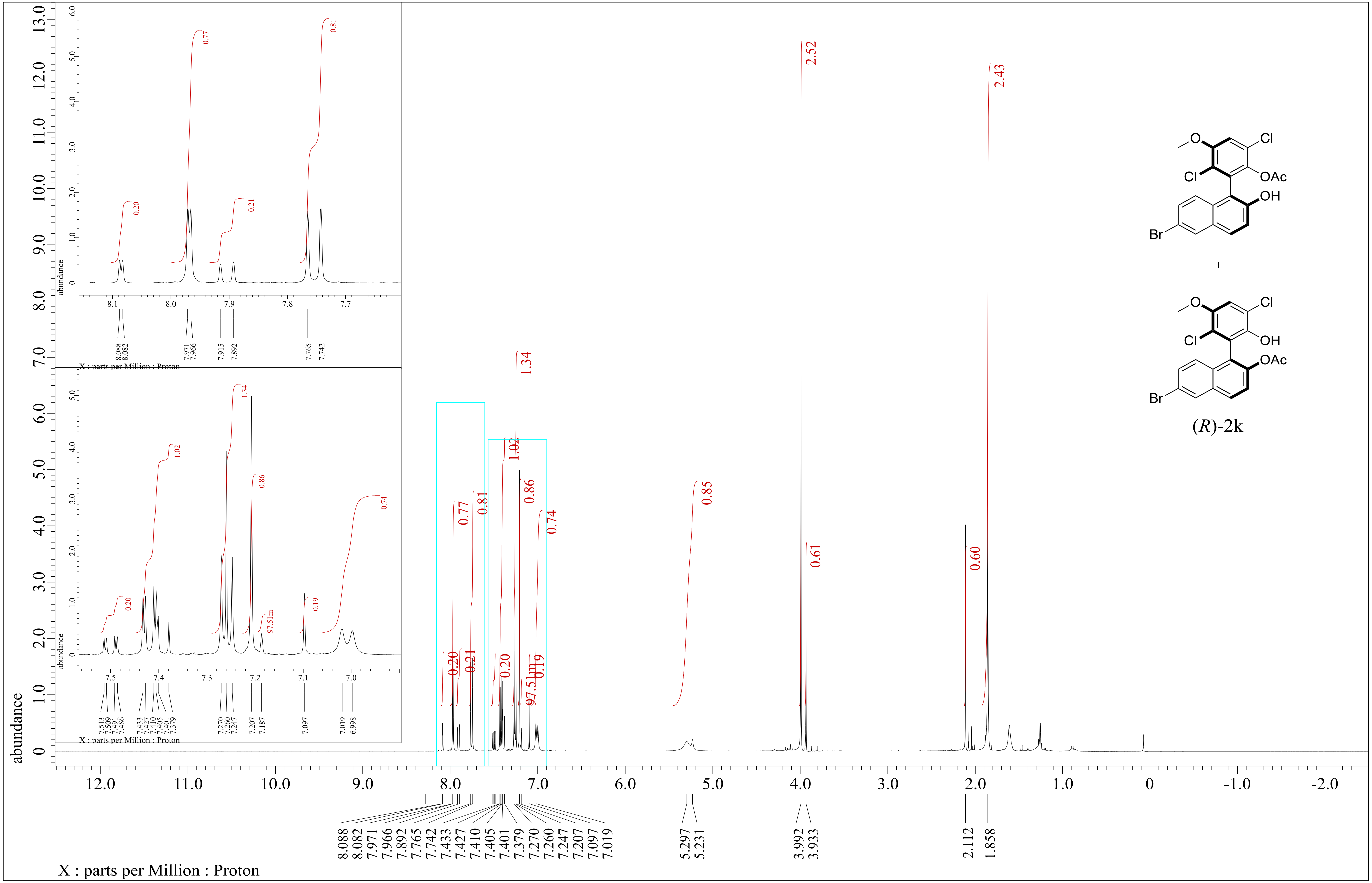

^{1}H NMR spectrum (500 MHz, $CDCl_3$) of (*R*)-**1k**

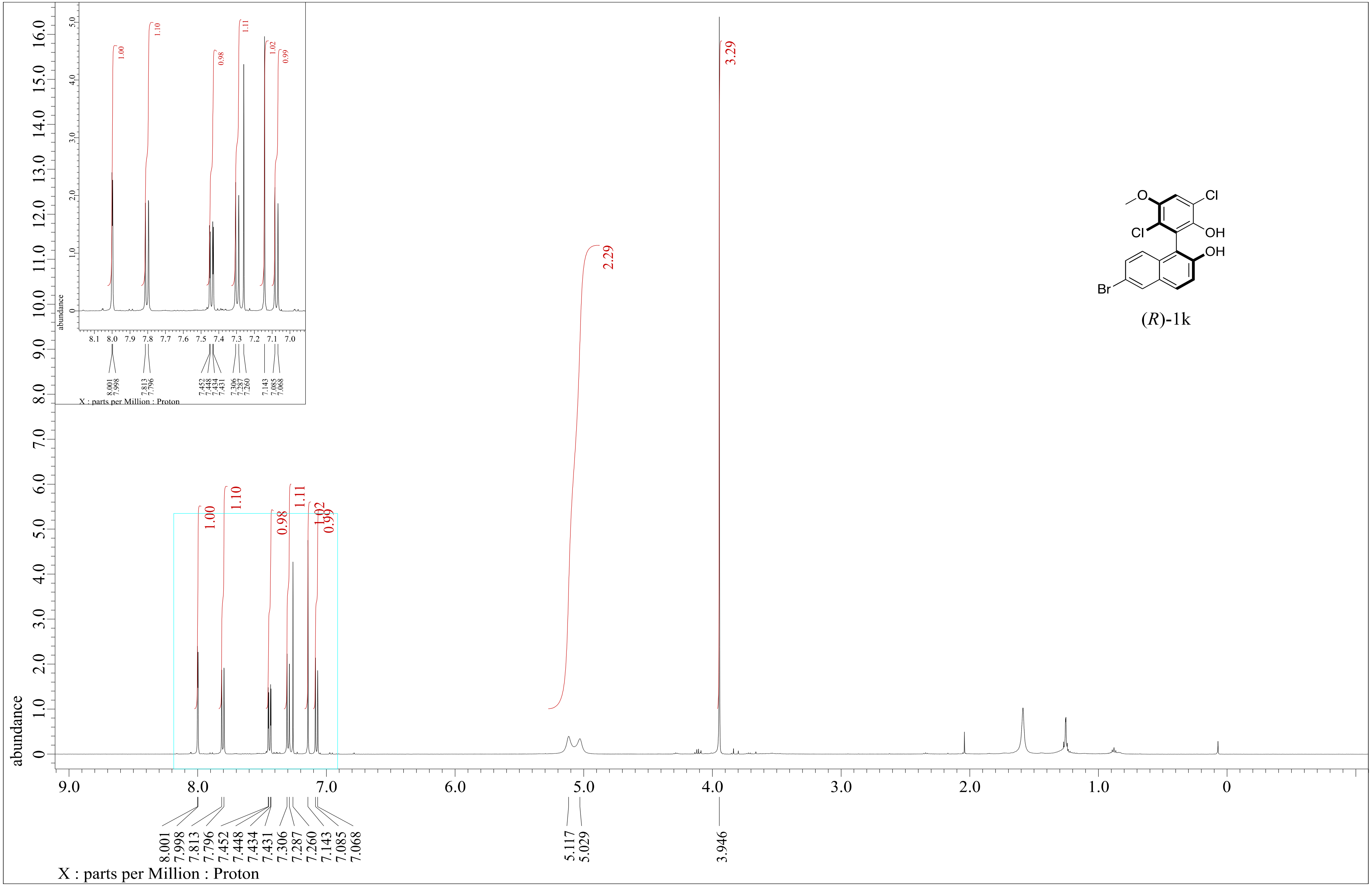

^{1}H NMR spectrum (500 MHz, $CDCl_3$) of (*R*)-**1k**

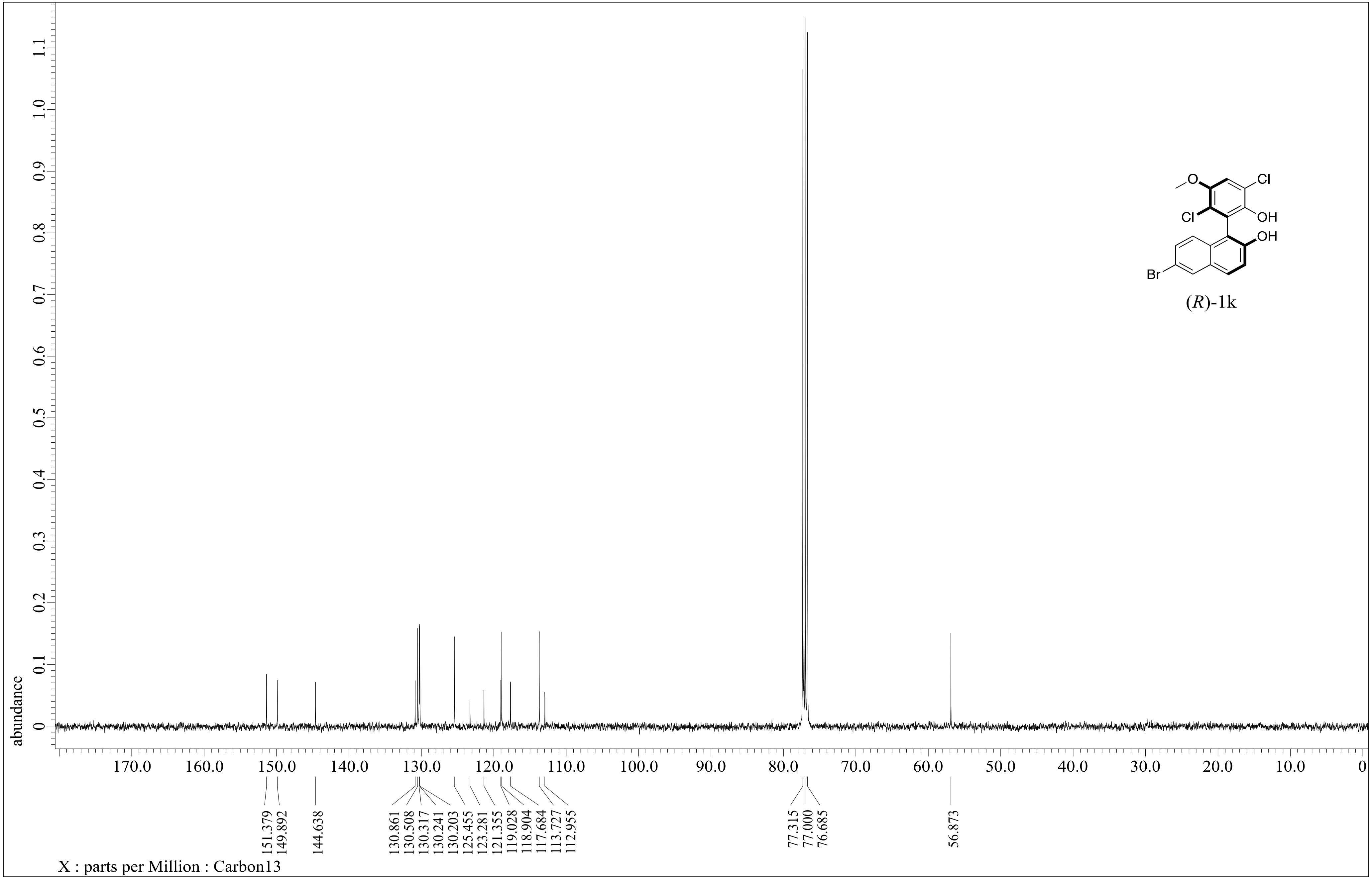

^{13}C NMR spectrum (100 MHz, $CDCl_3$) of (*R*)-**1k**

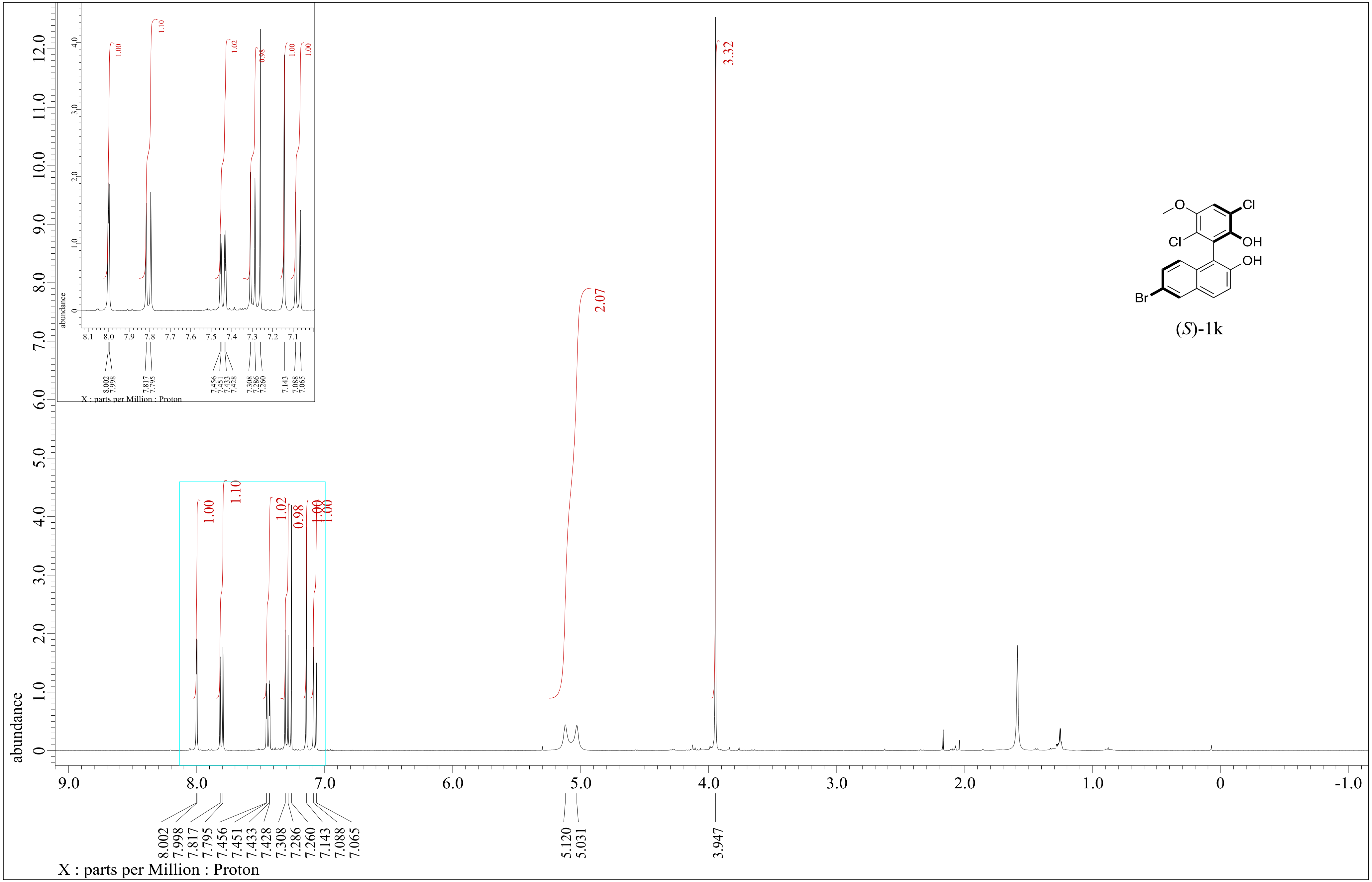

^{1}H NMR spectrum (400 MHz, $CDCl_3$) of (*S*)-**1k**

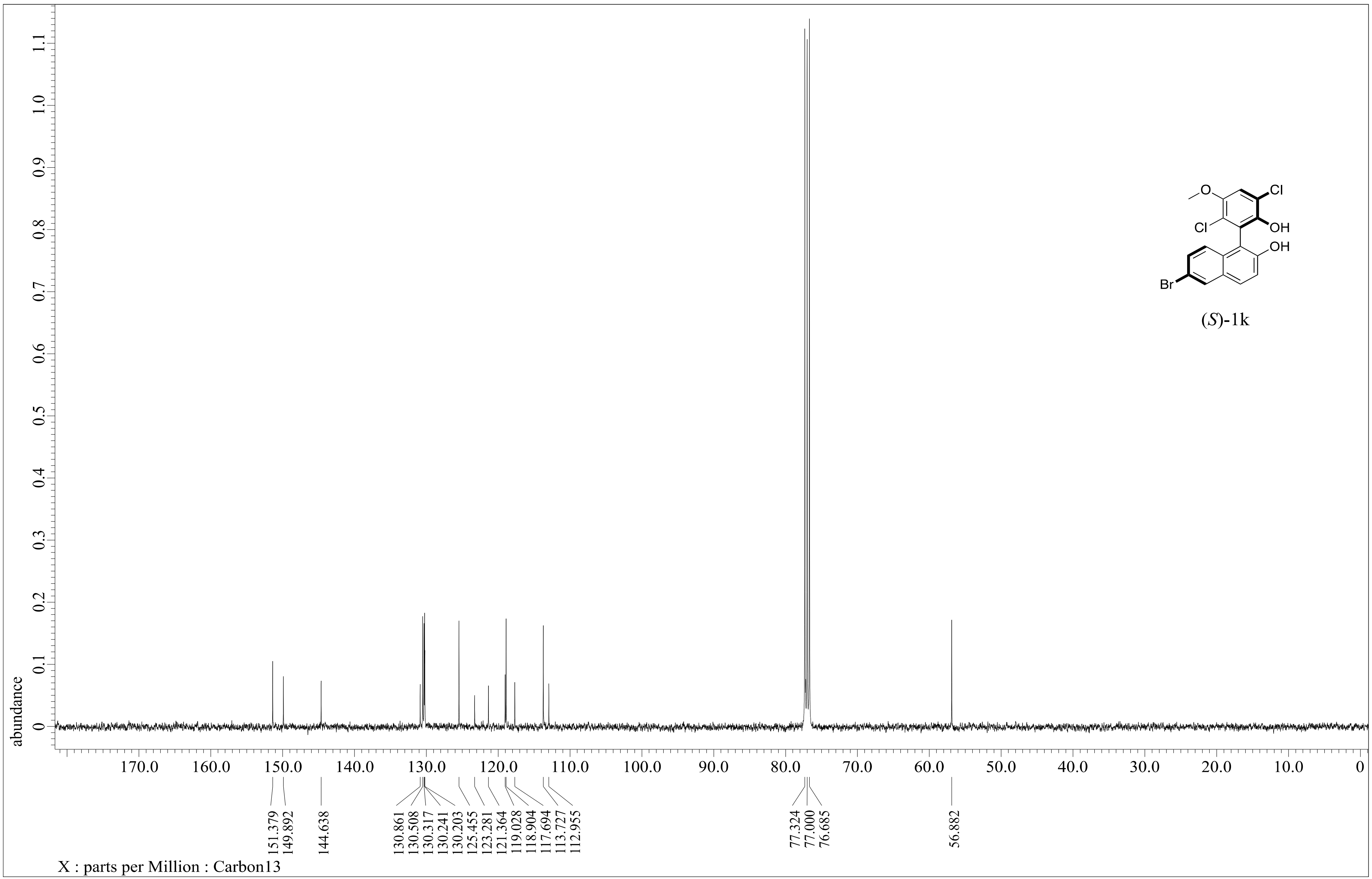

^{13}C NMR spectrum (100 MHz, $CDCl_3$) of (*S*)-**1k**

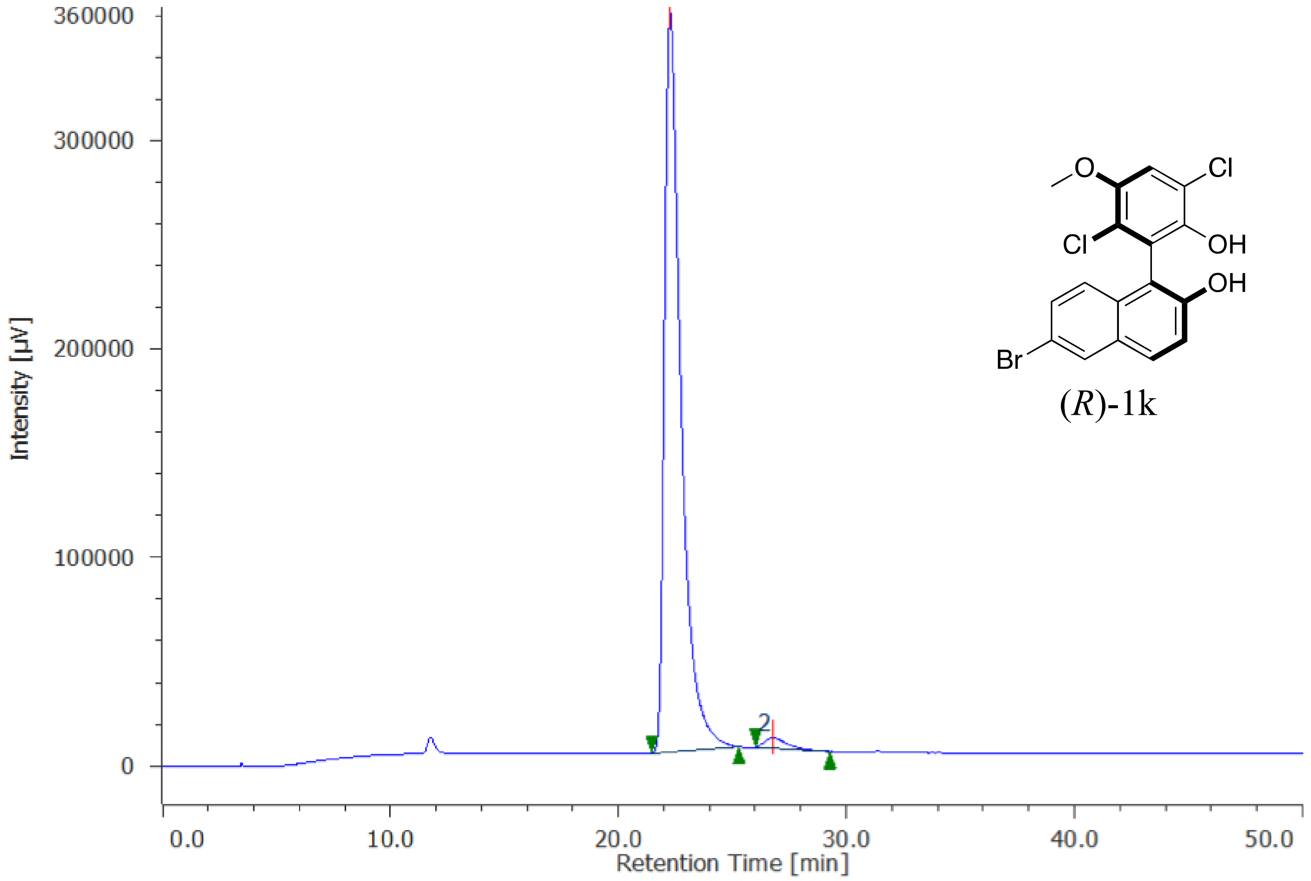

#	ピーク名	CH	tR [min]	面積 [µV·sec]	高さ [µV]	面積%	高さ%	定量値	NTP	分離度	シンメトリー係数	警告
1	Unknown	9	22.270	17900836	354826	98.155	98.644	N/A	5240	2.984	1.898	
2	Unknown	9	26.777	336494	4878	1.845	1.356	N/A	3549	N/A	1.640	

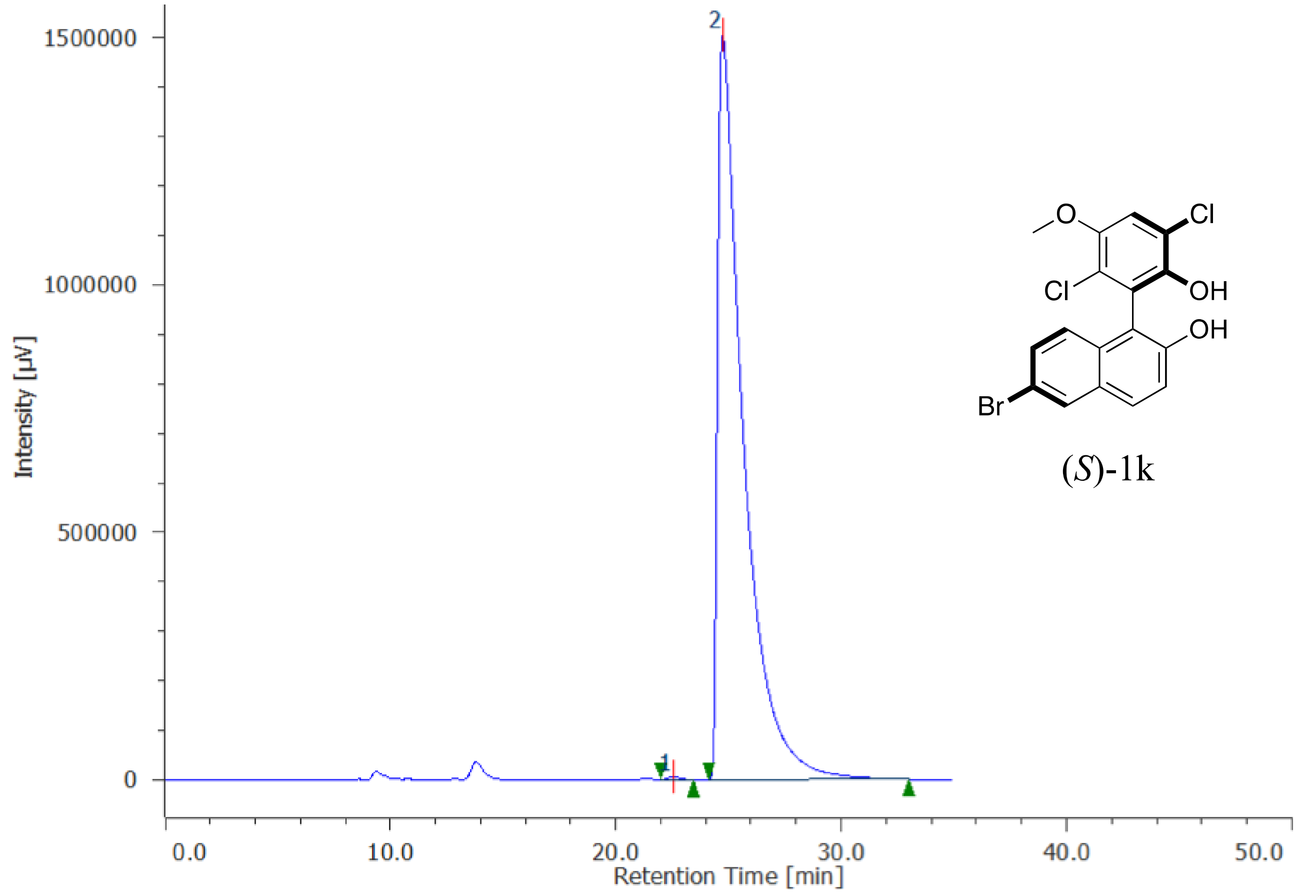

#	ピーク名	CH	tR [min]	面積 [µV·sec]	高さ [µV]	面積%	高さ%	定量値	NTP	分離度	シンメトリー係数	警告
1	Unknown	9	22.540	202886	5313	0.169	0.352	N/A	7690	1.528	1.284	
2	Unknown	9	24.743	120046402	1504739	99.831	99.648	N/A	2822	N/A	3.920	

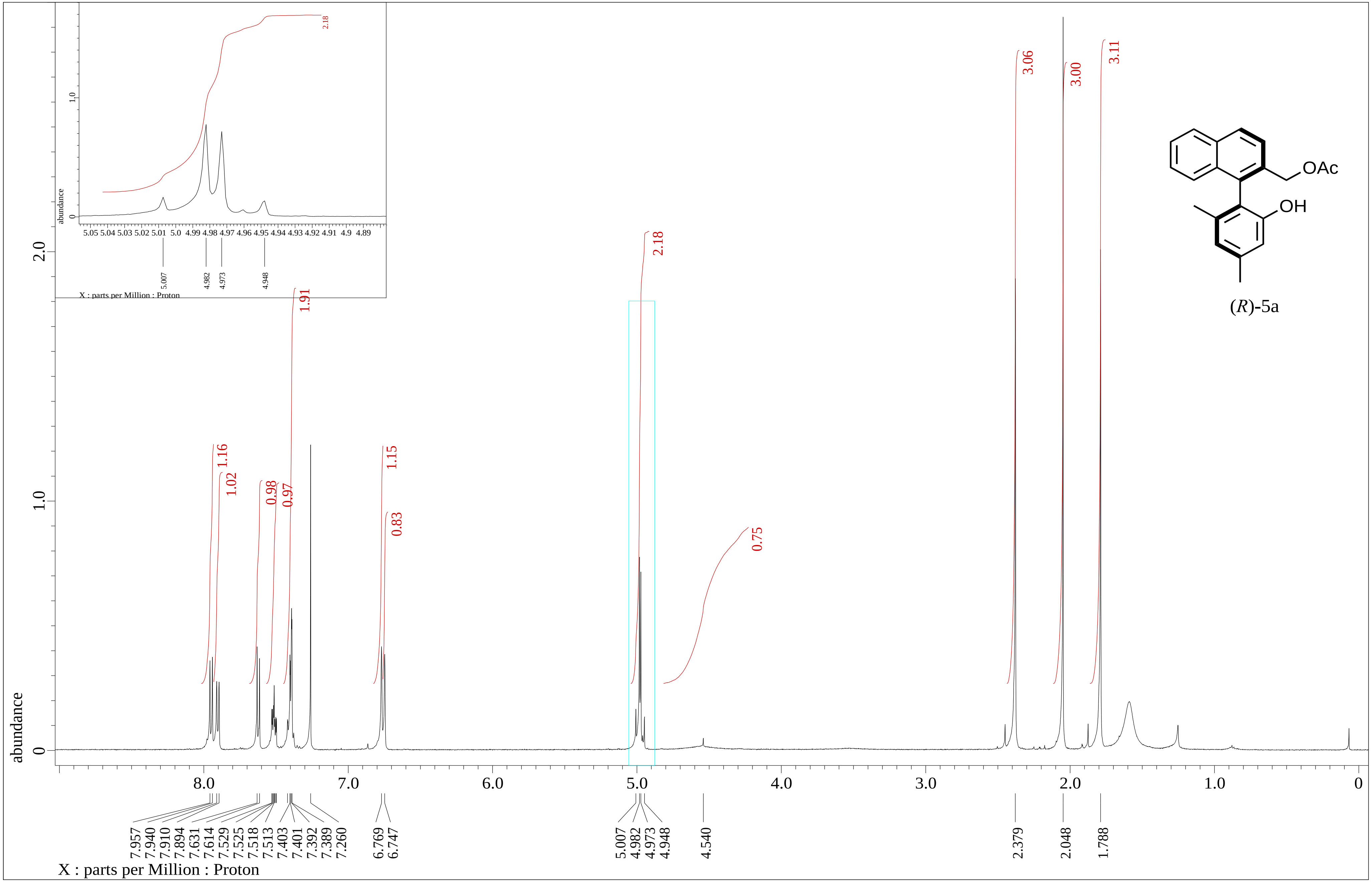

^{1}H NMR spectrum (500 MHz, $CDCl_3$) of (*R*)-**5a**

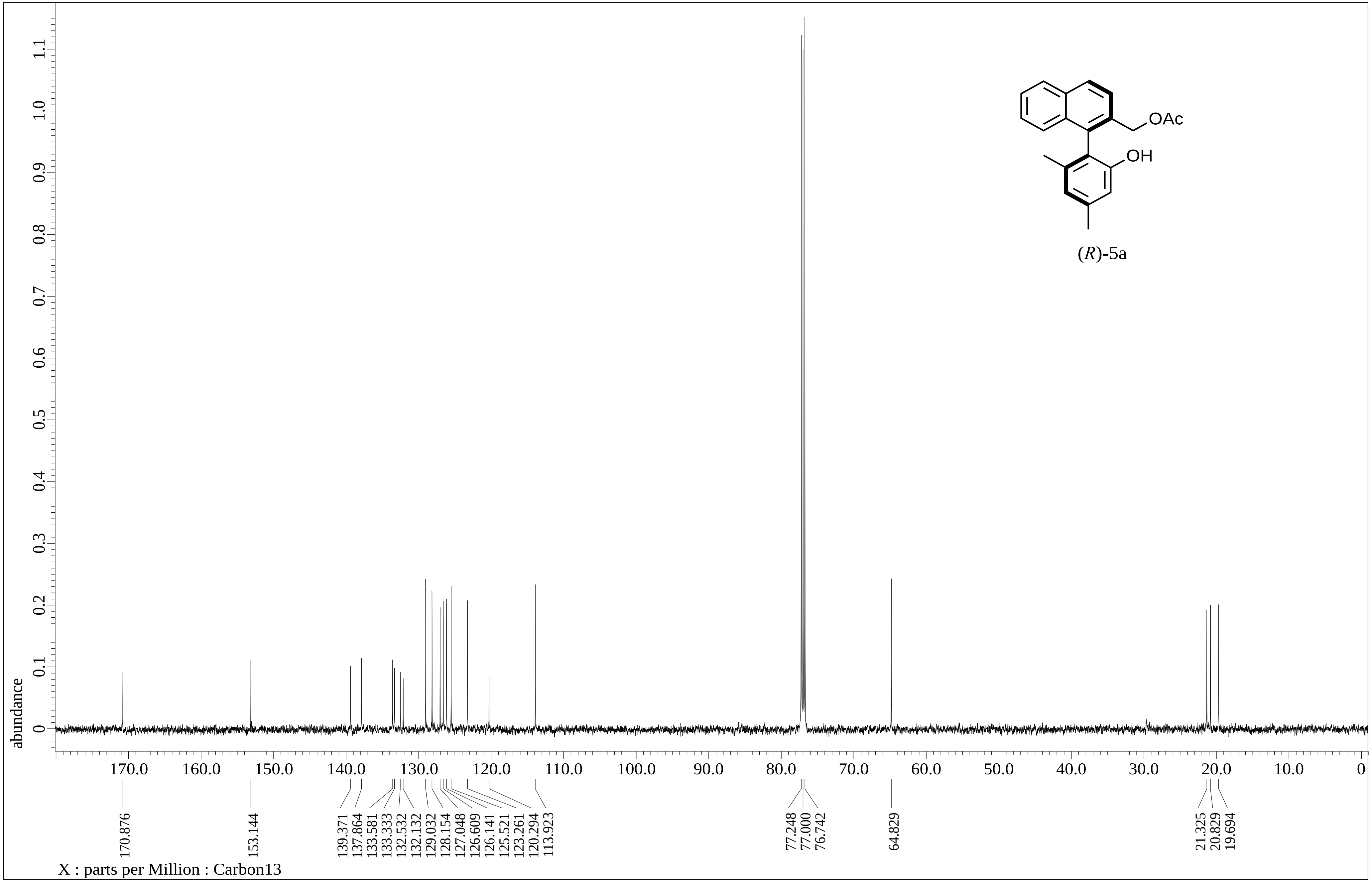

^{13}C NMR spectrum (125 MHz, $CDCl_3$) of (*R*)-**5a**

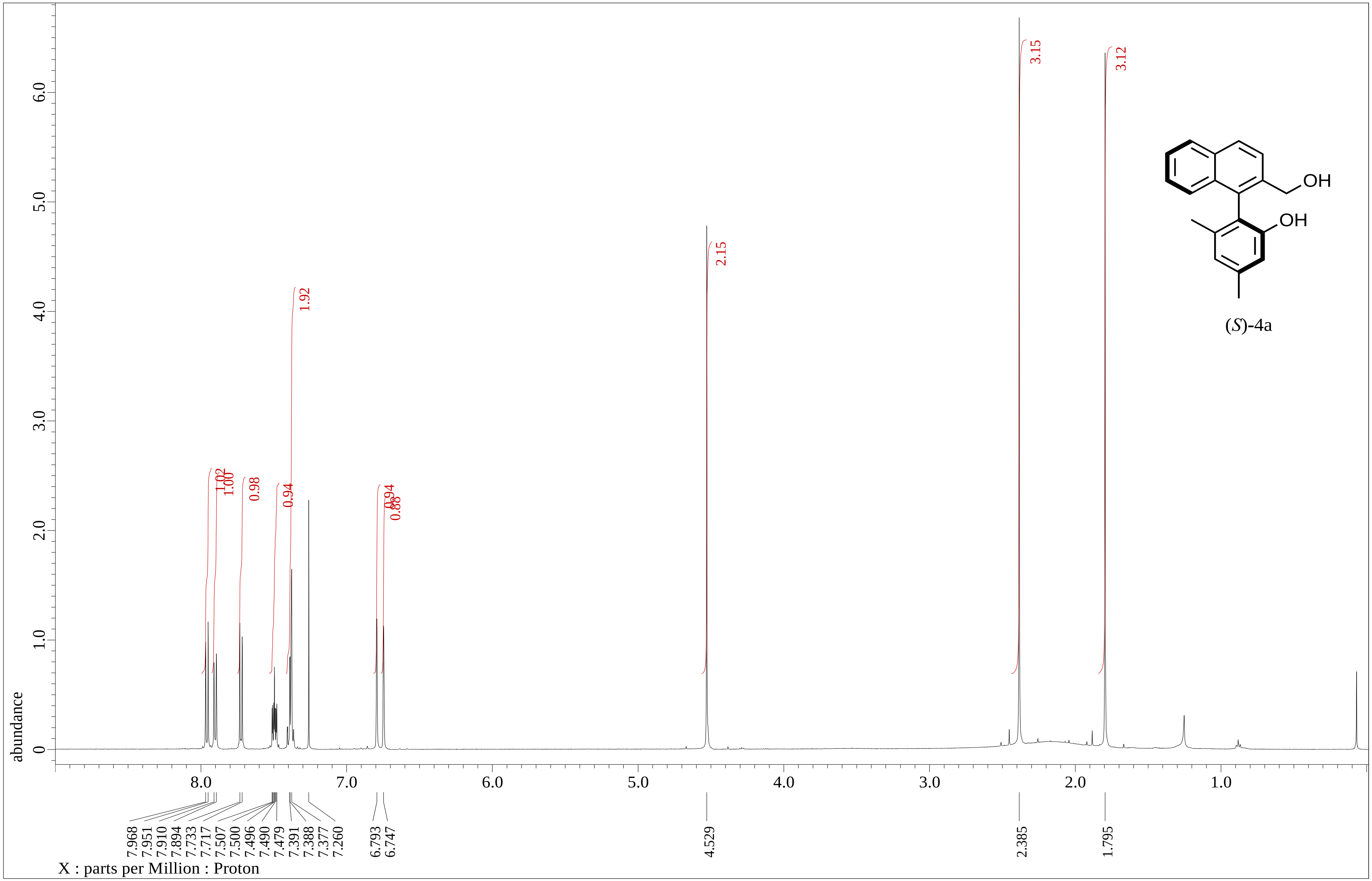

^{1}H NMR spectrum (400 MHz, $CDCl_3$) of (*S*)-**4a**

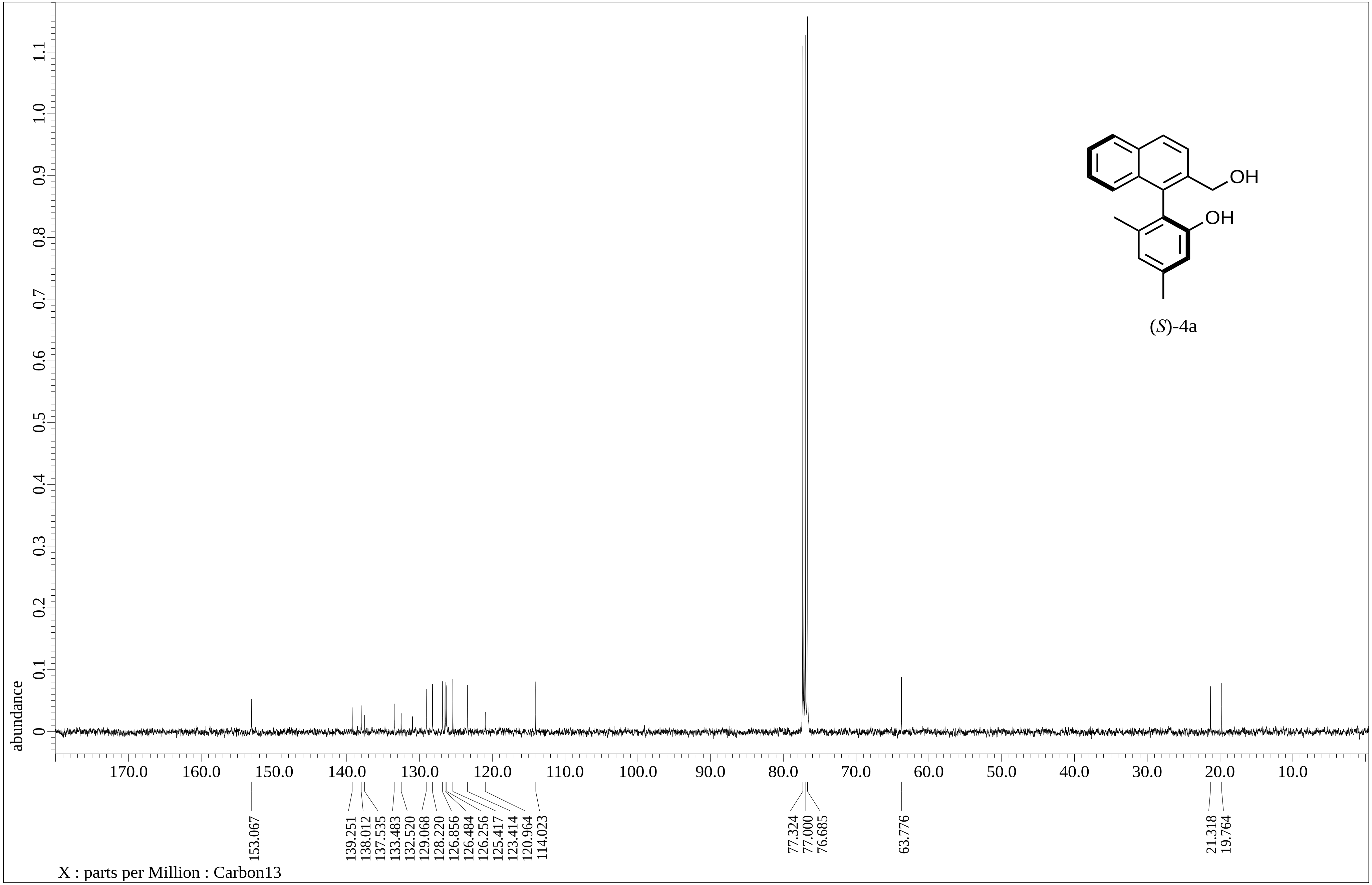

^{13}C NMR spectrum (100 MHz, $CDCl_3$) of (*S*)-**4a**

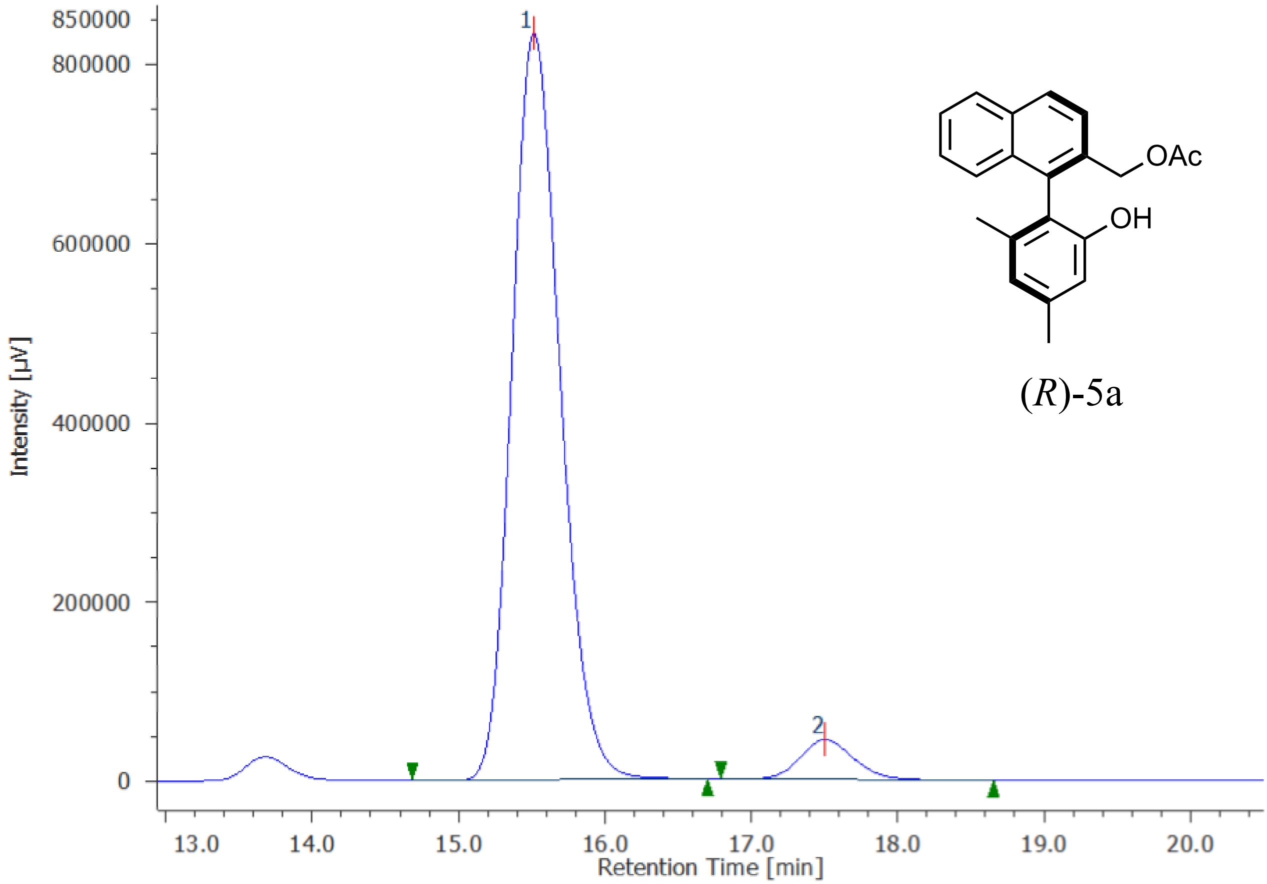

#	ピーク名	CH	tR [min]	面積 [µV·sec]	高さ [µV]	面積%	高さ%	定量値	NTP	分離度	シンメトリー係数	警告
1	Unknown	9	15.513	19103734	832754	94.470	94.955	N/A	10601	3.166	1.189	
2	Unknown	9	17.503	1118358	44249	5.530	5.045	N/A	11325	N/A	1.109	

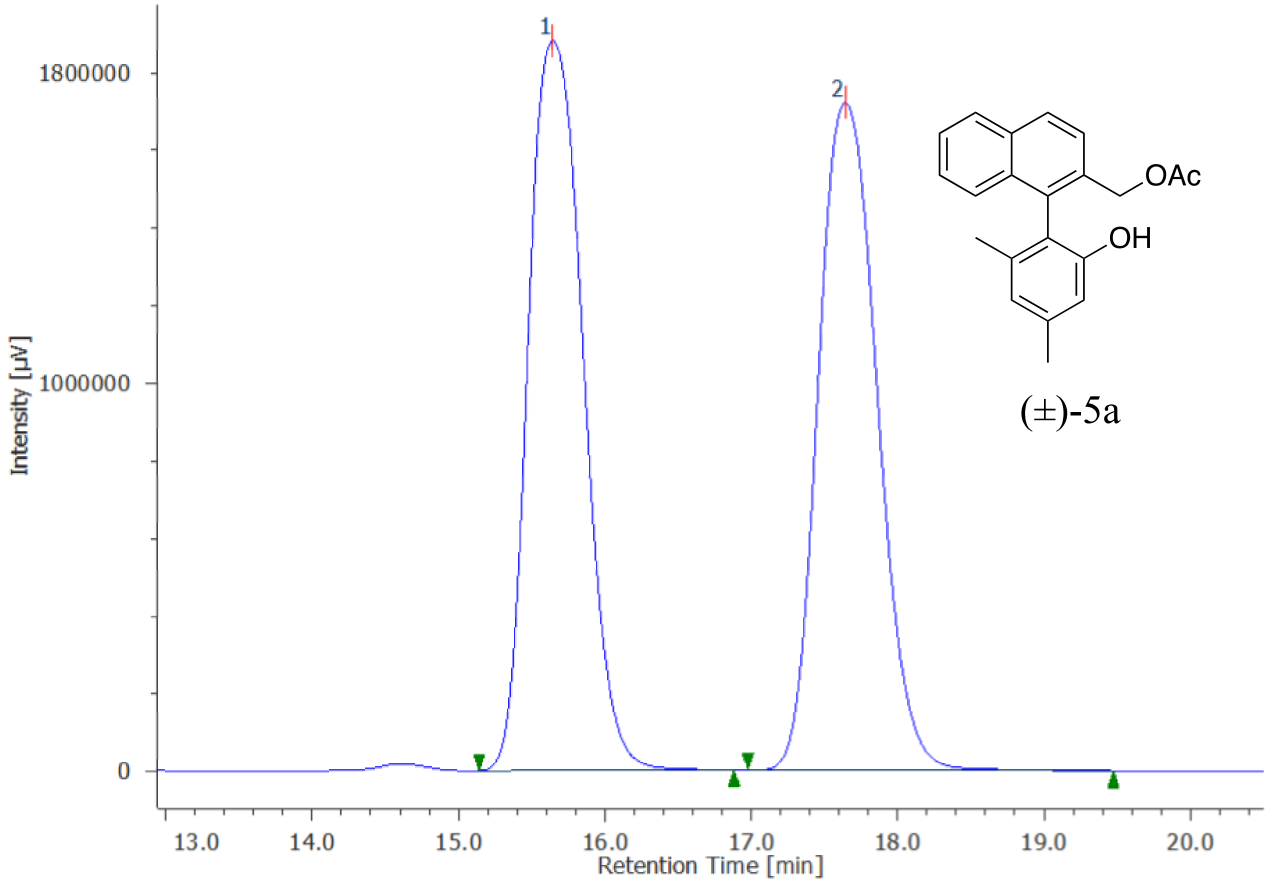

#	ピーク名	CH	tR [min]	面積 [µV·sec]	高さ [µV]	面積%	高さ%	定量値	NTP	分離度	シンメトリー係数	警告
1	Unknown	9	15.643	48950183	1879216	50.307	52.239	N/A	7946	2.757	1.195	
2	Unknown	9	17.643	48351924	1718137	49.693	47.761	N/A	8786	N/A	1.159	

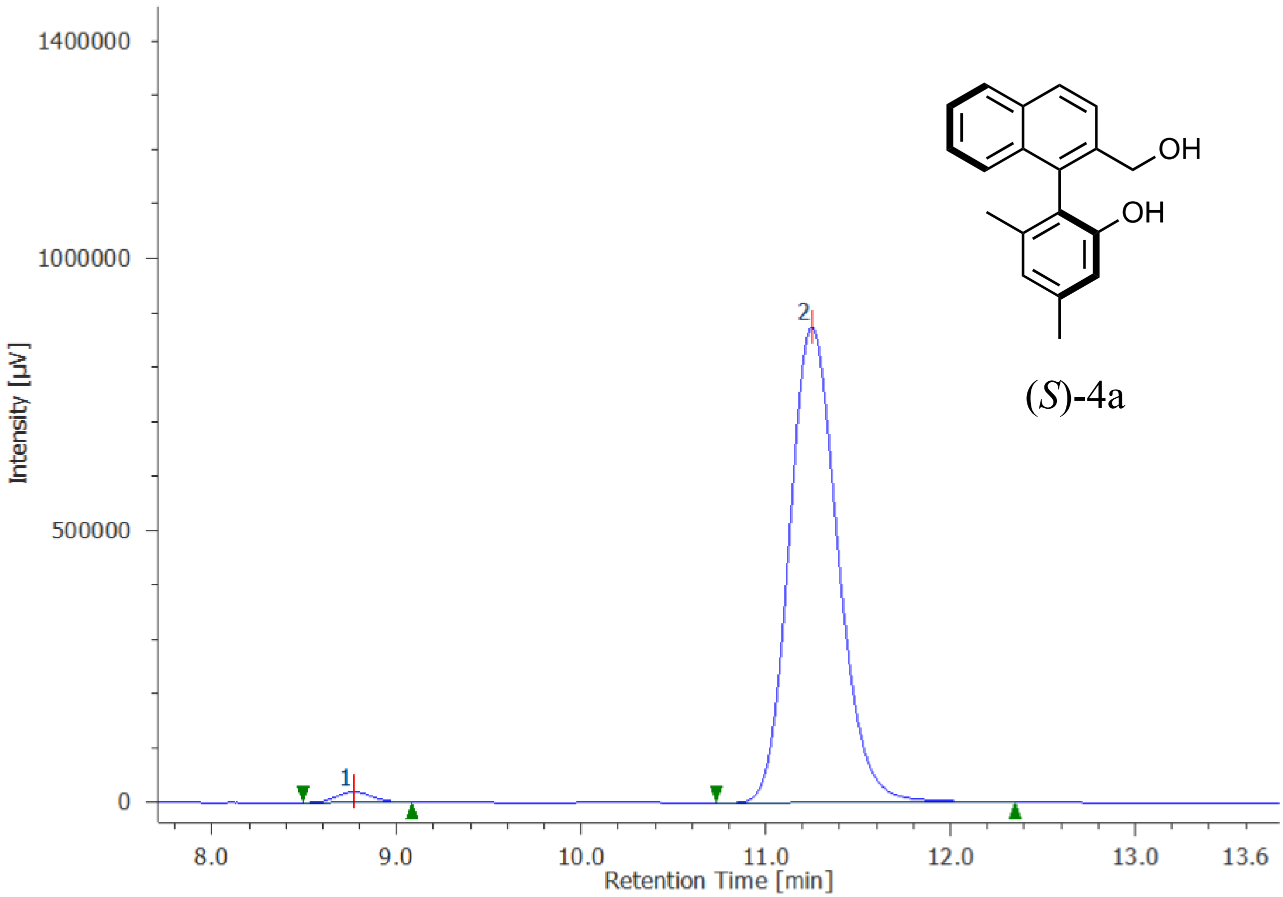

#	ピーク名	CH	tR [min]	面積 [µV·sec]	高さ [µV]	面積%	高さ%	定量値	NTP	分離度	シンメトリー係数	警告
1	Unknown	9	8.767	256123	19129	1.573	2.141	N/A	9584	5.950	1.095	
2	Unknown	9	11.247	16027587	874469	98.427	97.859	N/A	8870	N/A	1.167	

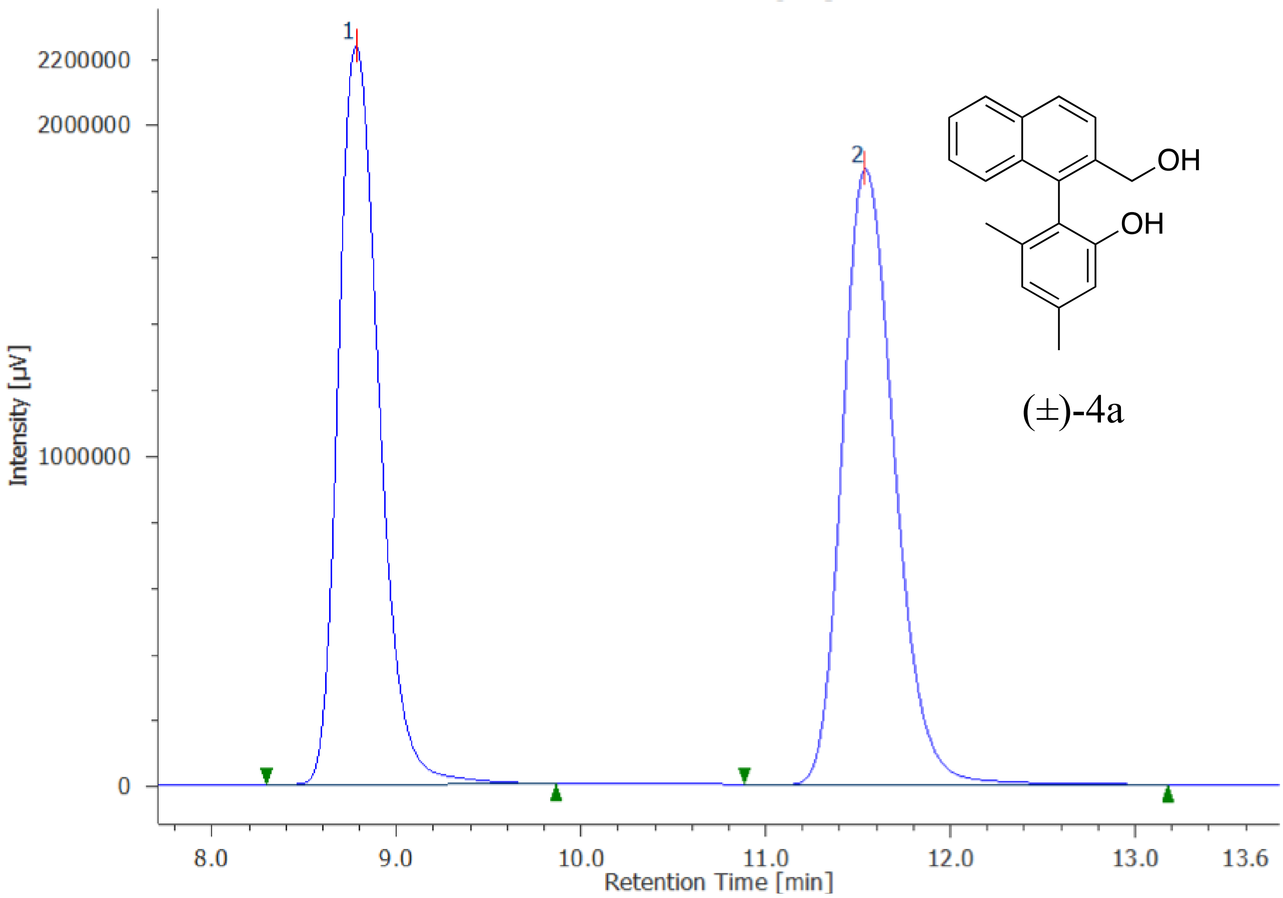

#	ピーク名	CH	tR [min]	面積 [µV·sec]	高さ [µV]	面積%	高さ%	定量値	NTP	分離度	シンメトリー係数	警告
1	Unknown	9	8.780	34492473	2236135	48.547	54.556	N/A	7752	6.076	1.275	
2	Unknown	9	11.537	36557345	1862678	51.453	45.444	N/A	8160	N/A	1.237	

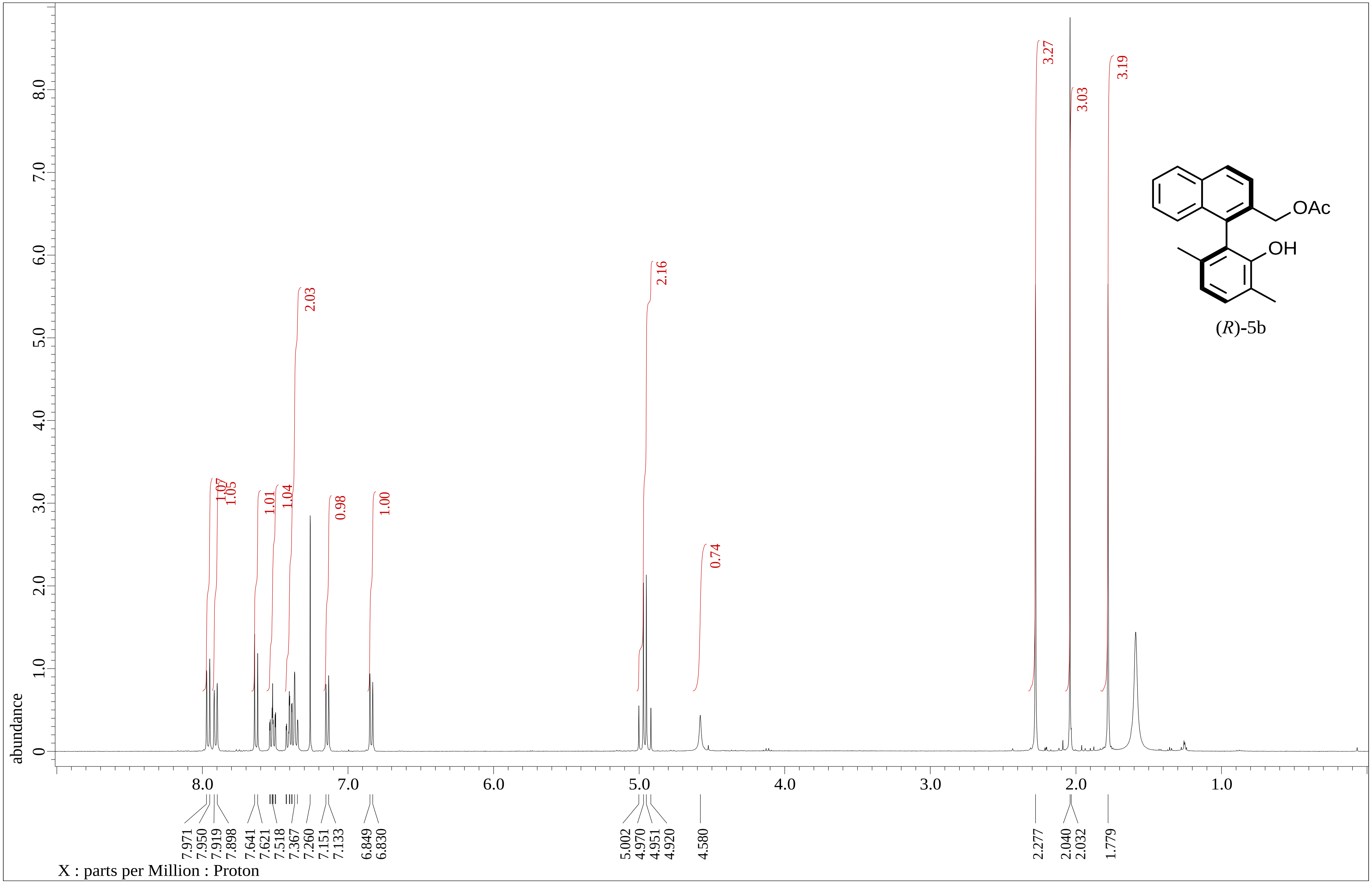

^{1}H NMR spectrum (400 MHz, $CDCl_3$) of (*R*)-**5b**

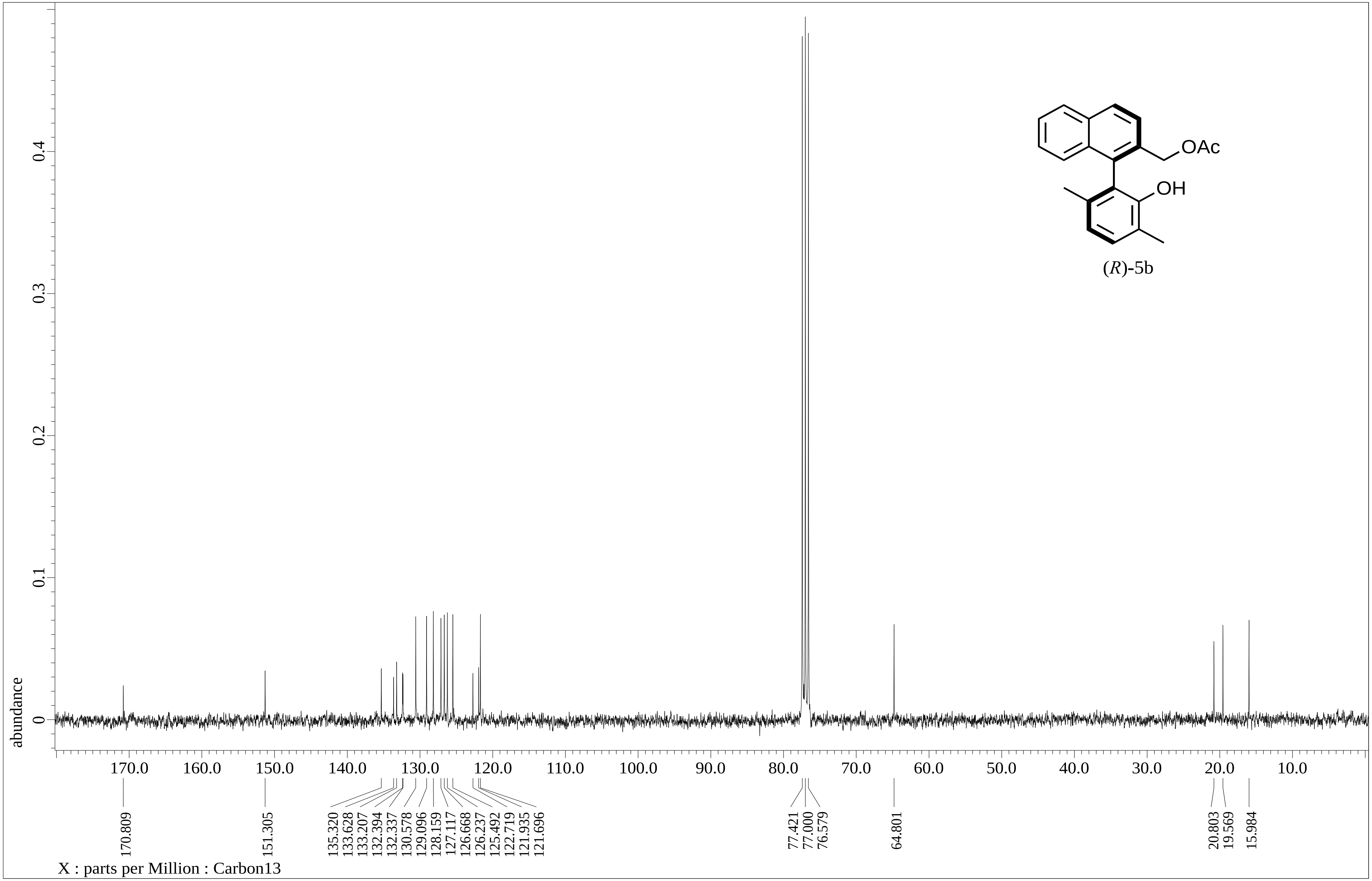

^{13}C NMR spectrum (100 MHz, $CDCl_3$) of (*R*)-**5b**

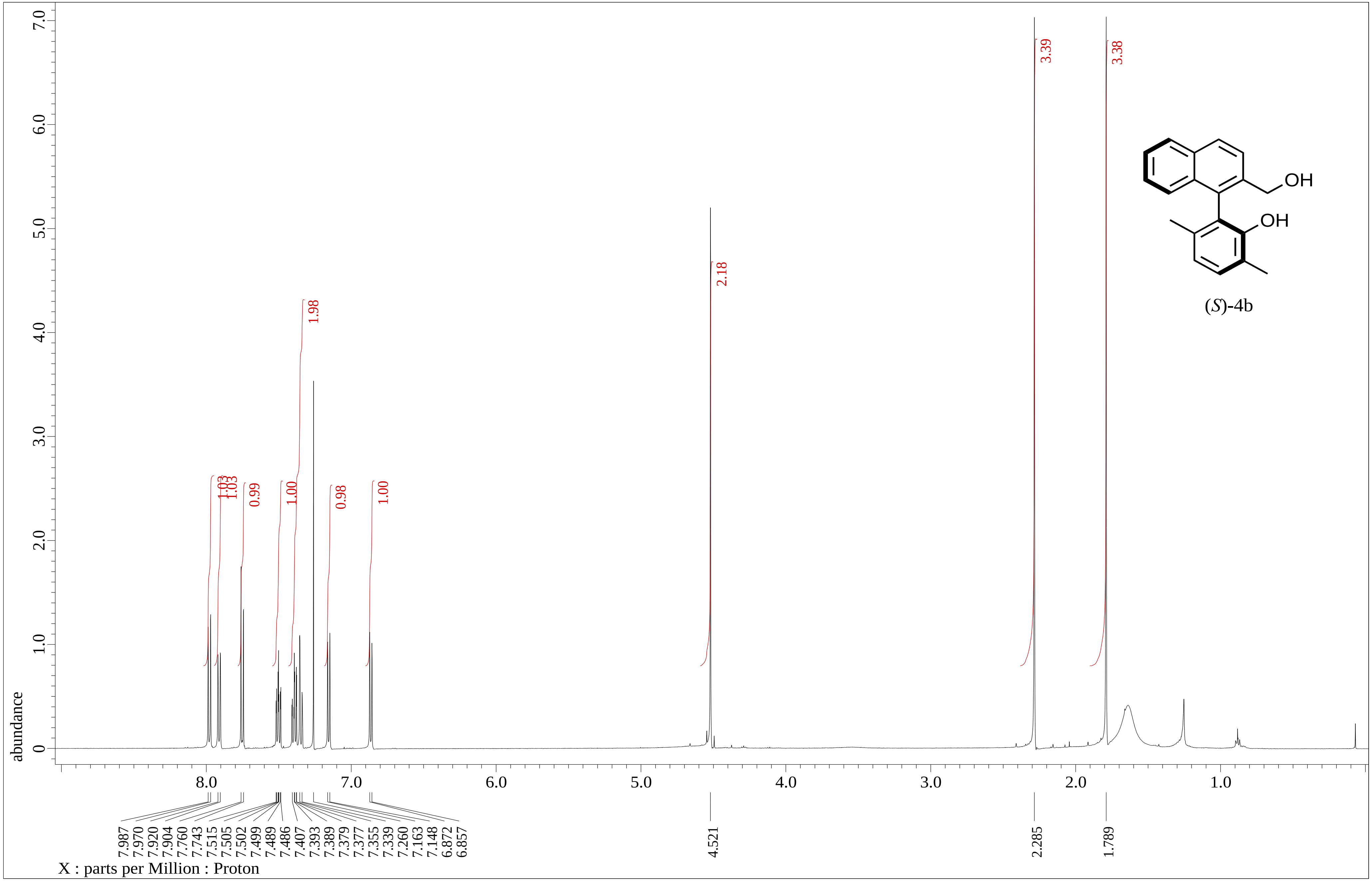

^{1}H NMR spectrum (400 MHz, $CDCl_3$) of (*S*)-**4b**

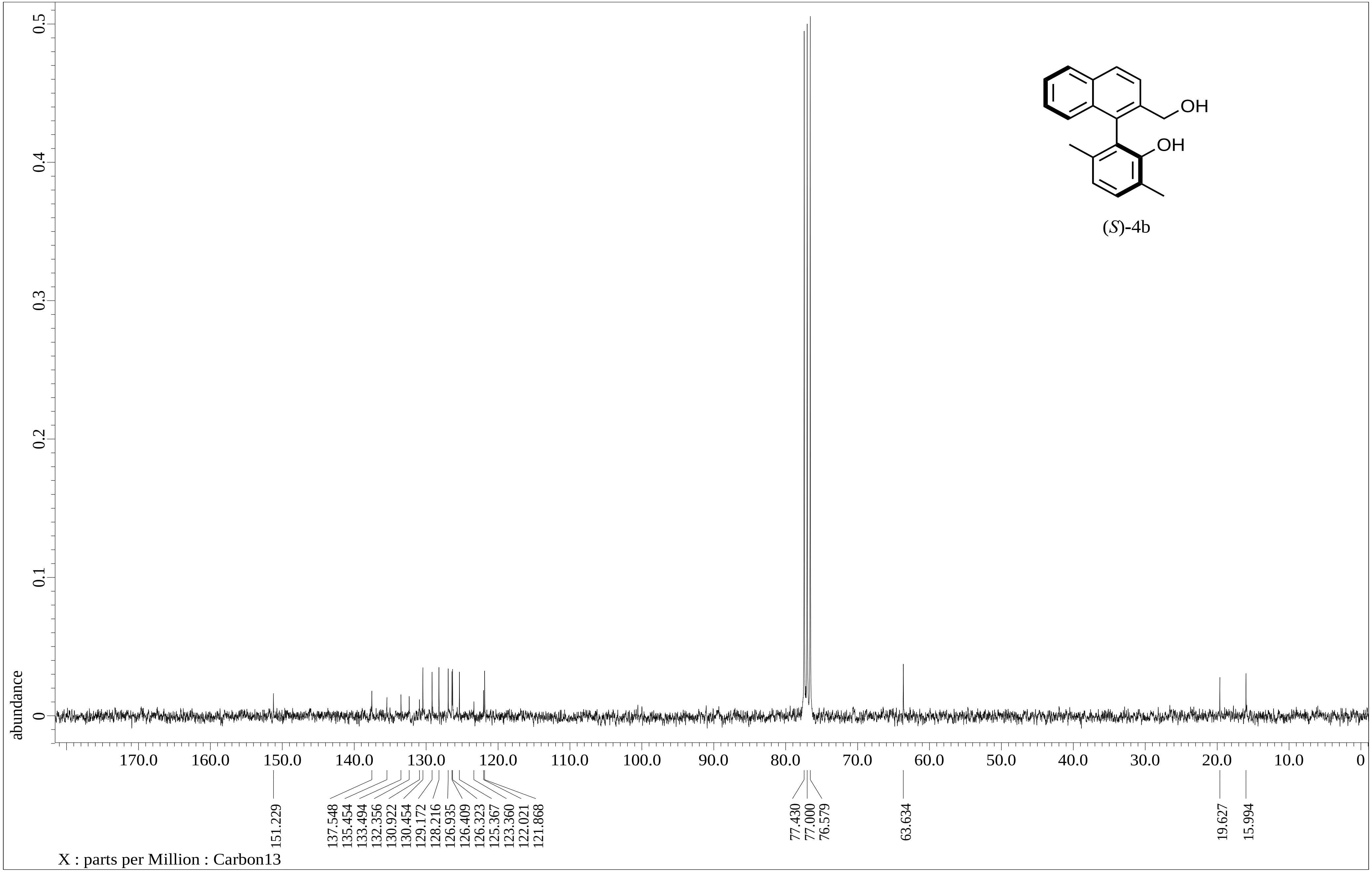

^{13}C NMR spectrum (100 MHz, $CDCl_3$) of (*S*)-**4b**

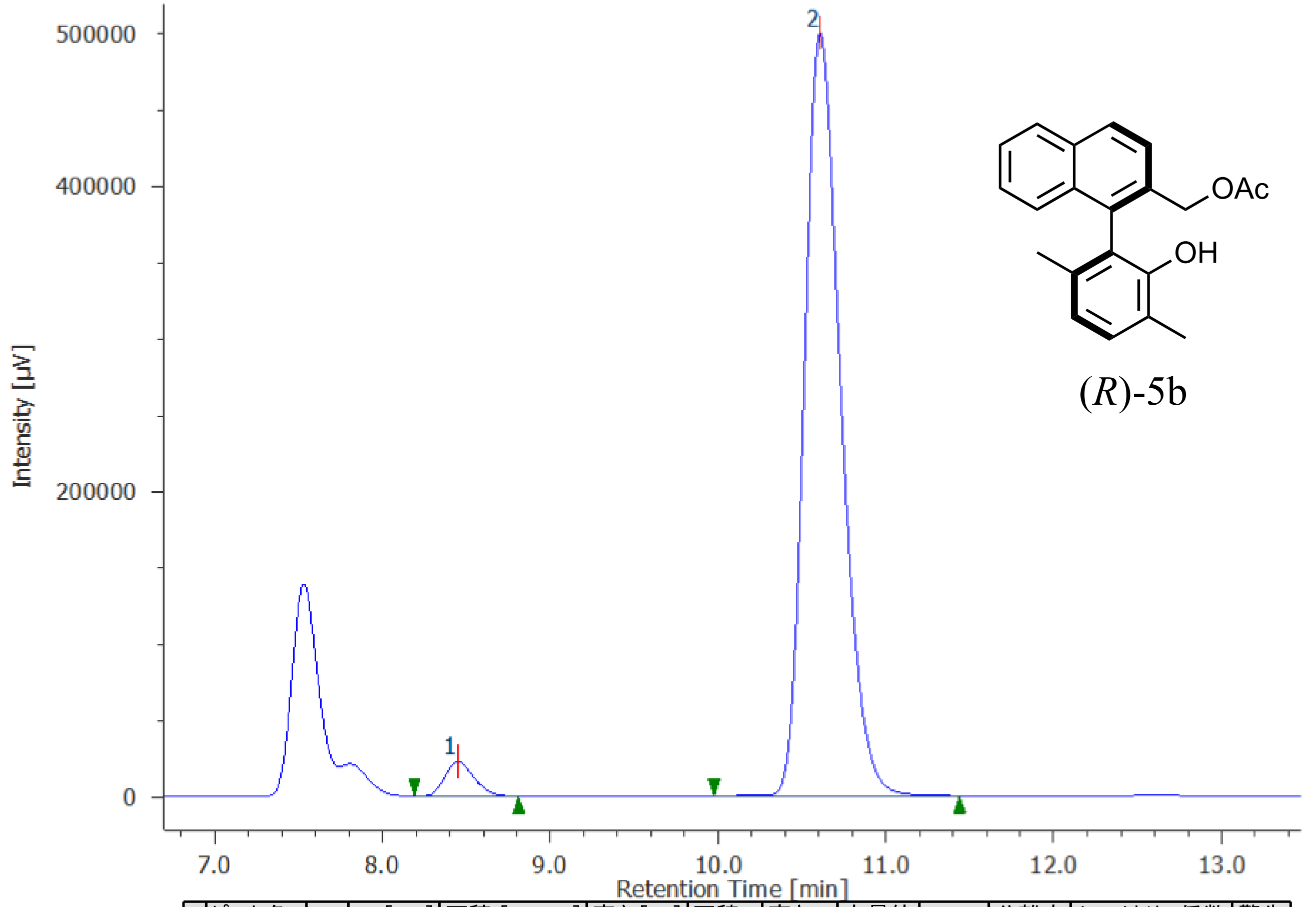

#	ピーク名	CH	tR [min]	面積 [µV·sec]	高さ [µV]	面積%	高さ%	定量値	NTP	分離度	シンメトリー係数	警告
1	Unknown	9	8.447	276228	22965	3.548	4.390	N/A	11512	6.132	1.230	
2	Unknown	9	10.607	7509485	500197	96.452	95.610	N/A	11744	N/A	1.276	

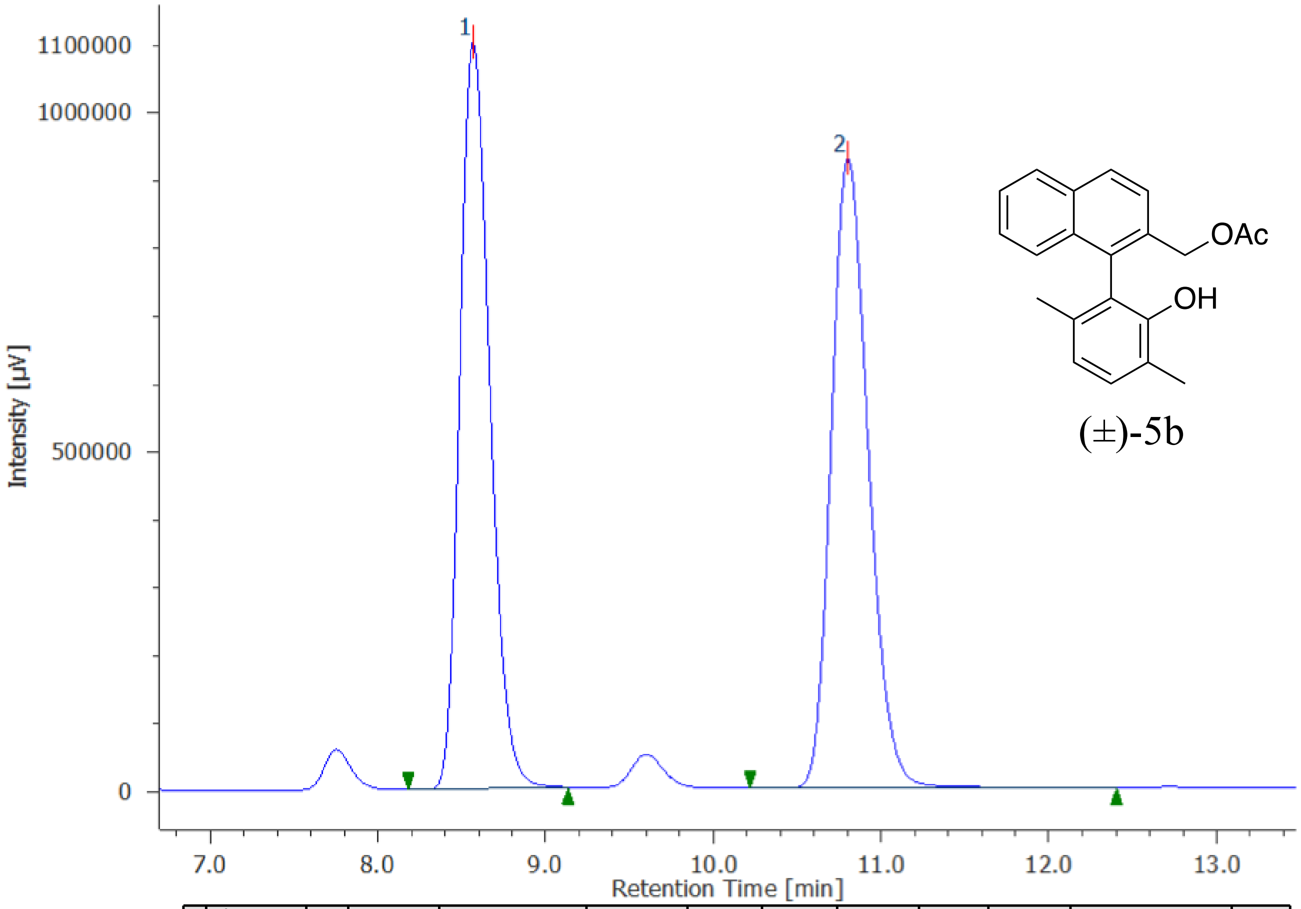

#	ピーク名	CH	tR [min]	面積 [µV·sec]	高さ [µV]	面積%	高さ%	定量値	NTP	分離度	シンメトリー係数	警告
1	Unknown	9	8.567	13687664	1099721	48.969	54.245	N/A	10985	6.140	1.227	
2	Unknown	9	10.800	14264005	927612	51.031	45.755	N/A	11519	N/A	1.216	

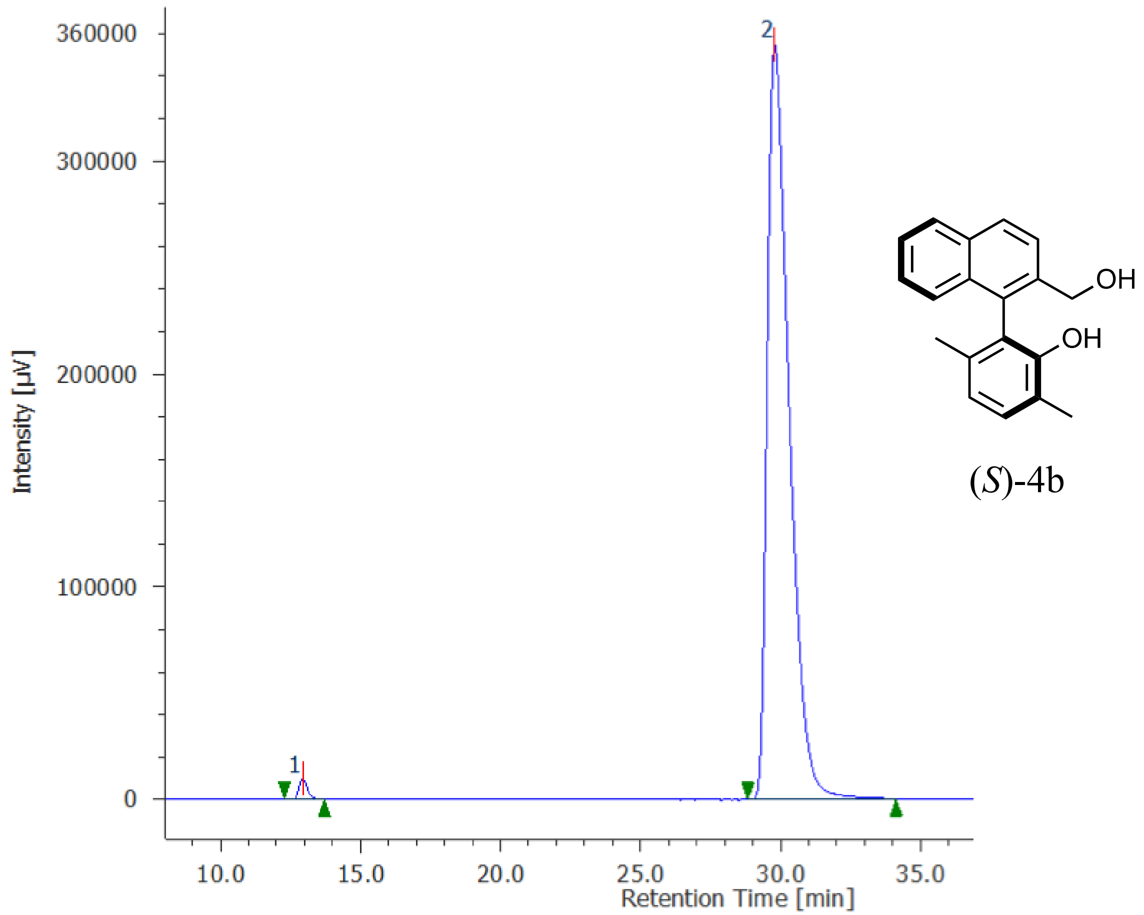

#	ピーク名	CH	tR [min]	面積 [µV·sec]	高さ [µV]	面積%	高さ%	定量値	NTP	分離度	シンメトリー係数	警告
1	Unknown	9	12.933	195818	9737	1.006	2.671	N/A	9922	17.513	1.271	
2	Unknown	9	29.787	19263402	354842	98.994	97.329	N/A	7136	N/A	1.727	

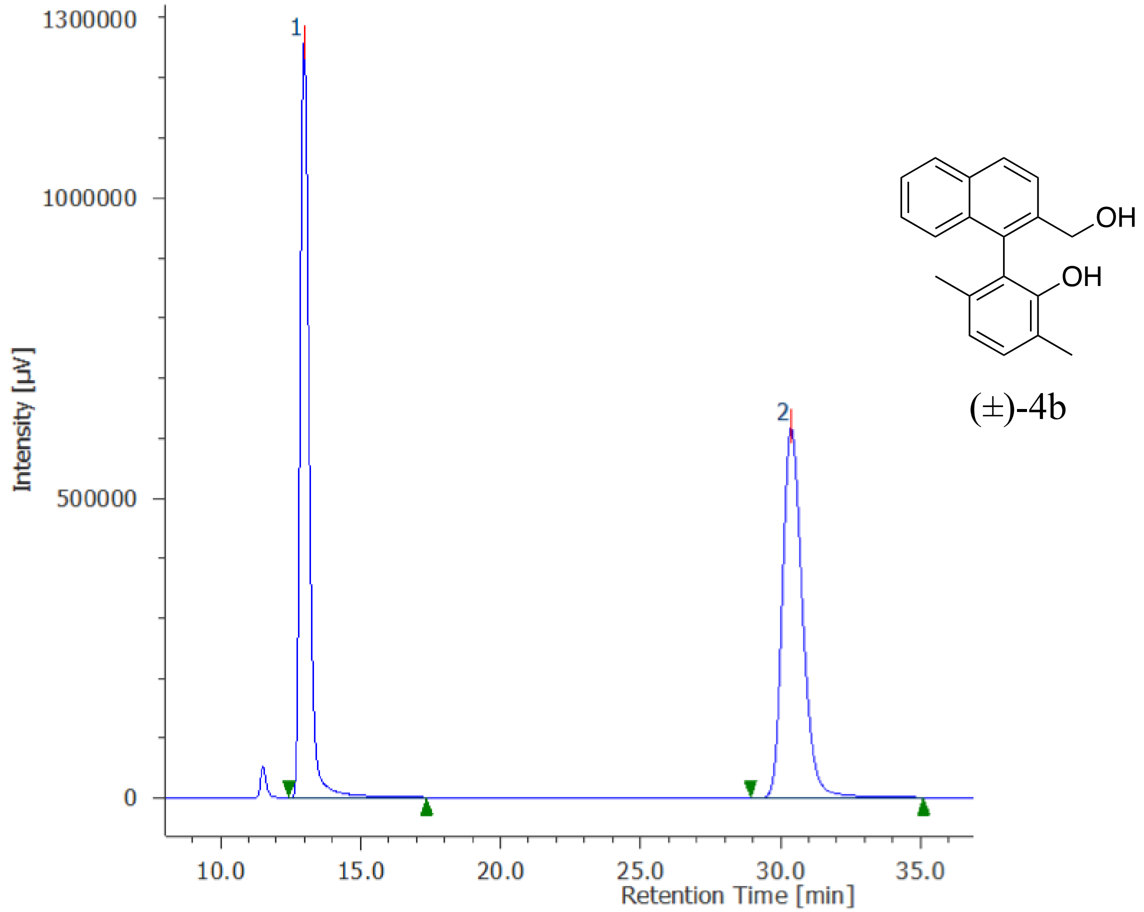

#	ピーク名	CH	tR [min]	面積 [µV·sec]	高さ [µV]	面積%	高さ%	定量値	NTP	分離度	シンメトリー係数	警告
1	Unknown	9	12.980	28267262	1258238	47.991	67.105	N/A	8505	18.938	1.316	
2	Unknown	9	30.373	30633675	616782	52.009	32.895	N/A	9026	N/A	1.260	
